# Cohesin reshapes replication fork contacts to aid fork slowing and reversal

**DOI:** 10.1038/s41586-026-11034-0

**Published:** 2026-09-16

**Authors:** Daniel González-Acosta, Daniel Giménez-Llorente, Melani Rodrigues, Moses Aouami, Orhi Barroso-Gomila, Javier Muñoz, Ana Cuadrado, Ana Losada, Massimo Lopes

**Affiliations:** 1 Institute of Molecular Cancer Research (IMCR), University of Zurich, Zurich, Switzerland; 2 Chromosome Dynamics Group, Spanish National Cancer Research Centre (CNIO), Madrid, Spain; 3 Cell Signaling and Clinical Proteomics Group, Biobizkaia Health Research Institute, Barakaldo, Spain; 4 Ikerbasque, the Basque Foundation for Science, Bilbao, Spain

**Keywords:** Stalled forks, DNA damage response

## Abstract

DNA replication forks can be challenged by cancer chemotherapeutic treatments, leading to accumulation of single-stranded DNA and slowdown of DNA synthesis. The marked plasticity of replication forks under replication stress ensures fork stability, damage tolerance and complete genome duplication^1^. Initiation and progression of replication forks occur in a three-dimensionally organized genome. DNA loop extrusion by the cohesin complex organizes the genome^2^ and regulates the initiation and positioning of DNA replication origins^3,4^. Although transient interaction of sister forks was recently reported during unperturbed replication^5^, the functional relevance of fork contacts during replication stress and the role of cohesin in this context remain unknown. Here we show that cohesin-mediated loop extrusion rearranges nascent DNA contacts at stressed replication forks to promote genome stability. Using auxin-inducible degron^6^, separation-of-function mutants^7–9^ and a newly developed Micro-C-based technique to capture chromatin contacts at nascent DNA (Repli-C), we found that loop-extruding cohesin accumulates at stalled replication forks, limiting sister-fork coupling in favour of inter-replicon contacts. This process promotes active fork slowing and reversal by preventing PRIMPOL action on single-stranded DNA^1^. These findings show that the replication stress response is not merely an accumulation of individual regulatory events, but is topologically integrated across the genome through cohesin loop extrusion. While providing a new function for loop-extruding cohesin, our results indicate the potential impact on cancer therapy of frequent cohesin mutations in tumours^10^.

## Abstract (web summary)

Cohesin-mediated loop extrusion limits sister-fork coupling and tethers nearby replication forks under replication stress, promoting fork reversal and slowing fork progression to safeguard genome stability.

## Main

Replication of organized genomes is spatiotemporally regulated, in which topologically associating domains (TADs) containing marks of open chromatin and active transcription replicate earlier than compact, transcriptionally repressed TADs^11,12^. Initiation of DNA replication takes place at DNA replication origins that spread along the genome^13,14^, which are enriched in initiation zones, defined by population studies^15^. Recent replication-specific Hi-C analyses revealed nascent DNA contacts within the same initiation zone, possibly representing sister forks of the same replicon and/or converging forks from neighbouring replicons, which are physically coupled during DNA synthesis^5^. However, how nascent DNA organization is affected by conditions interfering with fork progression (that is, replication stress (RS)) remains unknown. RS triggers changes in the composition of the replication apparatus and in the architecture of replication forks. This ‘fork plasticity’ assists cells tolerating DNA damage and replication interference^1,11^. One such mechanism is fork reversal, the active and reversible remodelling of the replication fork into a four-way junction through annealing of the two nascent DNA strands. This requires RAD51 and the DNA translocases SMARCAL1, HLTF and ZRANB3. An alternative mechanism sustaining DNA synthesis under stress is repriming by the DNA primase and polymerase PRIMPOL to promote discontinuous DNA synthesis^1^. How these mechanisms are used locally and globally remains unknown and may reflect chromatin architecture and the accessibility of nascent DNA to different stress-tolerance factors.

The cohesin complex (SMC1, SMC3, RAD21 and either STAG1 or STAG2) organizes the genome into TADs and chromatin loops because of its ability to extrude DNA^16–18^. This function requires its association with the cohesin chromatin loader NIPBL–MAU2 (refs. ^2,19^). Loop-extruding cohesin is stopped at certain locations by protein barriers, such as CTCF, or the MCM complex belonging to the replicative CMG helicase (CDC45–MCM–GINS)^20,21^. Cohesin also interacts with replicative pre-initiation complexes and aids localizing replication origins and initiation zones at early replicating domains^3,4^. During DNA replication, different mechanisms to embrace both sister chromatids by cohesin (cohesion) have been proposed and possibly coexist. These include replication fork passage through the ring, transient ring opening or cohesin conversion at replication termination events^22,23^. Binding of Sororin to cohesin complexes promotes cohesion by preventing cohesin unloading by WAPL^24^. In yeast, the MRX complex promotes cohesin recruitment to stalled replication forks aiding their stability and restart^25–27^. However, mechanisms of fork stability differ greatly between yeast and human cells. Although fork reversal in yeast is mainly found in checkpoint-deficient mutants, in human cells it evolved as one of the most frequent physiological responses at stressed replication forks, assisting genome stability^1^. Moreover, yeast cells do not possess PRIMPOL and mainly use Polα primase for this purpose^28,29^. In human cells, the cohesin complex rapidly accumulates at stalled replication forks^30^, and excessive accumulation due to unloading failure impairs replication fork progression^31,32^. However, whether physiological levels of cohesin actively restrain fork progression upon RS remained unknown. In this study, we characterized the molecular functions and dependencies of cohesin stabilization at stalled replication forks. We describe how cohesin reshapes replicating domains and drives fork plasticity, mediating the cellular response to genotoxic agents.

## Cohesin binds stalled forks through NIPBL

**Fig. 1:**
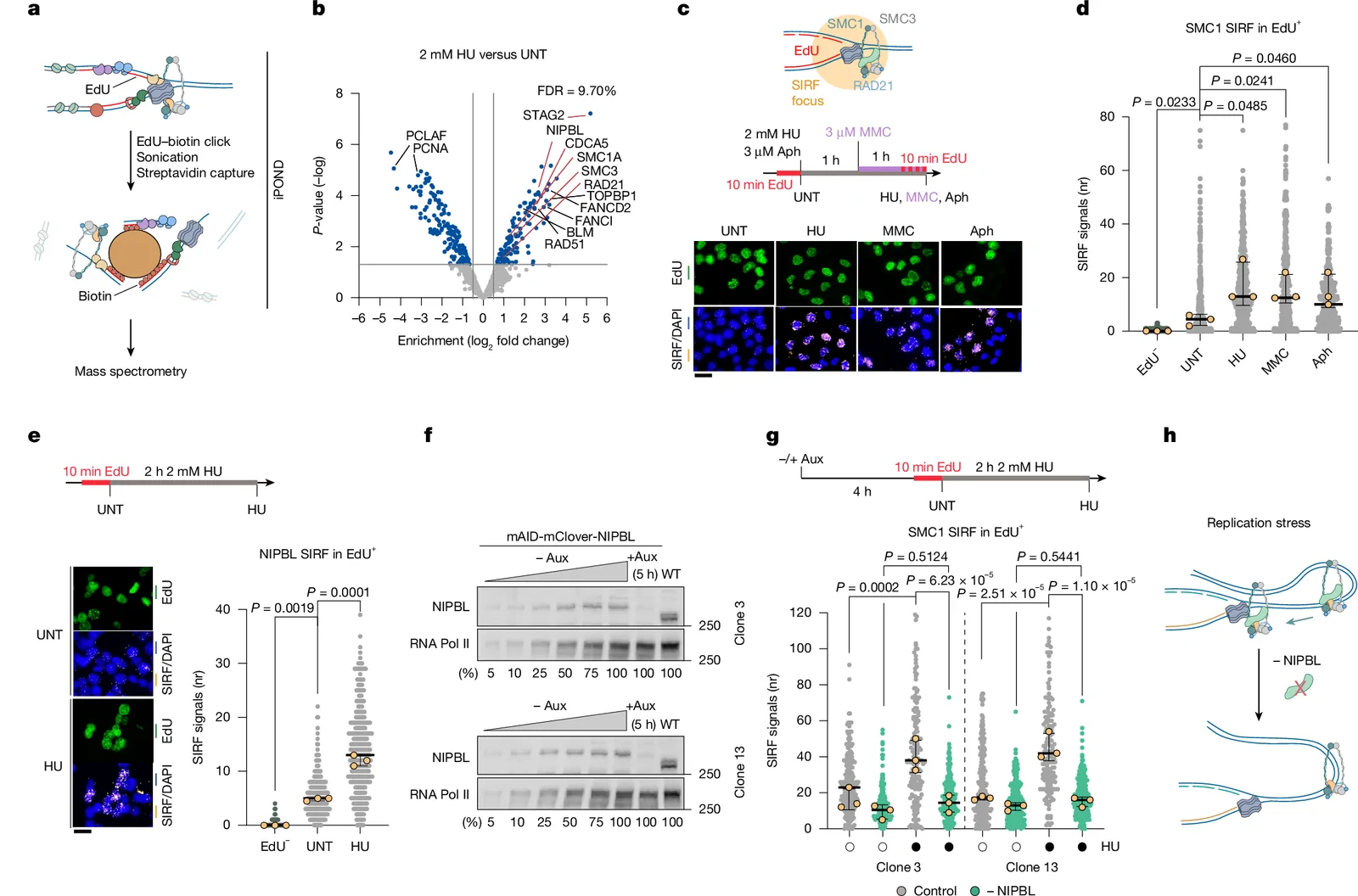
Cohesin accumulates at stalled replication forks in a NIPBL-dependent manner. **a**, iPOND–MS approach. Nascent DNA is clicked to biotin. DNA is sonicated and nascent DNA captured with streptavidin beads. Associated proteins are identified by mass spectrometry. **b**, Volcano plot of iPOND–MS data showing enrichment (log_2_ fold change) and significance (−log *P*-value) of proteins in 2 mM HU compared with untreated conditions (UNT). Among the selected proteins, cohesin subunits are indicated by red lines. FDR for selected comparison at *P* = 0.05 is shown. Differential abundance analysis. LIMMA test with 0.5 log_2_ fold change threshold. Benjamini–Hochberg correction for *P*-values and FDR. **c**, SMC1 SIRF. EdU was added for 10 min before HU or Aph, or in the last 10 min of MMC treatment. **d**, Quantification of **c**. EdU^−^ cells from the UNT are shown as control. One-way analysis of variance (ANOVA) plus Bonferroni with exact *P*-values. *n* = 3 median values (error bars, ±s.d.) of biological replicates (orange dots). Distribution values and median (black line) from one replicate are shown. **e**, NIPBL SIRF after 2 mM HU. One-way ANOVA plus Bonferroni with exact *P*-values. *n* = 3 median values (error bars, ±s.d.) of biological replicates (orange dots). Distribution values and median (black line) from one replicate are shown. **f**, Immunoblot of NIPBL and RNA Pol II (loading control) in mAID-mClover-NIPBL clones 3 and 13. Experiment repeated three times with similar results. Marker bands with molecular weight (in kDa) are shown. For gel source data, see Supplementary Fig. 1. **g**, SMC1 SIRF in NIPBL-degron cells after 2 mM HU. One-way ANOVA plus Bonferroni with exact *P*-values. *n* = 3 median values (error bars, ±s.d.) of biological replicates (orange dots). Distribution values and median (black line) from one replicate are shown. Black circles indicate the addition of HU; white circles indicate that HU is absent. **h**, Effect of NIPBL removal on cohesin at stalled forks. Scale bar, 20 μm (**c**,**e**). Source data

**Extended Data Fig. 1:**
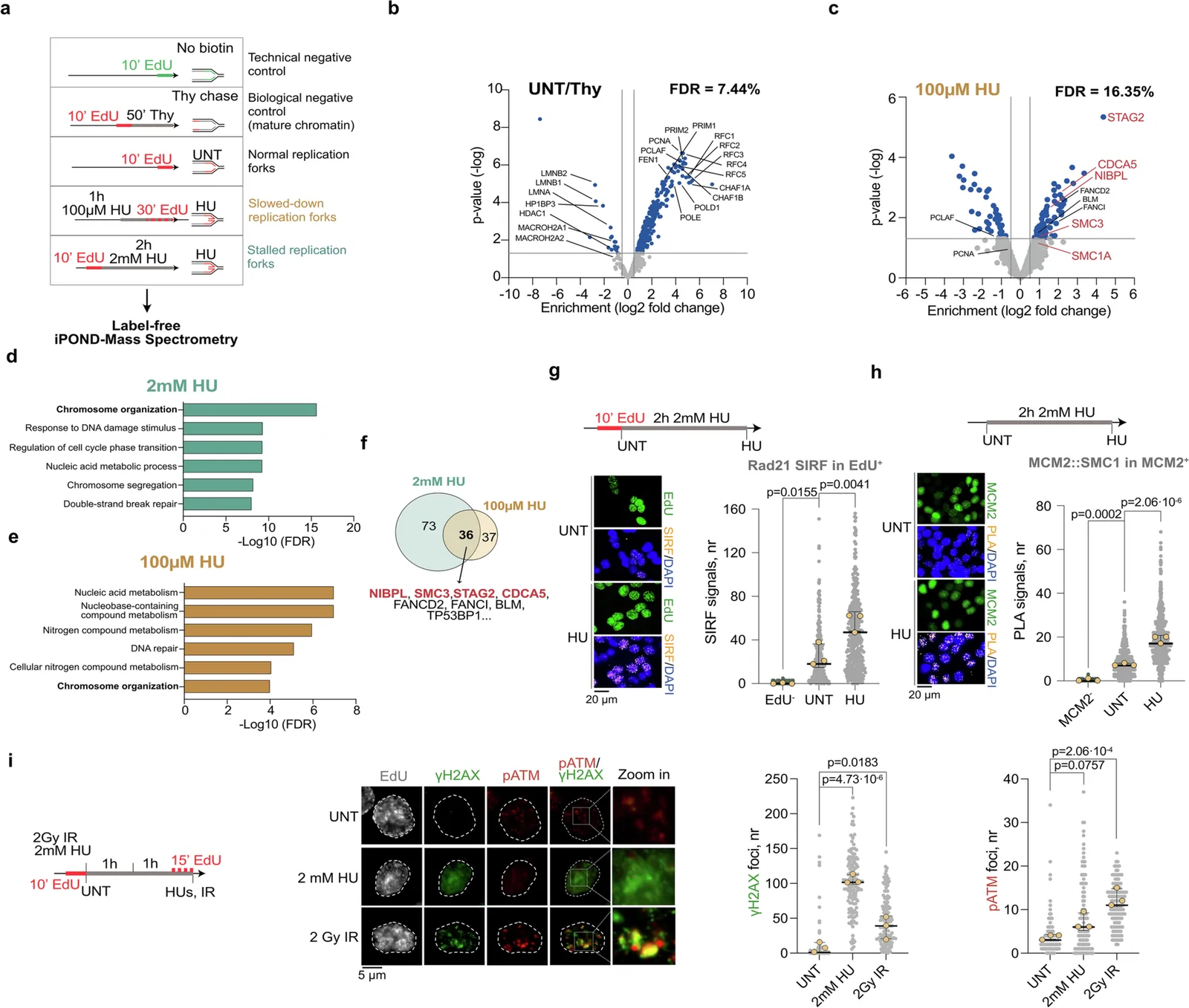
iPOND-MS analyses under fork slowing and stalling conditions with PLA validations. (**a**). Experimental conditions of iPOND-MS experiment. (**b** and **c**). Volcano plot of iPOND-MS data showing enrichment (log2 fold change) and significance (-log p-value) of the different proteins in untreated control compared to Thy chase (B) or to 100 µM HU (C). Selected protein hits are indicated. Cohesin subunits are shown in red. FDR for the selected comparison at p-value 0.05 is shown. Differential abundance analysis. LIMMA test with 0.5 log2 fold change (FC) threshold. Benjamini-Hochberg correction for p-values and FDR. (**d** and **e**). PANTHER Gene Ontology (GO) analyses of biological pathways in iPOND-MS hits of 2 mM HU and 100 µM HU. Significance is assessed with -Log10 FDR. (**f**). Venn diagram showing number of specific and overlapped protein hits found in 2 mM HU and 100 µM HU conditions. Examples protein hits found enriched in both are shown. (**g**). RAD21 SIRF upon 2 mM HU. EdU^−^ cells from the UNT condition are shown as a control. One-way ANOVA plus Bonferroni with exact p-values. n = 3 median values (error bars, +/− SD) of biological replicates (orange dots). Distribution values and median (black line) from one replicate are shown. (**h**). MCM2::SMC1 PLA after 2 mM HU. MCM2^-^ cells (G2 cells lacking MCM2 staining) from UNT serve as a negative control. One-way ANOVA plus Bonferroni with exact p-values. n = 3 median values (error bars, +/− SD) of biological replicates (orange dots). Distribution values and median (black line) from one replicate are shown. (**i**). γH2AX and pATM immunofluorescence staining and quantifications of cells treated with 2 mM HU or 2 Gy γ-irradiation. One-way ANOVA plus Bonferroni with exact p-values. n = 3 median values (error bars, +/− SD) of biological replicates (orange dots). Distribution values and median (black line) from one replicate are shown. Source data

Using ‘isolation of proteins on nascent DNA’ coupled to mass spectrometry^33^ (iPOND–MS; Fig. 1a), we characterized the proteome landscape of replication forks when cells experience two different treatments with the ribonucleotide reductase inhibitor hydroxyurea (HU): 2 h at 2 mM, which stalls all ongoing replication forks by severely depleting the cellular nucleotide pool^34^; and 1 h at 100 µM, which induces fork slowdown^35^ through mild reduction of cellular nucleotides and by promoting TIMELESS dissociation at forks through reactive oxygen species (ROS) and disruption of oligomerized PRDX2 (ref. ^36^). A thymidine chase control to enrich for mature, replicated chromatin-associated proteins (Thy) and a negative control, in which azide-biotin is substituted by DMSO, were also included (Extended Data Fig. 1a). As expected, mature, replicated chromatin is enriched in core histone proteins and lamins, whereas the untreated replication fork (UNT) is enriched in replisome core components (for example, POLE, POLD1, RFC1-5; ref. ^30^) (Extended Data Fig. 1b). After 2 h in 2 mM HU, we observed the enrichment of proteins involved in the RS response (for example, FANCD2/I, BLM, RAD51), in line with previous studies^30^ (Fig. 1b). Expectedly, fewer proteins were recruited to nascent DNA after 1 h at 100 µM HU (Extended Data Fig. 1c). Gene ontology analysis showed a high enrichment of factors involved in chromosome organization and other expected biological functions, such as DNA damage or cell cycle regulation factors (Extended Data Fig. 1d,e). Among those factors involved in chromosome organization, we found several subunits of the cohesin complex, especially enriched after 2 h at 2 mM HU (Fig. 1b). Importantly, following 1 h 100 µM and 2 h 2 mM HU treatments, STAG2, NIPBL, SMC3 and Sororin (CDCA5) were significantly enriched (Extended Data Fig. 1f). To support these results with an orthogonal assay, we used ‘in situ protein interactions with nascent DNA replication forks’ (SIRF), a proximity ligation-based assay (PLA) between cohesin and EdU-labelled DNA^37^ (Fig. 1c). We observed increased presence of SMC1 and RAD21 at nascent DNA after treatment with HU or with other fork stalling agents, for example, DNA polymerase α and δ inhibition by Aphidicolin (Aph) and a synthesis-permissive treatment with the DNA crosslinker Mitomycin C (MMC) (Fig. 1c,d and Extended Data Fig. 1g). Cohesin recruitment to nascent DNA was validated by proximity of SMC1 and the replisome component MCM2 (Extended Data Fig. 1h). We ruled out the possibility that double-strand breaks (DSBs) were responsible for cohesin accumulation at forks by analysing γH2AX (replication stress or DSB maker) and pATM foci (DSB signalling marker) in S-phase cells treated with 2 mM HU or γ-irradiation. Although γH2AX was strongly induced following 2 mM HU, we observed a mild, non-significant increase for pATM. By contrast, after γ-irradiation, both markers were significantly increased (Extended Data Fig. 1i). This is in line with previous reports showing fork collapse only at prolonged times of high HU treatment^38^.

**Extended Data Fig. 2:**
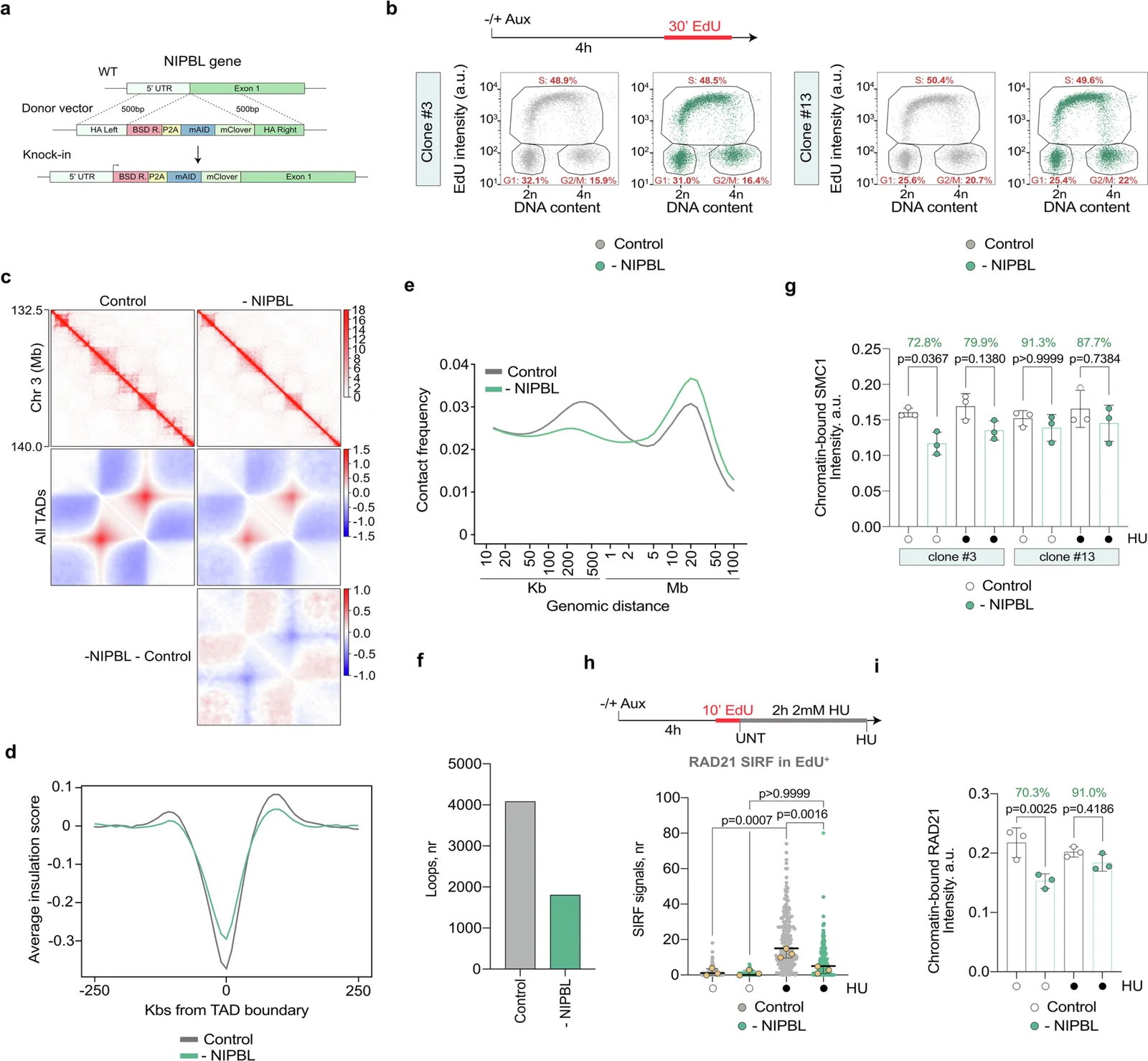
Characterization of DNA replication and genome organization after NIPBL depletion in mAID-mClover-NIPBL HCT116 cells. (**a**). Schematic of mAID-mClover cassette insertion into the start codon of the *NIPBL* gene. (**b**). EdU versus DAPI intensity flow cytometry profiles, after incorporation of EdU for 30 min. (**c**). Micro-C contact matrices from the indicated region. Below, aggregate analysis of chromatin contacts at re-scaled TADs. Differential enrichment of signals from aggregated analyses of -NIPBL minus Control. (**d**). Average insulation score around TAD boundaries. (**e**). Contact frequency distribution plotted as a function of genomic distance in the absence of NIPBL. (**f**). Quantification of the number of loops. (**g**). SMC1 intensity on chromatin after NIPBL depletion from the same cells as Fig. 1f. One-way ANOVA plus Bonferroni with exact p-values. n = 3 median values (error bars, +/− SD) of biological replicates (orange dots). Distribution values and median (black line) from one replicate are shown. (**h**). RAD21 SIRF upon 2 mM HU in NIPBL-degron cells. One-way ANOVA plus Bonferroni with exact p-values. n = 3 median values (error bars, +/− SD) of biological replicates (orange dots). Distribution values and median (black line) from one replicate are shown. (**i**). RAD21 intensity on chromatin after NIPBL depletion from the same cells as in (H). One-way ANOVA plus Bonferroni with exact p-values. n = 3 median values (error bars, +/− SD) of biological replicates (orange dots). Distribution values and median (black line) from one replicate are shown. Source data

Next, we asked which cohesin function drives its accumulation at stalled forks. To interrogate loop extrusion, we first confirmed by SIRF that NIPBL levels increase at stalled forks after 2 mM HU (Fig. 1e), in agreement with our iPOND data (Fig. 1b) and others^30^. Next, we generated HCT116 OsTIR1 (F74G)-expressing cell lines, in which NIPBL was endogenously and homozygously fused to a mini auxin-inducible degron (AID) and mini-Clover (mAID-mClover) protein tag, as a rapid and efficient inducer of protein degradation^6^ (Extended Data Fig. 2a). We confirmed a marked reduction of NIPBL cellular levels after addition of auxin (aux, 5-pH-IAA) in two independent clones (3 and 13) (Fig. 1f). Cell cycle progression and EdU incorporation in S-phase are not visibly affected by aux treatment (Extended Data Fig. 2b). Micro-C experiments show impaired TAD formation and insulation, decreased loop formation and increased compartmentalization (long-range interactions), as expected from a defect in loop extrusion^39^ (Extended Data Fig. 2c–f). Finally, after 2 mM HU treatment, NIPBL depletion abolished cohesin SMC1 and RAD21 accumulation at stalled forks (Fig. 1g,h and Extended Data Fig. 2h) with a marginal, non-significant increase in cohesin at stalled forks after HU treatment in NIPBL-depleted cells, probably reflecting incomplete NIPBL depletion in our degron system. This effect cannot be explained by impaired cohesin loading in the absence of NIPBL, because cohesin levels on chromatin are about 75–90% of those in the presence of NIPBL (Extended Data Fig. 2g,i), consistent with previous reports^40,41^.

**Extended Data Fig. 3:**
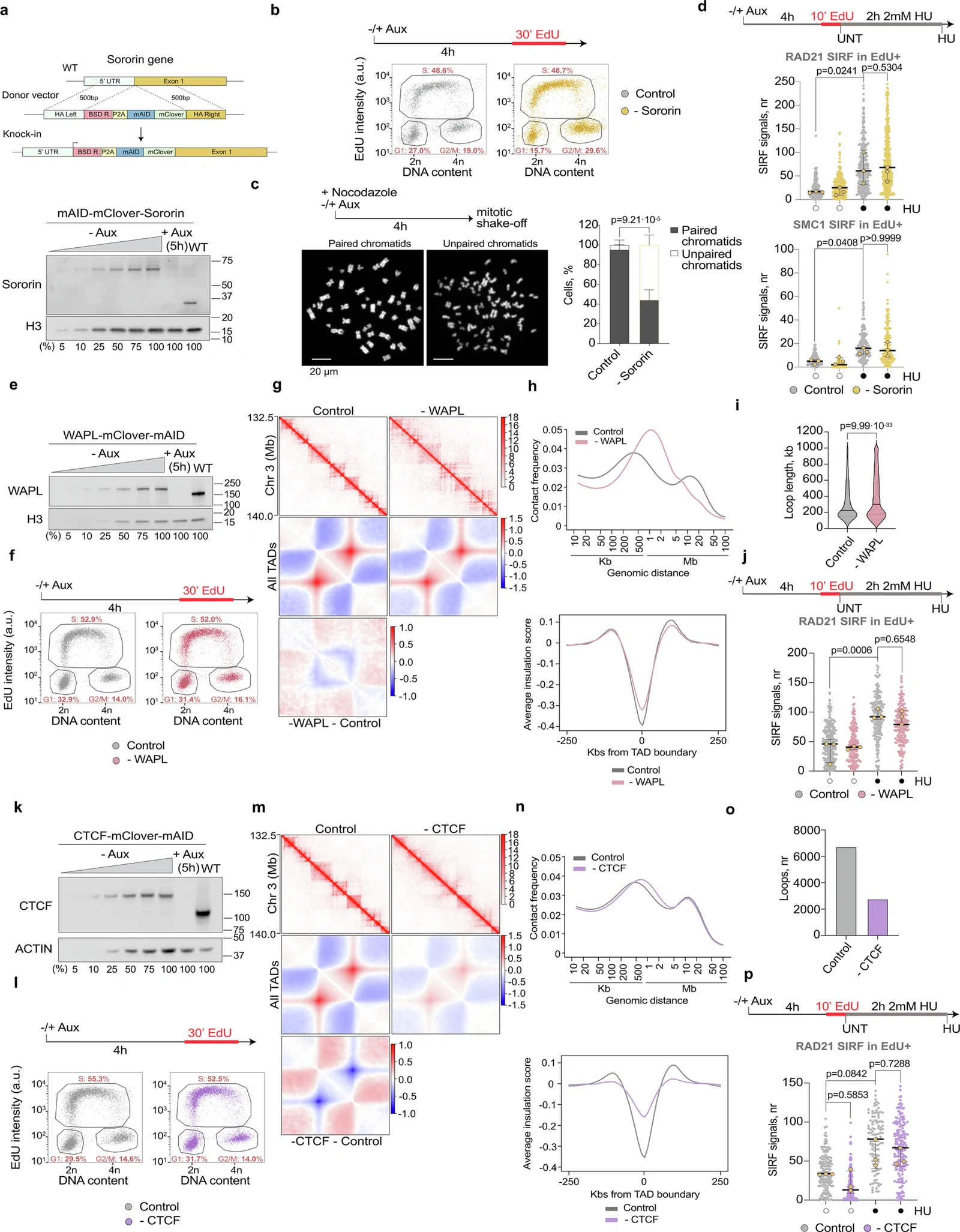
Effects of Sororin, WAPL or CTCF depletions. (**a**). mAID-mClover insertion strategy into the start codon of the *Sororin (CDCA5)* gene. Below, immunoblot of Sororin and H3 (loading control) in mAID-mClover-Sororin. Experiment repeated 3 times with similar results. Maker bands with molecular weight (in KDa) are shown. For gel source data, see Supplementary Fig. 1. (**b**). EdU/DAPI intensity flow cytometry after 30 min EdU. (**c**). Metaphase spreads after 4 h of Nocodazole and Aux. (**d**). RAD21 and SMC1 SIRFs upon 2 mM HU. One-way ANOVA plus Bonferroni with exact p-values. n = 3 median values (error bars, +/− SD) of biological replicates (orange dots). Distribution values and median (black line) from one replicate are shown. (**e**). Immunoblot of WAPL and H3 (loading control) in WAPL-mClover-mAID. Experiment repeated 3 times with similar results. Maker bands with molecular weight (in KDa) are shown. For gel source data, see Supplementary Fig. 1. (**f**). EdU/DAPI intensity flow cytometry after 30 min EdU. (**g**). Micro-C contact matrices from the indicated region. Below, aggregate analysis at re-scaled TADs. Differential enrichment of aggregated signals of -WAPL minus Control. (**h**). Average insulation score around TAD boundaries and contact frequency versus genomic distance. (**i**). Quantification of loop length. Two-tailed Mann-Whitney test with exact p-value. From left to right: n = 4807, 3873. Black line indicates the median; dashed lines indicate quartiles. (**j**). RAD21 SIRF upon 2 mM HU. One-way ANOVA plus Bonferroni with exact p-values. n = 3 median values (error bars, +/− SD) of biological replicates (orange dots). Distribution values and median (black line) from one replicate are shown. (**k**). Immunoblot of CTCF and ACTIN (loading control) in CTCF-mClover-mAID. Experiment repeated 3 times with similar results. Maker bands with molecular weight (in KDa) are shown. For gel source data, see Supplementary Fig. 1. (**l**). EdU/DAPI intensity flow cytometry after 30 min EdU. (**m**). Micro-C contact matrices from the indicated region. Below, aggregate analysis at re-scaled TADs. Differential enrichment of aggregated signals from -CTCF minus Control. (**n**). Average insulation score around TAD boundaries and contact frequency versus genomic distance. (**o**). Quantification of loop number. (**p**). RAD21 SIRF upon 2 mM HU. One-way ANOVA plus Bonferroni with exact p-values. n = 3 median values (error bars, +/− SD) of biological replicates (orange dots). Distribution values and median (black line) from one replicate are shown. Source data

As Sororin (CDCA5) was enriched at stalled forks in the iPOND–MS data (Fig. 1b), we tested whether the Sororin-mediated establishment of cohesion contributes to the observed accumulation of cohesin at forks. Using a degron strategy, validated by the loss of cohesion upon Sororin depletion (Extended Data Fig. 3a–c), we discarded this possibility for RAD21 and SMC1 subunits (Extended Data Fig. 3d). Likewise, we could not find any effect of WAPL depletion on cohesin recruitment at stalled forks, even if WAPL depletion proved to shift genomic contacts to a longer range, decreasing TAD insulation and increasing loop length (Extended Data Fig. 3e–j). These observations indicate that NIPBL is the only cohesin-associated factor required for its accumulation at stalled forks.

To address whether the lack of cohesin accumulation at stalled forks after NIPBL depletion is a consequence of deficient loop extrusion, disrupted genome organization or both, we analysed cohesin recruitment at HU-stalled replication forks after CTCF elimination using a previously generated degron cell line^6^. As expected, CTCF depletion impaired TAD formation and weakened insulation scores^42^. We observed a non-significant trend towards decreased cohesin enrichment near forks in the absence of RS but no effect on cohesin recruitment to stalled forks (Extended Data Fig. 3k–p). This result rules out an indirect effect of disrupted TAD organization in the absence of NIPBL and rather indicates that cohesin loading and/or loop extrusion are essential for cohesin accumulation at stalled forks.

## Cohesin reshapes fork contacts in response to RS

**Fig. 2:**
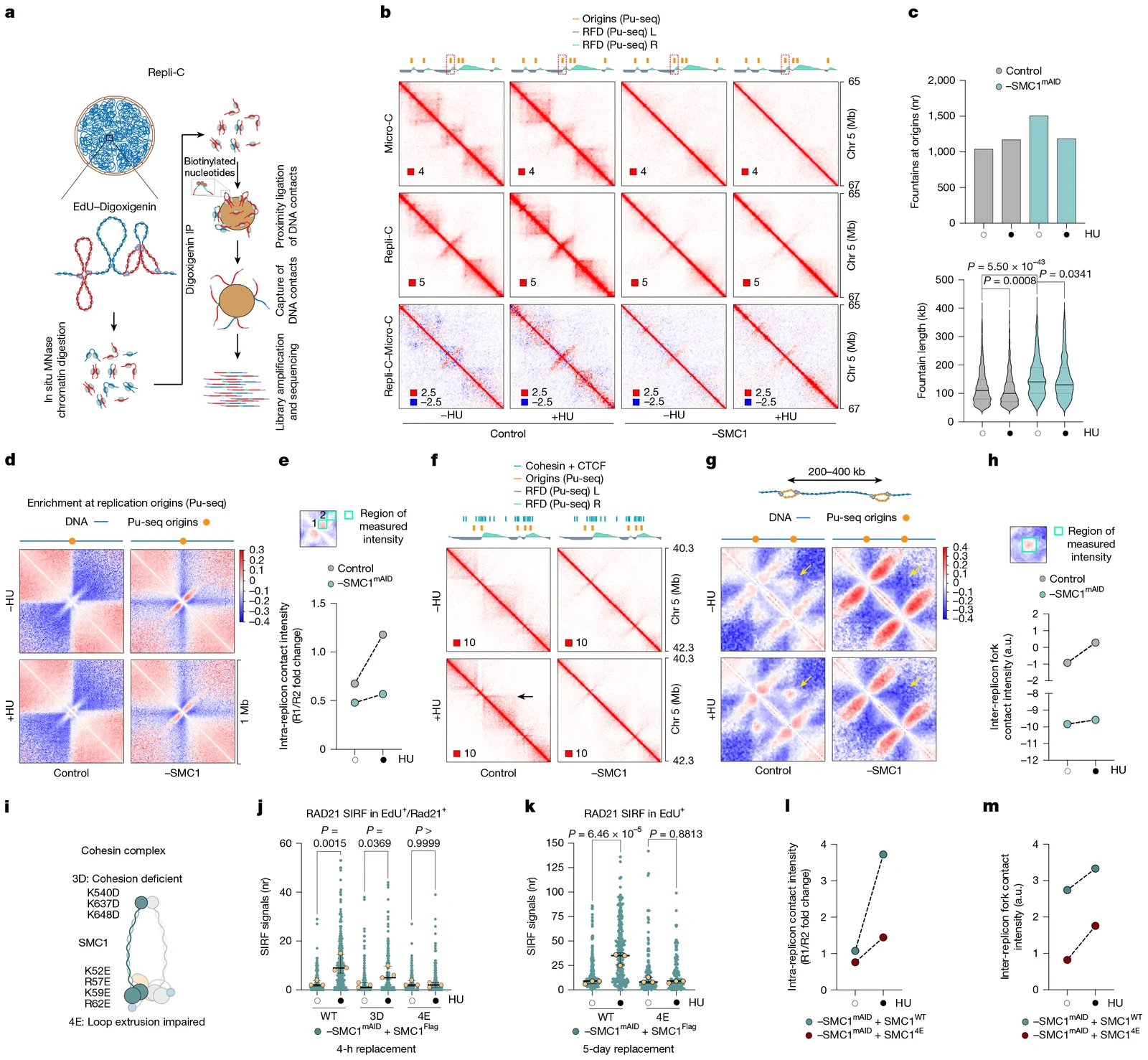
Cohesin reshapes genomic interactions at replication forks following RS. **a**, Experimental procedure of Repli-C (see text for details). IP, immunoprecipitation. **b**, Micro-C and Repli-C maps, and differential analysis in the indicated region. Origins and RFD (leftward (L) and rightward (R) forks; Pu-seq) are indicated. An origin displaying a fountain is highlighted by a dashed red box. **c**, Number and length of fountains at origin positions. Kruskal–Wallis plus Dunn’s test with exact *P*-values. From left to right, *n* = 1,014, 1,172, 1,506 and 1,186. Black line indicates the median; dashed lines indicate quartiles. **d**, Aggregated analysis of Repli-C contacts around centred origins (1 Mb). **e**, Contact intensity at replication origins from **d**. Fold changes of R1 (0–50 kb from diagonal) over R2 (50–100 kb from diagonal) are shown. **f**, Repli-C maps of the indicated region. Cohesin-CTCF, origins and RFD (Pu-seq) are indicated. A stripe structure is indicated by a black arrow in control + HU. **g**, Aggregate analysis of Repli-C contacts at scaled adjacent origins. Yellow arrows indicate the position of inter-replicon contacts. **h**, Inter-replicon contact intensities from **g** in the indicated region. **i**, SMC1 mutants used in this study. **j**, RAD21 SIRF in cells expressing SMC1^WT^, SMC1^3D^ and SMC1^4E^ after 4 h in aux. One-way ANOVA plus Bonferroni with exact *P*-values. *n* = 3 median values (error bars, ±s.d.) of biological replicates (orange dots). Distribution values and median (black line) from one replicate are shown. **k**, RAD21 SIRF in cells expressing SMC1^WT^ and SMC1^4E^ after 5 days in aux. One-way ANOVA plus Bonferroni with exact *P*-values. *n* = 3 median values (error bars, ±s.d.) of biological replicates (orange dots). Distribution values and median (black line) from one replicate are shown. **l**,**m**, Intra-replicon (**l**) and inter-replicon (**m**) contact intensities in SMC1^WT^ and SMC1^4E^ cells. Black circles indicate the addition of HU; white circles indicate that HU is absent. Source data

**Extended Data Fig. 4:**
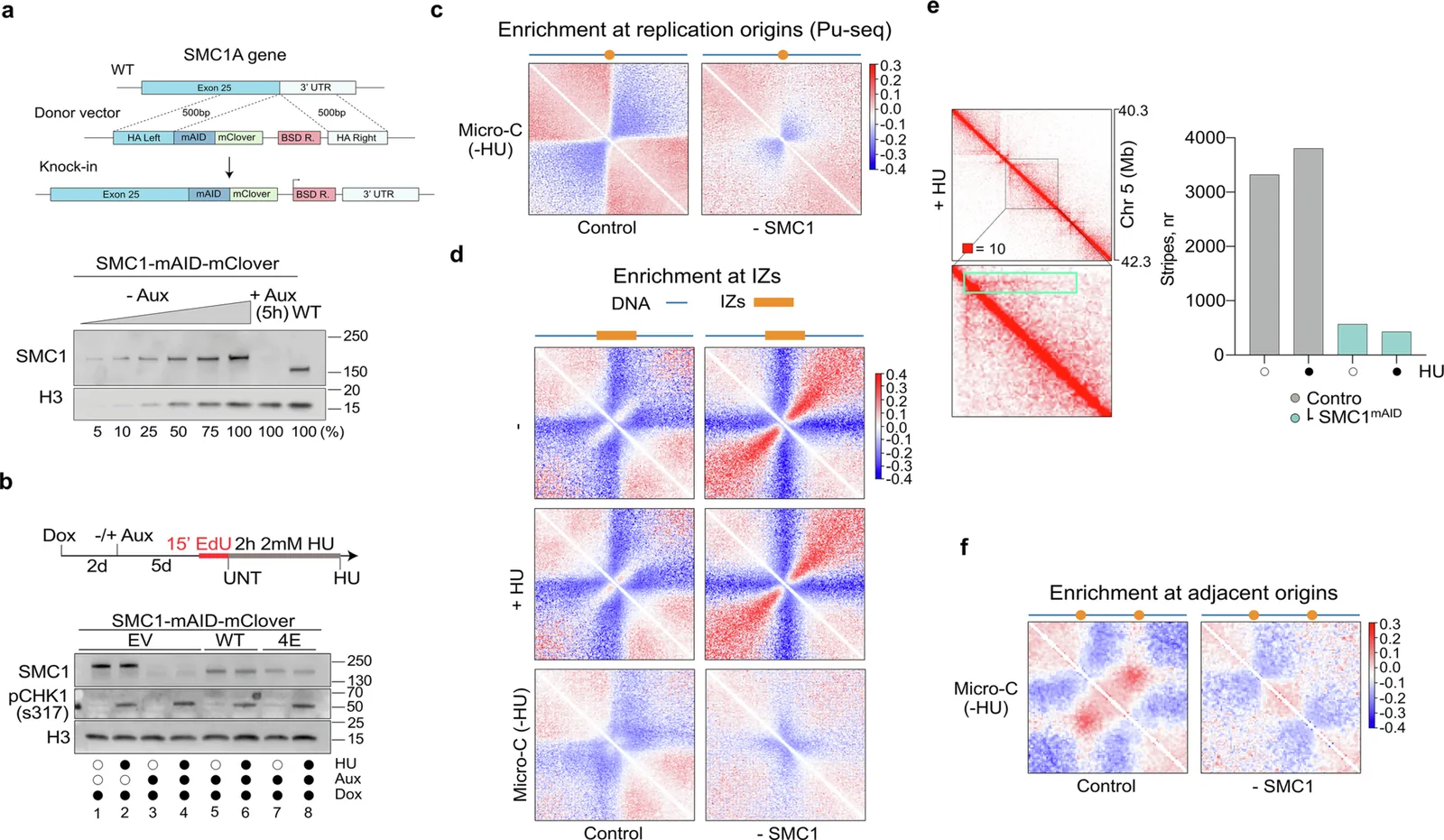
Analyses of Repli-C data upon RS and SMC1 depletion. (**a**). Schematic of mAID-mClover cassette insertion into the stop codon of the *SMC1A* gene. Immunoblot of SMC1 and H3 (loading control) in SMC1-mClover-mAID. Experiment repeated 3 times with similar results. Maker bands with molecular weight (in KDa) are shown. For gel source data, see Supplementary Fig. 1. (**b**). Experimental set up and immunoblot of SMC1, phospho-CHK1 (Ser317) and H3 (loading control) in SMC1-mClover-mAID cells. Experiment repeated 3 times with similar results. Maker bands with molecular weight (in KDa) are shown. For gel source data, see Supplementary Fig. 1. (**c**). Aggregated analysis of Micro-C contacts around replication origins (Pu-seq). (**d**). Aggregated analysis of Repli-C and Micro-C contacts around initiation zones. (**e**). Quantification of the number of stripes at the indicated conditions. (**f**). Aggregate analysis of Micro-C contacts at regions with adjacent origins (200-400 kb separation). Source data

If cohesin is recruited to stalled forks by NIPBL-dependent loop extrusion^2^, it may be required to reorganize chromatin contacts at replicating regions in response to RS. To test this hypothesis, we developed Repli-C, a Micro-C-based methodology to look at genomic contacts established at replicating regions by ligating DNA contacts after pulling down EdU-labelled nascent DNA (Fig. 2a). In short, cells were labelled with EdU for 15 min to focus our observations on the first kilobases of nascent DNA. EdU was linked to digoxigenin (Dig) through a click reaction. Cells were then exposed to controlled MNase digestion and chromatin contacts containing Dig–EdU were isolated. Finally, contacts were ligated, and libraries were prepared and sequenced. We obtained high-resolution maps of replication-associated genomic DNA contacts and identified ‘fountain-like’ structures at replication origins, previously described with Repli-Hi-C, which probably correspond to coupled sister forks^5^. These origins were identified as sites of replication fork divergence by analysing inflection points in replication fork directionality (RFD) profiles derived from polymerase usage sequencing (Pu-seq) data^43^. Fountain structures appear specifically in Repli-C maps, and not in parallel Micro-C analyses, confirming that they are replication-associated structures (Fig. 2b). To test the effect of cohesin depletion on these structures, we generated SMC1-mClover-mAID-expressing HCT116 cells, in which SMC1 can be depleted to less than 5% of the endogenous levels after a 5 h aux treatment (Extended Data Fig. 4a). We performed Repli-C in cells with or without cohesin and treated or not with 2 mM HU (Extended Data Fig. 4b, lanes 1–4). Visual inspection of Repli-C maps from all four conditions showed that fountains at origins get extended when cohesin is absent (Fig. 2b,c). This suggests that these replicative structures are largely cohesin-independent; their extended detection in the absence of cohesin may reflect the disruption of alternative contacts that replicating regions establish with other regions in a cohesin-dependent manner. This is in line with a recent report in yeast, showing cohesin-independent fountain structures at early-firing origins in Hi-C maps^44^. Quantification of the number and length of fountains by fountain detection analysis^5^ showed that SMC1 depletion generates many new fountains. Moreover, SMC1 depletion increased the fountain length, whereas HU consistently reduced it independently of cohesin (Fig. 2c). Hence, cohesin restricts fountain extension in unchallenged conditions, whereas HU promotes fountain shortening, suggesting that alternative cohesin-dependent and independent contacts may limit sister-fork proximity under stress.

Next, we plotted Repli-C contacts around all replication origins defined by Pu-seq^43^. We observed that HU shortened the fountain-like structure and concentrated new interactions close to the diagonal, that is, close to the origins of replication (intra-replicon contacts). This effect was not observed in the absence of cohesin, despite better detection of fountain-like structures (Fig. 2d, quantification in Fig. 2e). Importantly, no enrichment was found on Micro-C maps (Extended Data Fig. 4c), confirming the specificity of these contacts for replicating DNA. Similar observations were made when plotting Repli-C interactions at initiation zones identified by high-resolution Repli-seq^15^ (Extended Data Fig. 4d). Thus, after HU treatment, cohesin function is required to stabilize contacts at chromosomal regions proximal to origins.

In yeast Hi-C experiments, stalled replication forks were shown to act as barriers for cohesin-mediated loop extrusion^45^. Moreover, we noticed that HU markedly increased ‘stripes’ (Fig. 2f)—typically representing anchored loop extrusion in Hi-C and Micro-C maps—and confirmed that HU-induced stripes are dependent on cohesin (Extended Data Fig. 4e). We reasoned that increased loop extrusion on HU could be connecting replication forks from neighbouring replicons. To explore this possibility, we plotted Repli-C contacts at pairs of replication origins separated by a distance of 200–400 kb (in line with the average size of a replicon being 200 kb; ref. ^12^). In these Repli-C metaplots, clear contacts are detected between yet distant replication forks of neighbouring replicons—with each origin showing fountain-like structures (Fig. 2g)—while control Micro-C maps display spread contacts between the origins, similar to intra-TAD contacts, possibly owing to the frequent positioning of origins at TAD borders^11^ (Extended Data Fig. 4f). In both cases, inter-fork contacts disappear after SMC1 depletion. HU treatment markedly increased the frequency of these inter-replicon fork contacts, and this effect was entirely dependent on cohesin (Fig. 2f–h). Hence, cohesin establishes physical contacts between distant forks, which are stabilized under replication stress.

## Loop extrusion tethers adjacent forks

To further address the mechanism by which cohesin assists the response to replication stress, we set out to test recently described cohesin mutants^7–9,46^. In the SMC1^3D^ mutant, three lysine residues in the hinge domain are replaced by aspartic acid, and the resulting complex can sustain loop extrusion in vitro and shows a mild loop extrusion defect in vivo but is completely deficient in cohesion and cell proliferation^8^ (Fig. 2i). In the SMC1^4E^ mutant, four K/R residues critical for DNA binding are replaced by alanine, and the complex is unable to perform loop extrusion in vitro^7^, albeit partially retaining this function in vivo^9,46^ (Fig. 2i).

**Extended Data Fig. 5:**
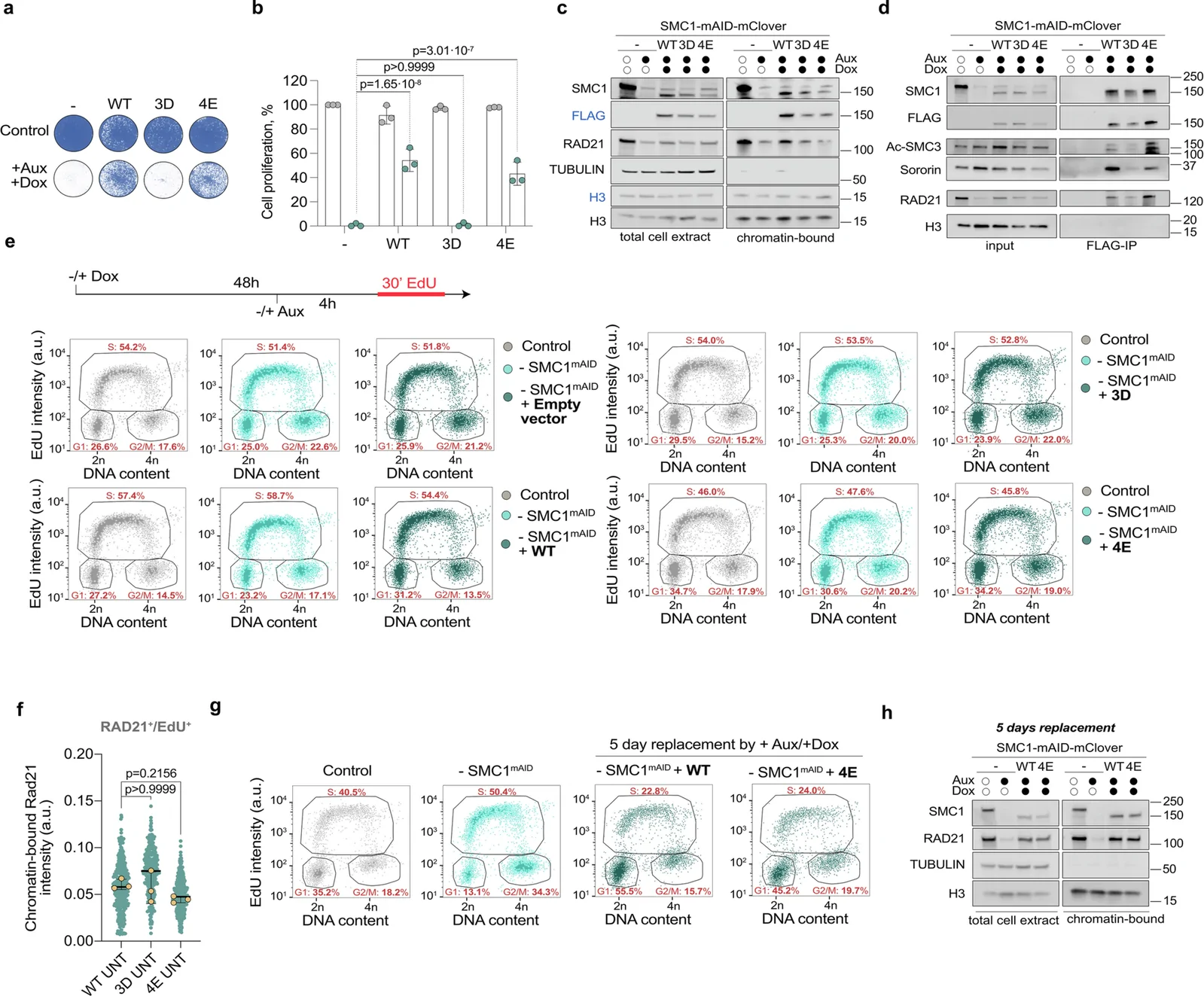
Characterization of cohesin mutants’ protein interactions and replication dynamics. (**a**). Representative images of cell proliferation staining. (**b**). Quantification of cell proliferation in the indicated SMC1-FLAG substituted conditions. Paired t-test plus Bonferroni with exact p-values. n = 3 median values (error bars, +/− SD) of biological replicates (circular dots). (**c**). Immunoblot of SMC1, FLAG, RAD21, TUBULIN (fractionation control) and H3 (loading control) in total cell extract and chromatin-bound fraction. Blue labels correspond to a separate WB run. Experiment repeated 3 times with similar results. Maker bands with molecular weight (in KDa) are shown. For gel source data, see Supplementary Fig. 1. (**d**). Immunoblot of SMC1, FLAG, Acetylated-SMC3, Sororin, RAD21 and H3 (immunoprecipitation control) after FLAG immunoprecipitation (IP). Experiment repeated 3 times with similar results. Maker bands with molecular weight (in KDa) are shown. For gel source data, see Supplementary Fig. 1. (**e** and **g**). EdU versus DAPI intensity flow cytometry profiles, after incorporation of EdU for 30 min. (**f**). Chromatin-bound RAD21 intensity in the experiment from Fig. 2j. One-way ANOVA plus Bonferroni with exact p-values. n = 3 median values (error bars, +/− SD) of biological replicates (orange dots). Distribution values and median (black line) from one replicate are shown. (**h**). Immunoblot of SMC1, RAD21, Tubulin (fractionation control) and H3 (loading control) in total cell extract and chromatin-bound fraction. Experiment repeated 3 times with similar results. Maker bands with molecular weight (in KDa) are shown. For gel source data, see Supplementary Fig. 1. Source data

Flag-tagged SMC1^WT^, SMC1^3D^ or SMC1^4E^ were ectopically expressed under the control of doxycycline (dox) in SMC1-mClover-mAID cells. We assessed the impact of these genetic perturbations on long-term cellular proliferation, by adding dox for 48 h before inducing endogenous SMC1 degradation for 7 days (Extended Data Fig. 5a). As expected, depletion of SMC1 had a major impact on cell proliferation that could be rescued by expression of ectopic SMC1^WT^ and SMC1^4E^, but not SMC1^3D^. This result confirms that the SMC1^3D^, but not the SMC1^4E^ mutation, impairs the essential function of cohesin in cohesion establishment (Extended Data Fig. 5a,b). Shortly after SMC1 replacement (48 h dox followed by 4 h aux), total cellular levels of all SMC1 variants were lower than endogenous SMC1 (Extended Data Fig. 5c). Nonetheless, they were recruited to chromatin as part of cohesin (Extended Data Fig. 5c,d), and complexes containing SMC1^4E^ but not SMC1^3D^ became acetylated and associated with Sororin, consistent with their ability and inability to perform cohesion, respectively (Extended Data Fig. 5d). Importantly, in these short-term experiments (48 h dox, 4 h aux), depletion of SMC1 and re-expression of the SMC1 variants did not have a major impact on the cell cycle and EdU incorporation (Extended Data Fig. 5e). However, in SIRF assays with cohesin RAD21 subunit, SMC1^WT^ and SMC1^3D^ cohesin complexes significantly accumulate at forks following HU treatment, whereas SMC1^4E^ cohesin does not (Fig. 2j). The intermediate phenotype observed for SMC1^3D^ is probably due to its incomplete loop extrusion activity in vivo because chromatin-bound levels in RAD21+/EdU+ cells are comparable to SMC1^WT^ and SMC1^4E^ (Extended Data Fig. 5f). These data confirm that cohesion is not required for cohesin recruitment at stalled forks.

**Extended Data Fig. 6:**
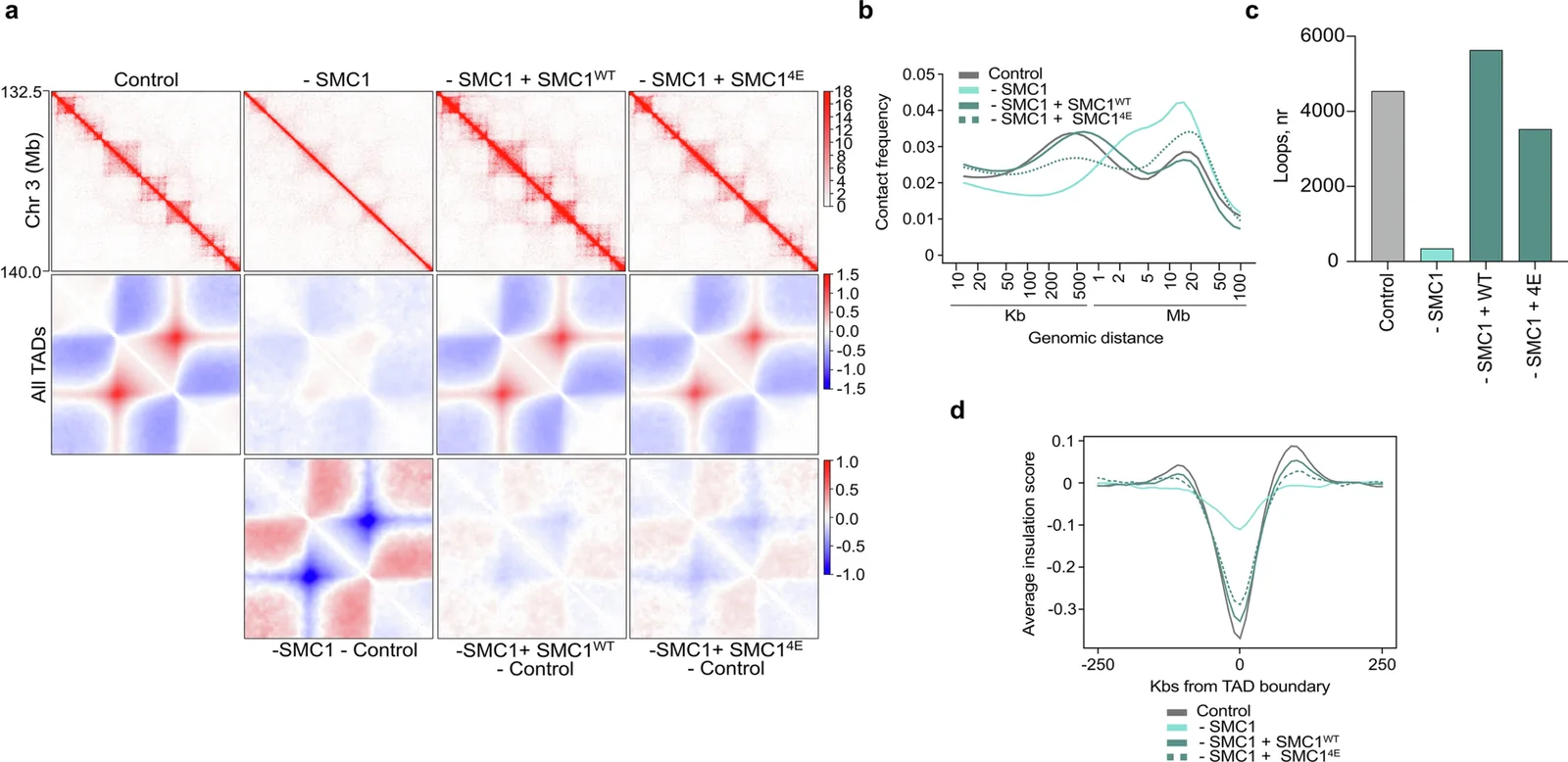
Analyses of genome organization of SMC1^4E^-substituted cells. (**a**). Micro-C contact matrices from the indicated region. Middle row, aggregate analysis of chromatin contacts at TADs. TADs are re-scaled to the same length. Bottom row, differential enrichment of signals from aggregated analyses of -SMC1, SMC1^WT^ or SMC1^4E^ minus Control. (**b**). Contact frequency distribution plotted as a function of genomic distance. (**c**). Quantification of the number of loops. (**d**). Average insulation score around TAD boundaries. Source data

**Extended Data Fig. 7:**
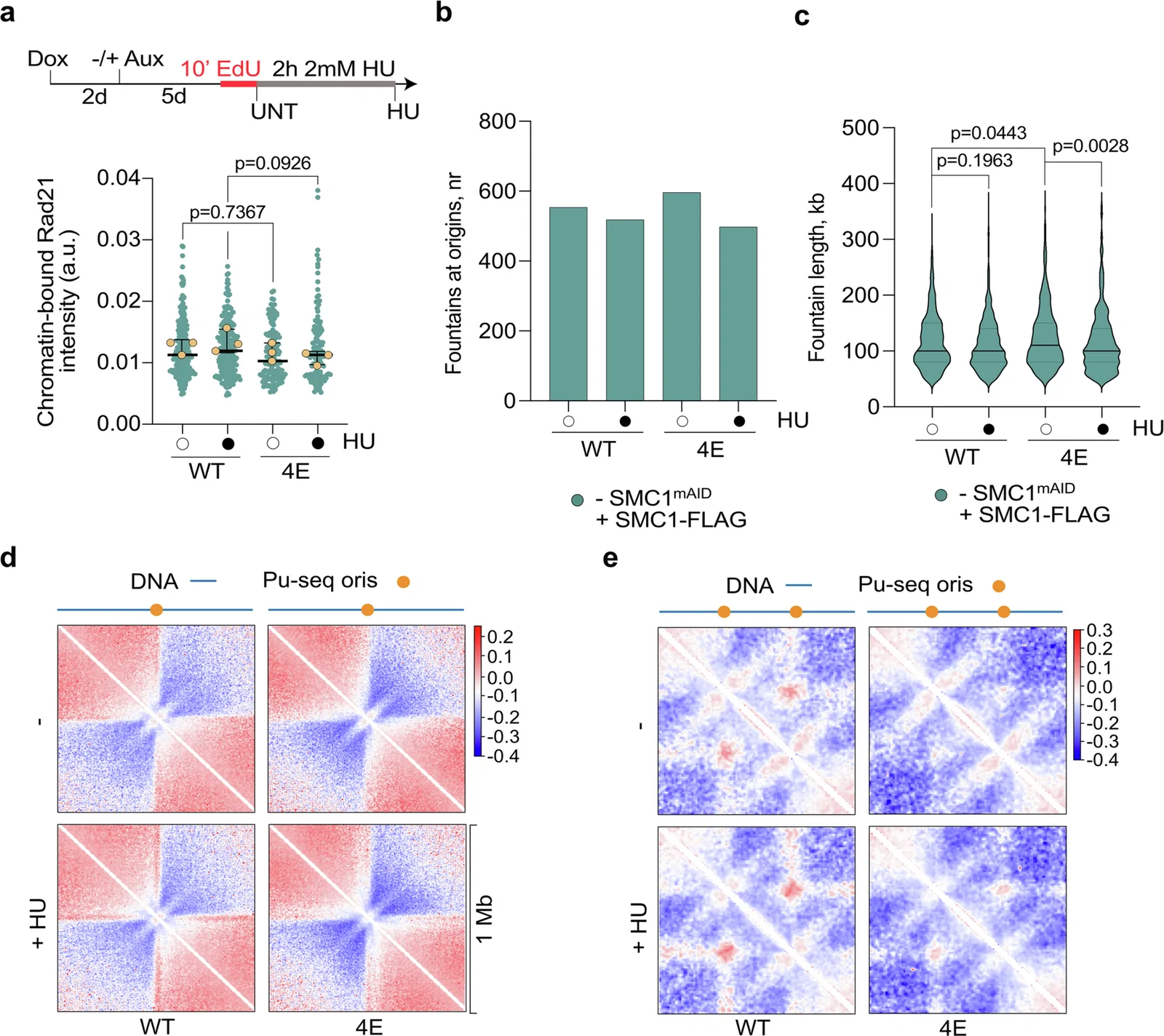
Chromatin binding of SMC1^4E^ and Repli-C analyses of SMC1^4E^-substituted cells upon RS. (**a**). Chromatin-bound RAD21 intensity in the experiment from Fig. 2k. One-way ANOVA plus Bonferroni with exact p-values. n = 3 median values (error bars, +/− SD) of biological replicates (orange dots). Distribution values and median (black line) from one replicate are shown. (**b** and **c**). Number and length of fountains identified at origin positions in the indicated conditions. Kruskal-Wallis plus Dunn’s test with exact p-values. From left to right: n = 554, 519, 597, 498. Black line indicates the median; dashed lines indicate quartiles. (**d**). Aggregated analysis of Repli-C contacts around centered origins (1 Mb). (**e**). Aggregate analysis of Repli-C contacts at regions with adjacent origins (200-400 kb separation). Source data

Next, we substituted endogenous SMC1 with SMC1^WT^ or SMC1^4E^ for 5 days in culture. This long-term substitution resulted in chromatin-bound cohesin levels similar to those in the parental cell line, as well as similar EdU incorporation (Extended Data Fig. 5g,h). In these conditions, we first interrogated genome organization by performing Micro-C. As expected, SMC1-depletion abolished TADs (Extended Data Fig. 6a), reduced contacts in the cohesin-mediated loop range (Extended Data Fig. 6b) and reduced the number of loops (Extended Data Fig. 6c), as well as TAD insulation (Extended Data Fig. 6d). Expression of SMC1^WT^ rescued all these phenotypes, whereas expression of SMC1^4E^ could only partially do so (Extended Data Fig. 6a–d), consistent with recent reports^46^. Having observed a defect in loop extrusion in SMC1^4E^ even after prolonged substitution, we tested its ability to be recruited at stalled forks by RAD21 SIRF. SMC1^4E^ could not support cohesin accumulation at HU-stalled replication forks (Fig. 2k), which is not merely a consequence of lower chromatin association of cohesin (Extended Data Fig. 7a), confirming that cohesin stabilization at stalled forks specifically requires loop extrusion.

Next, we used these controlled conditions of selective loop-extrusion impairment (SMC1^4E^) to assess by Repli-C the functional relevance of loop extrusion for the increased contacts induced by HU between sister forks and between forks belonging to adjacent replicons (Extended Data Fig. 4b, lanes 5–8). SMC1^4E^ exhibited a slight increase in fountain formation and length, and the latter was reduced after HU treatment (Extended Data Fig. 7b,c), consistent with sister-fork coupling being reduced upon RS, independently of loop extrusion. Similar to our previous experiments in the SMC1-degron cell line (Fig. 2d,e), we observed that HU treatment induces intra-replicon contacts close to the diagonal although the level was lower for SMC1^4E^ cells (Fig. 2l and Extended Data Fig. 7d), indicating that loop extrusion contributes to intra-replicon re-arrangement. However, a higher impact of loop extrusion was observed at inter-replicon fork contacts; SMC1^4E^ cells showed a striking decrease in these contacts in unchallenged conditions, that were only marginally increased after HU treatment (Fig. 2m and Extended Data Fig. 7e). Altogether, these results indicate that efficient loop extrusion stabilizes cohesin in proximity of stalled replication forks, inducing new three-dimensional (3D) contacts of replicating DNA that extend to neighbouring replicons.

## Cohesin limits PRIMPOL to reverse forks

**Fig. 3:**
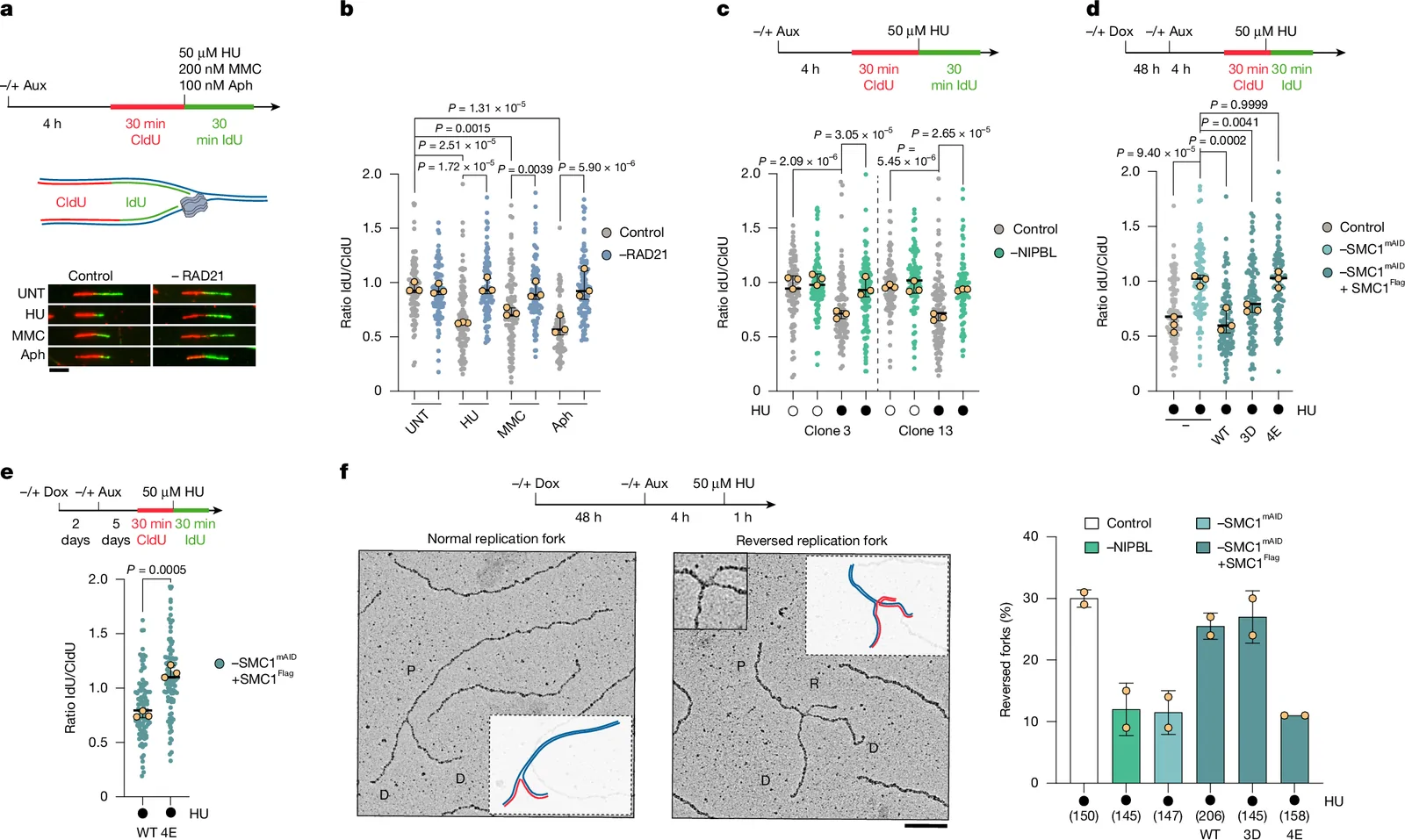
Loop-extruding cohesin promotes fork slowdown and reversal following RS. **a**, Scheme of the DNA fibre assay used to measure fork progression in RAD21-degron cells cultured in the presence of the indicated drugs during IdU labelling. Drawing represents incorporation of labelled nucleotides at ongoing forks. Representative images of individual labelled molecules are shown below. **b**, IdU/CldU DNA fibre values of **a**. One-way ANOVA plus Bonferroni with exact *P*-values. *n* = 3 median values (error bars, ±s.d.) of biological replicates (orange dots). Distribution values and median (black line) from one replicate are shown. **c**, IdU/CldU DNA fibre values (50 µM HU) in NIBPL-degron cells (3 and 13). One-way ANOVA plus Bonferroni with exact *P*-values. *n* = 3 median values (error bars, ±s.d.) of biological replicates (orange dots). Distribution values and median (black line) from one replicate are shown. **d**, IdU/CldU DNA fibre values (50 µM HU) in cells expressing SMC1^WT^, SMC1^3D^ and SMC1^4E^ after 4 h in aux. One-way ANOVA plus Bonferroni with exact *P*-values. *n* = 3 median values (error bars, ±s.d.) of biological replicates (orange dots). Distribution values and median (black line) from one replicate are shown. **e**, IdU/CldU DNA fibre values (50 µM HU) in cells expressing SMC1^WT^ and SMC1^4E^ after 5 days in aux. Paired *t*-test plus Bonferroni with exact *P*-values. *n* = 3 median values (error bars, ±s.d.) of biological replicates (orange dots). Distribution values and median (black line) from one replicate are shown. **f**, Representative images of normal and reversed forks (left). P, parental DNA; D, daughter strands; R, regressed arm. Explanatory drawings are included (blue, parental DNA; red, nascent DNA). Percentage of reversed forks measured after dox for 48 h, aux for the last 5 h and 50 µM HU during the last hour (right). The number of scored forks is indicated in brackets. Black circles indicate the addition of HU; white circles indicate that HU is absent. The experiment was performed two times. Median values (error bars, ±s.d.) of biological replicates (orange dots) are shown. Scale bar, 5 μm (**a**); 100 nm (**f**). Source data

**Extended Data Fig. 8:**
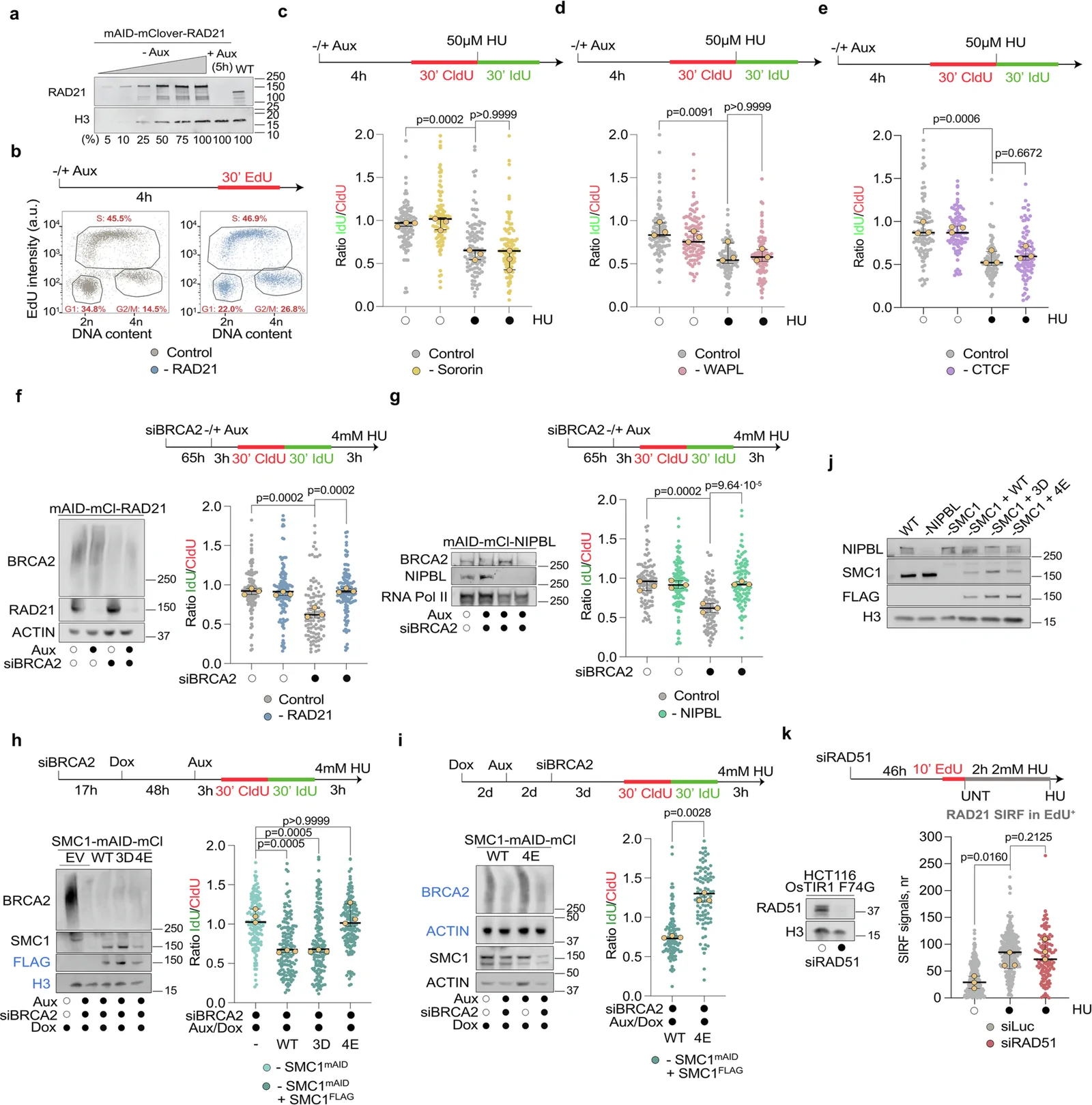
Regulation of fork progression and reversal by architectural factors. (**a**). Immunoblot of RAD21 and H3 (loading control) in mAID-mClover-RAD21. Experiment repeated 3 times with similar results. Maker bands with molecular weight (in KDa) are shown. For gel source data, see Supplementary Fig. 1. (**b**). EdU/DAPI intensity flow cytometry after 30 min EdU. (**c-e**). IdU/CldU DNA fiber values (upon 50 µM HU) in Sororin-, WAPL- and CTCF-degron cells. One-way ANOVA plus Bonferroni with exact p-values. n = 3 median values (error bars, +/− SD) of biological replicates (orange dots). Distribution values and median (black line) from one replicate are shown. (**f-i**). Immunoblot of BRCA2, RAD21 and ACTIN (loading control) (F), BRCA2, NIPBL and RNA Pol II (loading control) (G), BRCA2, SMC1, FLAG and H3 (loading control) (H), BRCA2, SMC1, and ACTIN (loading control) (I). Experiments repeated 3 times with similar results. Maker bands with molecular weight (in KDa) are shown. Blue labels correspond to a separate WB run. For gel source data, see Supplementary Fig. 1. At their right, IdU/CldU DNA fiber values (upon 4 mM HU, siBRCA2) in the indicated cell lines and treatments. For all, one-way ANOVA plus Bonferroni with exact p-values. n = 3 median values (error bars, +/− SD) of biological replicates (orange dots). Distribution values and median (black line) from one replicate are shown. **(j**). Immunoblot of NIPBL, SMC1, FLAG and H3 (loading control). Experiment repeated 3 times with similar results. Maker bands with molecular weight (in KDa) are shown. For gel source data, see Supplementary Fig. 1. (**k**). Immunoblot of RAD51 and H3 (loading control). Experiment repeated 3 times with similar results. Maker bands with molecular weight (in KDa) are shown. For gel source data, see Supplementary Fig. 1. RAD21 SIRF upon 2 mM HU. One-way ANOVA plus Bonferroni with exact p-values. n = 3 median values (error bars, +/− SD) of biological replicates (orange dots). Distribution values and median (black line) from one replicate are shown. Source data

Next, we tested the functional relevance of these cohesin-mediated intra- and inter-replicon fork contacts in the cellular response to RS. Cohesin accumulation due to defective unloading or regulation leads to replication fork slowdown^31,32^. To test the impact of acute cohesin inactivation on RS, we used a previously described mAID-mClover-RAD21 HCT116 cell line^6^, in which efficient RAD21 degradation has no major impact on the cell cycle and EdU incorporation profiles (Extended Data Fig. 8a,b). Single-molecule analysis of replication fork progression in the presence of 50 µM HU (which induces fork-reversal-dependent slowdown by ROS, independently of nucleotide depletion^36,47^), 200 nM MMC or 100 nM Aph, showed that these treatments reduced fork rate only in the presence of RAD21 (Fig. 3a,b). Depletion of NIPBL also led to unrestrained fork progression following HU addition (Fig. 3c), whereas Sororin, WAPL and CTCF depletions did not (Extended Data Fig. 8c–e). These results identify loop extrusion as the key cohesin function mediating the slowdown of stressed replication forks. Accordingly, ectopic expression of SMC1^WT^ and partly SMC1^3D^, but not SMC1^4E^, restores fork slowdown after HU treatment when endogenous SMC1 is depleted (Fig. 3d). Unrestrained fork progression in SMC1^4E^-expressing cells was also observed after long-term substitutions (Fig. 3e).

Fork slowdown following HU treatment is commonly associated with fork reversal. BRCA2 protects nascent DNA at stalled forks from nucleolytic activities by stabilizing RAD51 filaments, in a process that requires fork reversal. Hence, fork degradation in BRCA2-defective cells is often used as an indirect readout of proficient fork reversal^1^. After 3 h of 4 mM HU treatment, BRCA2 knockdown in mAID-mClover-RAD21 resulted in nascent DNA degradation (which impedes any DNA synthesis), as observed by the lower IdU/CldU ratio of single replication tracks (Extended Data Fig. 8f). However, co-depletion of RAD21 or NIPBL prevented this degradation (Extended Data Fig. 8f,g). Moreover, SMC1^WT^ and SMC1^3D^, but not SMC1^4E^, restore fork degradation in BRCA2/SMC1 co-depleted cells (Extended Data Fig. 8h). SMC1^4E^ could not restore fork degradation even in long-term substitutions (Extended Data Fig. 8i).

These results indicate that cohesin promotes fork reversal in an NIPBL-dependent manner, through its loop extrusion activity. Consistent with this possibility, fork reversal frequency assessed by electron microscopy^48^ was severely reduced in NIPBL-depleted cells and cells depleted of SMC1 or expressing SMC1^4E^ following HU treatment (Fig. 3f and Extended Data Fig. 8j). Furthermore, we observed that cohesin accumulation at stalled forks is not altered when fork reversal is genetically inactivated by RAD51 depletion, indicating that this accumulation precedes fork reversal (Extended Data Fig. 8k).

**Fig. 4:**
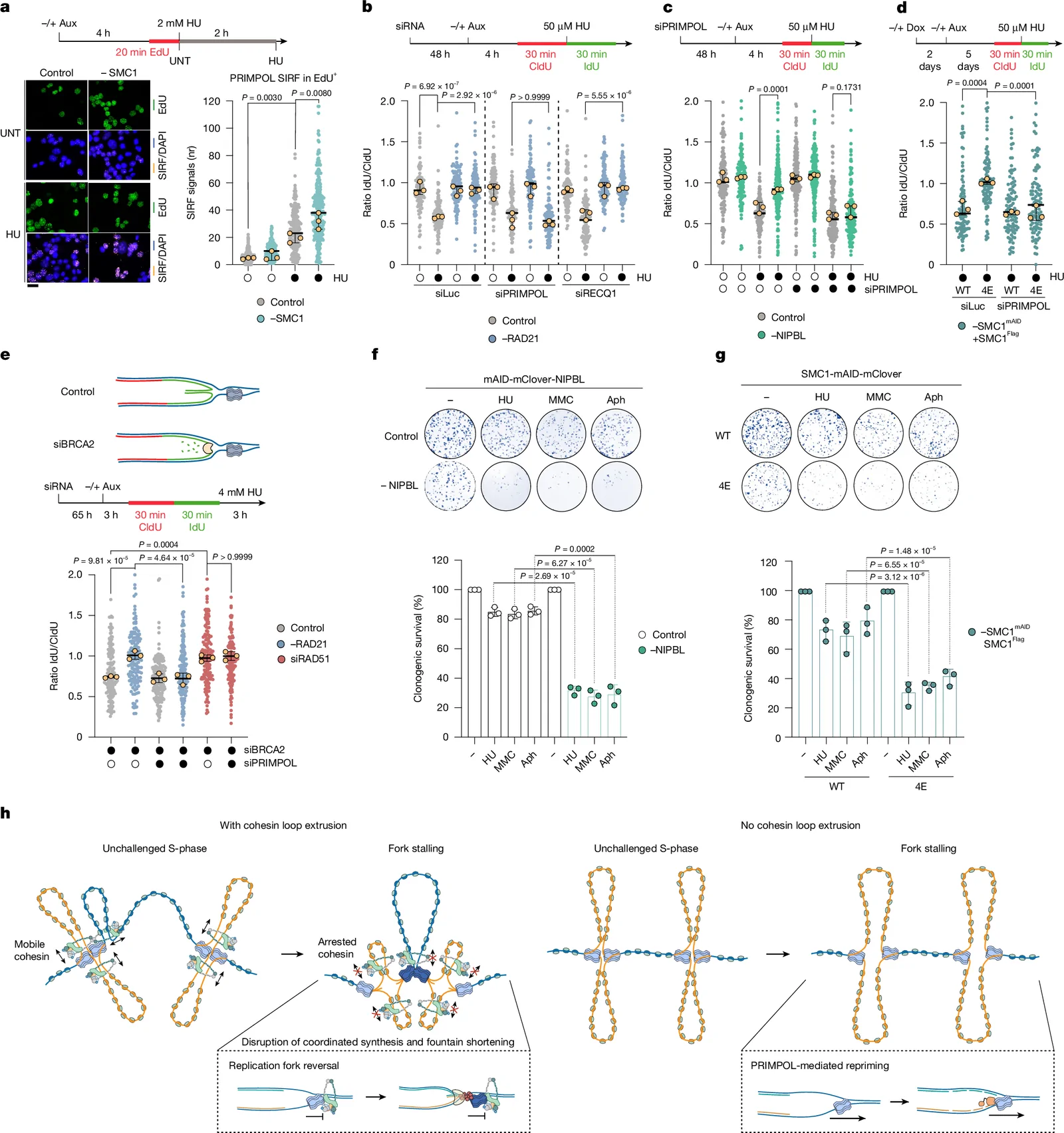
Loop-extrusion promotes fork slowdown and reversal by preventing PRIMPOL action. **a**, PRIMPOL SIRF after 2 mM HU in SMC1-degron cells. **b**–**e**, IdU/CldU DNA fibre values in the indicated conditions in RAD21-degron (**b**,**e**), NIPBL degron clone 13 (**c**) and SMC1 degron cells expressing SMC1^WT^ and SMC1^4E^ after 5 days in aux (**d**). Schemes of the experiments are shown above the graphs. One-way ANOVA plus Bonferroni with exact *P*-values. *n* = 3 median values (error bars, ±s.d.) of biological replicates (orange dots). Distribution values and median (black line) from one replicate are shown. In **e**, a drawing of fork degradation in the absence of BRCA2 is included. Black circles indicate the addition of HU; white circles indicate that HU is absent. **f**,**g**, Clonogenic assays in NIBPL-degron cells (**f**) and cells expressing SMC1^WT^ and SMC1^4E^ (**g**) in the presence of 150 µM HU, 50 nM Aph for 9 days or 90 nM MMC for 24 h followed by 8 days of recovery. Paired *t*-test plus Bonferroni with exact *P*-values. Median values (error bars, ±s.d.) of *n* = 3 biological replicates are represented (circular dots). **h**, During replication, mobile cohesin interacts with nascent DNA at coordinated replisomes. Fork stalling disrupts replisome coordination (dark blue CMGs) and arrests loop-extruding cohesin, creating inter-replicon contacts that promote fork reversal. The absence of cohesin favours PRIMPOL action, allowing unrestrained fork progression. Scale bar, 20 μm (**a**). Source data

**Extended Data Fig. 9:**
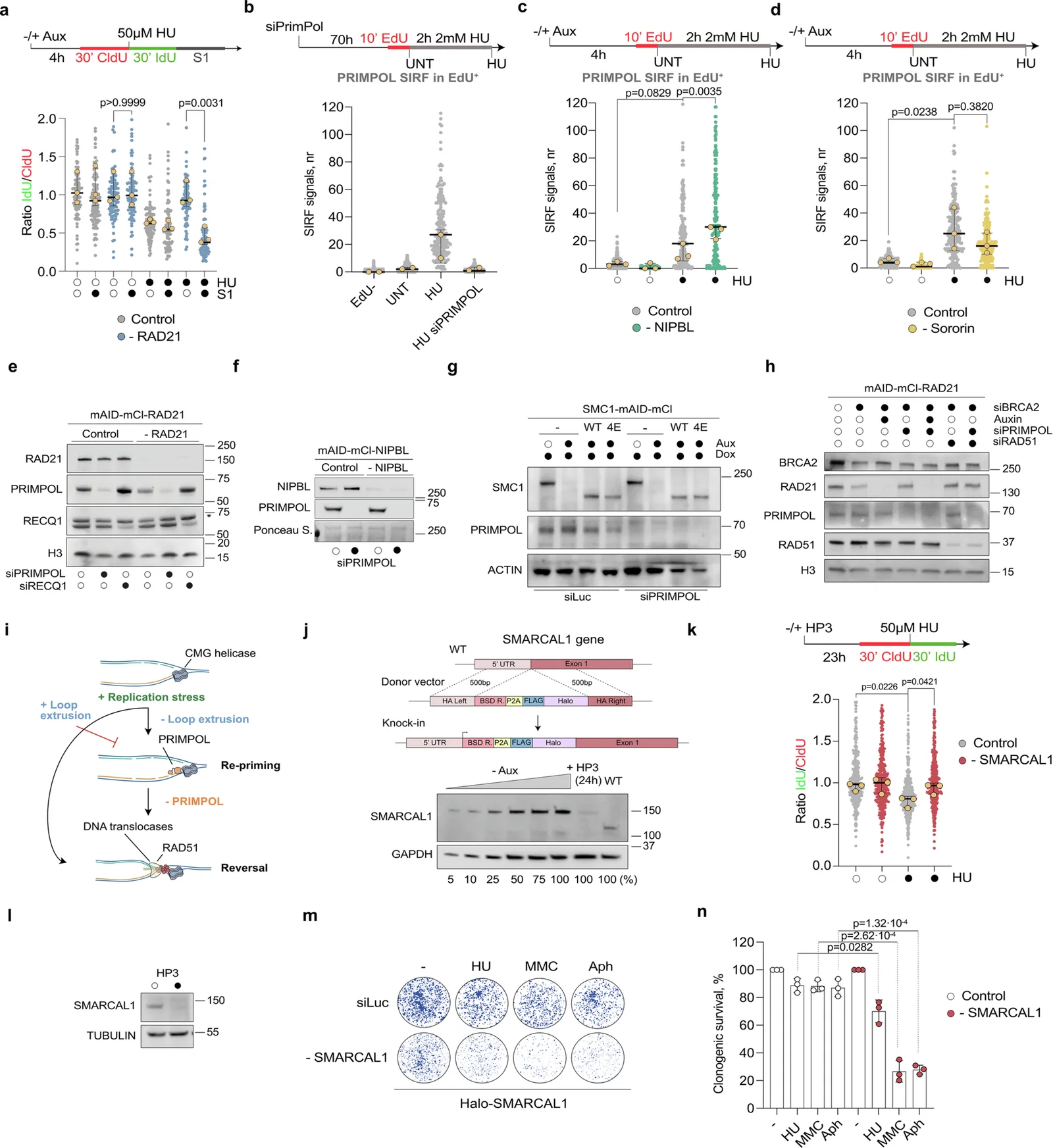
Role of PrimPol upon impairment of cohesin loop extrusion. **(a**). IdU/CldU DNA fiber values in RAD21-degron. One-way ANOVA plus szBonferroni with exact p-values. n = 3 median values (error bars, +/− SD) of biological replicates (orange dots). Distribution values and median (black line) from one replicate are shown. (**b**). PRIMPOL SIRF upon 2 mM HU in siPrimPol, (**c**) in NIPBL-degron and (**d**) Sororin-degron. One-way ANOVA plus Bonferroni with exact p-values. n = 3 median values (error bars, +/− SD) of biological replicates (orange dots). Distribution values and median (black line) from one replicate are shown. (**e**). Immunoblot of RAD21, PRIMPOL, RECQ1 and H3 (loading control), (**f**) NIPBL, PRIMPOL, and Ponceau staining (loading control), (**g**) SMC1, PRIMPOL, and ACTIN (loading control), (**h**). BRCA2, RAD21, PRIMPOL, RAD51 and H3 (loading control). Experiments repeated 3 times with similar results. Maker bands with molecular weight (in KDa) are shown. For gel source data, see Supplementary Fig. 1. (**i**). Role of loop-extruding cohesin in fork plasticity (see text for details). (**j**). FLAG-Halo cassette insertion into the start codon of the *SMARCAL1* gene. Below, immunoblot of SMARCAL1 and GAPDH (loading control) in FLAG-Halo-SMARCAL1. Experiment repeated 3 times with similar results. Maker bands with molecular weight (in KDa) are shown. For gel source data, see Supplementary Fig. 1. (**k**). IdU/CldU DNA fiber values (50 µM HU). One-way ANOVA plus Bonferroni with exact p-values. n = 3 median values (error bars, +/− SD) of biological replicates (orange dots). Distribution values and median (black line) from one replicate are shown. (**l**). Immunoblot of SMARCAL1 and TUBULIN (loading control) in FLAG-Halo-SMARCAL1. Experiment repeated 3 times with similar results. Maker bands with molecular weight (in KDa) are shown. For gel source data, see Supplementary Fig. 1. (**m**). Clonogenic assays in SMARCAL1-degron cells exposed to HU, Aph or MMC as in Fig. 4f and g. (**n**). Quantifications from (M). Paired t-test plus Bonferroni with exact p-values. n = 3 median values (error bars, +/− SD) of biological replicates (circular dots). Source data

To further investigate the consequences of the defective fork reversal and slowdown in the absence of cohesin, we interrogated the nature of the unrestrained DNA synthesis and the factors involved in RS tolerance. First, we combined DNA fibre assays with incubation with S1 nuclease, which attacks newly synthesized DNA only if it presents single-stranded DNA (ssDNA) gaps^49^. IdU tracks were shortened by S1 treatment in HU-treated RAD21-depleted cells, pointing towards discontinuous DNA synthesis (Extended Data Fig. 9a). The main contributor to discontinuous DNA synthesis following fork reversal defects is the PRIMPOL primase^47^. To test the possible role of PRIMPOL, we carried out PRIMPOL SIRF assays and showed that PRIMPOL increases at HU-stalled replication forks, an effect that is reversed by PRIMPOL downregulation (Extended Data Fig. 9b). Depletion of SMC1 or NIBPL, but not Sororin, further increased PRIMPOL levels at stalled replication forks (Fig. 4a and Extended Data Fig. 9c,d). To test the functional relevance of this increase, we downregulated PRIMPOL and observed that it prevented the unrestrained DNA synthesis of RAD21-depleted cells (Fig. 4b and Extended Data Fig. 9e), NIPBL-depleted cells (Fig. 4c and Extended Data Fig. 9f) and long-term substituted SMC1^4E^-expressing cells (Fig. 4d and Extended Data Fig. 9g), linking defective loop extrusion to deregulated PRIMPOL action. An alternative pathway possibly driving unrestrained fork progression requires the action of RECQ1 helicase^50,51^. However, depletion of RECQ1 (Extended Data Fig. 9e) had no effect on the unrestrained fork progression of RAD21-depleted cells treated with HU (Fig. 4b), indicating that reversed forks are unlikely to be formed in the first place in the absence of cohesin.

Next, we asked whether PRIMPOL depletion restores the ability of stalled forks to undergo fork reversal. To answer this question, we reverted to fork degradation assays in the absence of BRCA2. As shown before, RAD21 depletion prevented nascent DNA degradation in BRCA2-deficient cells (Extended Data Fig. 8f), but fork degradation was readily restored by PRIMPOL depletion (Fig. 4e and Extended Data Fig. 9h). Hence, defective loop extrusion impairs fork reversal due to PRIMPOL deregulation, although different from other genetic perturbations that drive PRIMPOL-dependent unrestrained fork progression (RAD51 depletion) in which fork reversal is not recovered after PRIMPOL depletion^52^ (Fig. 4e and Extended Data Fig. 9h).

Finally, we tested whether cohesin loop extrusion is important to maintain cellular fitness after long-term treatments with low doses of RS-inducing agents. For this purpose, we used NIPBL-depleted and SMC1^4E^-expressing cells, which can proliferate for long periods. Loss of NIPBL or SMC1^4E^ substitution had a main impact on the growth of single colonies after genotoxic treatments (Fig. 4f,g). This sensitivity is comparable to that observed after fork reversal inactivation, induced by acute depletion of endogenously tagged Halo-SMARCAL1 after 24-h treatment with Halo-PROTAC3 (HP3), as validated in DNA fibre assays (Extended Data Fig. 9j–n). These results further confirm that cohesin-mediated loop extrusion helps cells cope with RS during prolonged treatments. This sensitivity to genotoxic agents may partly reflect unleashed PRIMPOL action and toxic accumulation of ssDNA gaps. However, cohesin-mediated loop extrusion also plays a part in the repair of DSBs, which are likely to arise during the prolonged treatments used for these experiments^53^.

## Discussion

Here, we have observed that cohesin limits sister-fork coupling as forks progress away from the origins, restraining fountain extension within replicons. When S-phase is challenged by fork stalling agents, cohesin accumulates in proximity to stalled forks through loop extrusion. What stops cohesin at stressed replication forks awaits further investigation, but recent reports point at the MCM complex as a barrier for loop extrusion^20^. More specifically, the YDF domain at MCM3 was shown to stop cohesin in vitro when inserted into a yeast MCM3. This same domain is found at CTCF^21^. Whether an active CMG helicase can stop cohesin-mediated loop extrusion has not been assessed in vivo, but single-molecule imaging of encounters between cohesin complexes and replication forks using the *Xenopus* egg cell-free system indicates that cohesin is mainly pushed by the replisome^23^. Interactions between cohesin and the replisome during RS promote the tethering of neighbouring replicons and contribute to restraining the uncoupling of helicase and polymerase activities at the replisome, probably limiting the exposure of large ssDNA stretches that stimulate PRIMPOL repriming, at least in vitro^54^. Supercoiling generated by cohesin-mediated loop extrusion may further restrict helicase progression during uncoupling^48^. At the same time, preventing PRIMPOL action may also stabilize short ssDNA stretches arising during uncoupled DNA synthesis, which in turn facilitate the more complex biochemical reactions and DNA rearrangements required to drive fork reversal and ultimately maintain genome stability (Fig. 4h). We predict that this mechanism drives fork reversal and slowdown at stressed replication forks, and possibly contributes to complete stalling when cohesin stops at a CTCF binding site, in which stabilization is driven by G9-driven heterochromatinization of the replication fork-containing loop, as recently reported^55^. In summary, our results identify loop-extruding cohesin as a critical mediator of the cellular response to RS and to chemotherapeutic cancer treatments.

## Methods

### Cell culture

HCT116 cells and derivative cell lines (Supplementary Table 1) were grown in DMEM (Life Technologies, 41966-029) supplemented with 10% fetal bovine serum (FBS, GIBCO), 100 U ml^−1^ penicillin and 100 mg ml^−1^ streptomycin in an atmosphere containing 6% CO_2_ at 37 °C. HCT116 OsTIR (F74G), mAID-mClover-RAD21 HCT116 OsTIR (F74G)^6^, WAPL-mClover-mAID HCT116 OsTIR (F74G)^4^ and CTCF-mClover-mAID HCT116 OsTIR (F74G)^6^ were provided by M. Kanemaki. None of the cell lines were authenticated in house. All cell lines used in this study were subjected to regular mycoplasma testing in house, and all of them consistently reported negative results. To degrade mAID-tagged proteins, cells were treated with 2 µM 5-Ph-IAA (aux; BioAcademia). To degrade Halo-SMARCAL1, cells were treated with Halo-PROTAC3 at 1:1,000 for the indicated times.

### Cell line generation

Cell lines were generated following published protocols^56,57^. HCT116 OsTIR (F74G) cells were transfected with CRISPR-Cas9 and donor plasmids using FuGENE HD Transfection Reagent (Promega) in a 12-well plate following the instructions of the manufacturer. Two days after transfection, cells were plated in 10 cm^2^ dishes and selected with antibiotics. Selected clones were isolated and confirmed for protein expression of the modified alleles. Oligonucleotides to generate sgRNA or amplify homology arms to build donor plasmids are provided in Supplementary Table 1.

### RNA interference

For RNA interference, cells were transfected with the indicated siRNAs (Supplementary Table 1) for the indicated times and concentrations. Transfections were carried out using RNAiMax (Thermo Fisher) according to the instructions of the manufacturer.

### Lentiviral transduction

For cell lines expressing SMC1^WT^, SMC1^3D^ or SMC1^4E^, SMC1-3×Flag variant genes were cloned into LT3GEPIR (generated by GeneScript) and transfected into to HEK293T cells together with the lentiviral packaging vectors. After 48 h, lentiviral culture medium was harvested, filtered and added to SMC1-mClover-mAID HCT116 OsTIR (F74G) cells. After infection for 24 h, puromycin (2 μg ml^−1^, InvivoGen) resistant single cells were isolated. To induce the expression of the SMC1 variants, cells were treated with 1 µg ml^−1^ dox (Sigma-Aldrich).

### iPOND–MS

iPOND was performed as described^33,58^ with minor modifications. HCT116 cells were labelled with 10 µM EdU for 10 min and treated with the different drugs as indicated in Extended Data Fig. 1a. Cells were crosslinked with 1% formaldehyde for 20 min at room temperature (RT), quenched with 0.125 M glycine for 5 min, and washed three times with cold PBS. EdU was linked to biotin, after permeabilization with 0.25% Triton X-100/PBS for 30 min, by incubating in click reaction buffer (10 mM sodium-l-ascorbate, 20 µM biotin azide (Vanderbilt University) and 2 mM CuSO_4_) at RT for 1 h on a rotator. Cells were washed twice with PBS, resuspended in lysis buffer (50 mM Tris-HCl, pH 8.0 and 1% SDS) supplemented with protease inhibitors, and chromatin was solubilized by sonication in a Bioruptor Pico (Diagenode) at 4 °C 10 min (30 s on and 30 s off cycles). After centrifugation for 10 min at 16,000*g*, supernatants were diluted with 1:1 PBS (vol/vol) containing protease inhibitors and incubated overnight with myOne streptavidin C1 dynabeads (Thermo Fisher). Beads were washed once with lysis buffer, once with 1 M NaCl, twice with lysis buffer and once with PBS. Captured proteins were digested on beads using 500 ng of sequencing grade modified trypsin (Promega, V5111), including reduction and alkylation of cysteines with tris(2-carboxyethyl)phosphine hydrochloride (TCEP) and 2-chloroacetamide (ClAA) addition, respectively. Resulting peptides were cleaned using the Phoenix kit (Preomics) according to instructions in the kit. Liquid chromatography-tandem mass spectrometry analysis of peptide mixture was conducted on an Orbitrap Exploris 480 mass spectrometer (Thermo Fisher) directly coupled to an ACQUITY UPLC M-Class System (Waters) configured for 75-μm scale single-pump trapping. Peptides were separated on a nanoEase HSS C18 T3, 100 A, 75 μm × 250 mm analytical column (Waters, PN: 186008818) at a constant flow rate of 300 nl min^−1^ applying a peace-wise linear gradient from 5% to 33% solvent B in 45 min (solvent A: water, including 0.1% formic acid; solvent B: acetonitrile, including 0.1% formic acid). MS data acquisition was conducted in data-independent mode. DIA scans were acquired in the Orbitrap mass analyser at 15,000 Resolution (normalized AGC target: 3,000%, maxIT: 23 ms) covering the *m*/*z* range from 350 to 1,050 in 70 non-overlapping isolation windows. Precursors were quadrupole isolated at 10*m*/*z* and HCD fragmented at an NCE of 28 (calculated for the middle of the isolation window and charge 2).

Data analysis was performed using the PROSTAR package (v.1.34.6) in R (v.4.3.3). Protein intensity values were log_2_-transformed, and missing values for partially observed values were imputed using the SLSA (Structured Least Squares Algorithm). Data normalization was carried out first within groups and subsequently globally (excluding no biotin and thymidine chase controls) using the LOESS method. The normalized dataset was further processed in Perseus (v.1.6.10.50), in which remaining missing values were imputed from a normal distribution. For each comparison, proteins were retained only if at least two non-imputed valid values were present in at least one group. The resulting dataset was re-imported into PROSTAR, and differential abundance analysis was performed using the LIMMA test with a log_2_ fold-change threshold of 0.5. Benjamini–Hochberg correction was used to calculate *P*-values and FDR. Volcano plots were generated using GraphPad Prism (v.10).

### PLA and SIRF

Cells were asynchronously grown on poly-lysine-coated coverslips. Cells were labelled with 25 µM EdU (Thermo Fisher) for 10 min. After the indicated treatments, cells were washed with PBS and pre-extracted for 5 min using CSK buffer (10 mM HEPES, 50 mM NaCl, 0.3 M sucrose, 3 mM MgCl_2_, 1 mM EDTA or 1 mM EGTA and 0.5% Triton X-100) at 4 °C, followed by fixation in 4% formaldehyde at RT for 15 min. Following fixation, cells were washed three times with PBS. In SIRF experiments, EdU was linked to biotin by incubating in click reaction (0.1 M Tris, pH 8.5, 0.1 M sodium-l-ascorbate, 2 mM CuSO_4_ and 0.1 mM biotin azide) for 1 h at 37 °C in a humidified chamber. After three washes with PBS, the cells were incubated in blocking buffer (5% BSA in PBS) at 37 °C for 1 h and incubated overnight at 4 °C with the indicated antibodies (Supplementary Table 1). After washing twice for 5 min with TBST (0.05% Tween-20 in TBS), cells were incubated with PLA probes (Merck) for 1 h at 37 °C, ligated for 30 min at 37 °C and subjected to a polymerase reaction for 100 min at 37 °C according to the instructions of the manufacturer. After washing twice for 5 min with TBS, cells were incubated at 37 °C for 30 min with fluorescent-labelled secondary antibodies (Supplementary Table 1), against the individual targets of the PLA, in TBS containing DAPI (0.5 µg ml^−1^). Following two 5-min washes in TBS and letting coverslips air-dry in the dark, coverslips were mounted with Prolong Gold antifade reagent. Microscopy imaging was performed using a Leica DM6 B microscope (HCX PL APO 63× objective). SIRF/PLA and individual channel quantification was performed using Cell Profiler, and plotted and statistically assessed using GraphPad Prism 10. At least 100 cells were measured per condition.

### Biochemical fractionation, co-immunoprecipitation assays and Western blotting

Biochemical fractionation of cells was performed as previously described^33,58^. Co-immunoprecipitation experiments were performed by lysing cells in a buffer consisting of 20 mM Tris (pH 7.5), 150 mM NaCl, 5 mM MgCl_2_, 2 mM NaF, 10% glycerol, 0.2% NP40, 20 mM β-glycerophosphate, 0.5 mM DTT, protease inhibitor cocktail and Benzonase (Millipore), and rotated at 4 °C for 4 h. Lysates were centrifuged at 16,000*g* for 30 min at 4 °C. Supernatants were incubated with anti-Flag M2 affinity beads (Merck) overnight at 4 °C. Beads were washed three times with the lysis buffer. Biochemical fractions, co-immunoprecipitation beads and total cell extracts were prepared in Laemmli sample buffer (4% SDS, 20% glycerol and 120 mM Tris-HCl, pH 6.8) and loaded onto 4–20% Mini-PROTEAN TGX Precast Protein Gels (BioRad). For BRCA2 detection, extracts were run in 3–8% NuPAG Tris-Acetate Midi Protein Gels (Thermo Fisher). Proteins from all gels were separated by electrophoresis at 180 V followed by protein transfer to a nitrocellulose membrane in transfer buffer (25 mM Tris and 192 mM glycine) containing 20% methanol. Before addition of primary antibodies, membranes were blocked in 5% milk in 0.1% TBST (1× TBS supplemented with 0.1% Tween-20) for 1 h and incubated in 3% BSA with primary antibodies overnight at 4 °C (Supplementary Table 1). Secondary antibodies were added for 1 h at RT (in blocking solution; Supplementary Table 1). Membranes were washed three times with 0.1% TBST, 10 min each, after primary and secondary antibody incubations and detected with ECL detection reagent (GE Healthcare).

### Flow cytometry

Cell lines were labelled with 25 µM EdU for 30 min, harvested by standard trypsinization, washed with PBS once and fixed for 12 min with 4% formaldehyde in PBS. Cells were permeabilized with 0.25% Triton X in PBS, washed twice with PBS, and EdU was labelled by incubating in click reaction (0.1 M Tris pH 8.5, 0.1 M sodium-l-ascorbate, 2 mM CuSO_4_ and 0.1 mM Alexa Fluor-linked azide (Thermo Fisher)) for 1 h at RT. Cells were washed twice with PBS and DNA was stained with 1 μg ml^−1^ DAPI in PBS for 1 h. Samples were run on Attune NxT Flow Cytometer (Thermo Fisher) and analysed using FlowJo software v.10.0.8 (FlowJo). At least 10,000 cells were measured per condition. For gating strategy, see Supplementary Fig. 2.

### Metaphase spreads

Asynchronous mAID-mClover-Sororin cells were treated with 2 μM aux and/or nocodazole for a total of 4 h. Mitotic cells were collected by shake-off, incubated in hypotonic buffer (0.03 M sodium citrate), fixed in Carnoy’s solution, dropped on acid-washed slides and left overnight at RT. After staining with 1 μg ml^−1^ DAPI, slides were mounted with ProLong antifade and left to dry overnight before imaging in a Leica DM6000 microscope with LAS AF software.

### DNA fibre assays

All cell lines subjected to this analysis were grown asynchronously and labelled with 30 μM of the thymidine analogue 5-chloro-2′-deoxyuridine (CldU; Sigma-Aldrich) for 30 min, washed three times with warm PBS and exposed to 250 μM of 5-iodo-2′-deoxyuridine (IdU) for 30 min. For fork progression and S1 DNA fibre assessments, IdU was added alone or in combination with the indicated genotoxic treatments and concentrations. For fork degradation assays, IdU was added alone for 30 min, cells were washed three times with PBS and media containing 4 mM HU was added for 3 h. All cells were collected by trypsinization. For S1 assays, cells were incubated with S1 and prepared for spreading as described in ref. ^49^. All cells or nuclei were resuspended in cold PBS at 5 × 10^5^ cells ml^−1^ or 2 × 10^6^ nuclei ml^−1^. A total of 2 μl of this cell suspension was mixed with 7 μl of lysis buffer (200 mM Tris-HCl, pH 7.5, 50 mM EDTA and 0.5% SDS) on a glass slide. After incubating for 6 min at RT, the slides were tilted to a 45° angle to stretch the DNA fibres onto the slide. The resulting DNA spreads were air-dried and fixed in 3:1 methanol:acetic acid. The DNA fibres were denatured by incubating them in 2.5 M HCl for 1 h at RT, washed three times with PBS and blocked with 1% BSA in PBS-T (0.05% Tween-20 in PBS) for 1 h at RT. CldU and IdU tracks were stained for 2 h at RT using two different anti-BrdU antibodies recognizing CldU and IdU, respectively (Supplementary Table 1). After washing three times with PBS, slides were stained with secondary antibodies (Supplementary Table 1) for 1 h at RT in the dark. The slides were mounted in 30 μl Prolong Gold antifade reagent (Invitrogen). Microscopy was done using a Leica DM6 B microscope (HCX PL APO 63× objective). At least 100 fibres per sample were measured using ImageJ. Values were plotted and statistically assessed using GraphPad Prism 10.

### Electron microscopy

Cells were asynchronously grown, treated with 1 µg ml^−1^ dox for 48 h, 2 µM aux for 5 h and 50 µM HU where indicated. Cells were collected, resuspended in ice-cold PBS and crosslinked with 10 μg ml^−1^ 4,5′,8-trimethylpsoralen and pulses of 365 nm ultraviolet (UV) irradiation with monochromatic light (UV Stratalinker 1800; Agilent Technologies). DNA was extracted as previously described^48^. Briefly, cells were lysed (1.28 M sucrose, 40 mM Tris-HCl [pH 7.5], 20 mM MgCl_2_, and 4% Triton X-100; Qiagen) and digested (800 mM guanidine-HCl, 30 mM Tris-HCl pH 8.0, 30 mM EDTA pH 8.0, 5% Tween-20 and 0.5% Triton X-100) at 50 °C for 2 h in presence of 1 mg ml^−1^ proteinase K. The DNA was purified using chloroform:isoamylalcohol (24:1) and precipitated in one volume of isopropanol. DNA was washed with 70% EtOH and resuspended in 200 μl TE (Tris-EDTA) buffer. A total of 6 μg of was incubated with 120 U of PvuII HF (New England Biolabs) for 5 h at 37 °C. RNase A (Sigma-Aldrich, R5503) was added to a final concentration of 250 µg ml^−1^ for the last 2 h of this incubation. Digested DNA was purified using a Silica Bead Gel Extraction kit (Thermo Fisher Scientific) according to the instructions of the manufacturer. DNA was spread on carbon-coated 400-mesh nickel grids (G2400N, Plano, using benzyl-dimethyl-alkyl-ammonium chloride). DNA was then coated with platinum using a High Vacuum Evaporator (EM BAF060, Leica) as previously described^48^. The grids were imaged automatically at 28,000× using a Talos 120 transmission electron microscope (FEI; LaB6 filament, high tension ≤120 kV) with a bottom-mounted CMOS camera BM-Ceta (4,096 × 4,096 pixels) and the MAPS 3 software (Thermo Fisher Scientific). For analysis, the samples were annotated for replication intermediates using the MAPS offline viewer (v.3.28, Thermo Fisher Scientific), and the corresponding images were extracted. The replication intermediates were scored blind to the experimental condition using Fiji^59^. For each experimental condition, at least 65 replication fork molecules were analysed in two distinct biological replicates. The values were plotted and statistically assessed using GraphPad Prism 10.

### Proliferation and clonogenic assay

Cells were treated with 1 µg ml^−1^ of dox for 48 h (for WT and 4E-SMC1 substituted cells) and 2 µM aux (1 day before treatment in NIPBL-cells and 3 days before treatment in SMC1-substituted cells). In each well of a 6-well plate, 1,000 cells were seeded. The cells were then optionally treated with 90 nM MMC (Sigma-Aldrich) for 24 h, washed, and incubated in normal media, or treated with 150 µM HU (Sigma-Aldrich) or 50 nM Aph (Sigma-Aldrich) for 9 days to allow formation of colonies. Subsequently, colonies were stained with 0.5% (wt/vol) crystal violet in 20% ethanol. Plates were imaged using a plate reader, and the number of colonies was quantified using ImageJ. Values were normalized to the UNT control, and then plotted and statistically assessed using GraphPad Prism 10.

### Micro-C experiments

The Micro-C library was prepared using the Dovetail Micro-C Kit according to the protocol of the manufacturer. Briefly, cells were treated with 1 µg ml^−1^ dox for 48 h and 2 µM aux for 5 h where indicated. The chromatin was fixed with disuccinimidyl glutarate (DSG) and formaldehyde in the nucleus. The crosslinked chromatin was then digested in situ with micrococcal nuclease (MNase). After digestion, the cells were lysed with SDS to extract chromatin fragments, which were then bound to Chromatin Capture Beads. Next, the chromatin ends were repaired and ligated to a biotinylated bridge adapter, followed by proximity ligation of adapter-containing ends. After proximity ligation, the crosslinks were reversed, the associated proteins were degraded, and the DNA was purified and then converted into a sequencing library using Illumina-compatible adaptors. Biotin-containing fragments were isolated using streptavidin beads before PCR amplification.

### Repli-C experiments

Cells were treated with 1 µg ml^−1^ dox for 48 h, 2 µM aux for 5 h where indicated. A total of 10 µM EdU was added for 15 min, either before collection or before washing and treating cells with 2 mM HU for 2 h. Repli-C was performed using the Dovetail HiChIP MNase Kit with modifications. The cells were collected by trypsinization, the chromatin was fixed with DSG and formaldehyde and permeabilized with 0.25% Triton X in PBS. EdU was clicked to digoxigenin by incubating in a click reaction (0.1 M Tris, pH 8.5, 0.1 M sodium-l-ascorbate, 2 mM CuSO_4_ and 0.3 mM digoxigenin-azide (ATTBio)). Then, crosslinked chromatin was digested in situ with MNase and extracted after cell lysis using a combination of RIPA lysis and gentle sonication using Bioruptor Pico in RIPA buffer. The chromatin fragments were incubated with anti-digoxigenin antibody pre-coupled to A/G-coated beads, overnight rotating at 4 °C. Next, the chromatin ends were repaired and ligated to a biotinylated bridge adapter followed by proximity ligation of adapter-containing ends. After proximity ligation, the crosslinks were reversed, the associated proteins were degraded, and the DNA was purified and converted into a sequencing library using Illumina-compatible adaptors. Biotin-containing fragments were isolated using streptavidin beads before PCR amplification.

### Micro-C and Repli-C analyses

Micro-C and Repli-C data were processed using a standardized Hi-C analysis pipeline. Raw paired-end sequencing reads were quality-trimmed using Trim Galore (quality threshold = 20, minimum length = 30 bp) and aligned to the hg38 human reference genome using BWA-MEM^60^) with Hi-C-specific parameters (−5SP −T0). Aligned reads were processed with the pairtools suite: contacts were parsed with a minimum mapping quality of 40 (–min-mapq 40), allowing walks of up to five unique alignments (–walks-policy 5unique) with a maximum inter-alignment gap of 30 bp. Parsed pairs were sorted, and PCR duplicates were removed while retaining duplicate marking statistics. Valid contact pairs were converted to ‘.hic’ format using Juicer Tools (v.1.22.01) and to multi-resolution cool format (.mcool) using HiCExplorer with Knight–Ruiz (KR) matrix balancing normalization. For comparative analyses between conditions, biological replicates were merged using pairtools merge, and datasets were downsampled to equalize sequencing depth across conditions using pairtools sample. Downsampling fractions were calculated to match the condition with the lowest sequencing depth, ensuring unbiased comparisons (Supplementary Tables 2–6). Contact probability as a function of genomic distance was calculated as an intra-chromosomal contact frequency distribution, using logarithmically increasing genomic distance bins. The insulation score was computed using a custom Python script following the methodology described in ref. ^61^, extracting 10-kb resolution raw matrices and using a sliding window of 100 kb × 100 kb. Chromatin loops were detected using Chromosight^62^ on balanced contact matrices at 25 kb resolution, with a scanning distance range of 50 kb to 1 Mb. Moreover, architectural stripes (left and right patterns) were identified at 25 kb resolution.

Replication fountains, characterized by enriched long-range chromatin interactions radiating from replication origins, were identified using the FUN (Fountains Using Neighborhoods) algorithm^5^ on balanced contact matrices at 10 kb and 25 kb resolutions. Signal-over-noise (SoN) scores were calculated using a sliding window approach with an extension length of 500 kb, offset of 50 kb and padding widths of three bins (10 kb) or 1 bin (25 kb). Fountain structures were detected by extending outwards from SoN summits (extension pixels: 6–100 bins, step size 2 at 10 kb; 3–35 bins, step size 1 at 25 kb) and evaluated for statistical significance (*P*-value < 0.05, signal-to-background ratio >1.3).

Metaplots were created using coolpuppy^63^ on Knight–Ruiz balanced matrices. In the inter-replication fork metaplots, the –scale option was added to scale all the regions to the same size. Intra-replicon was quantified from metaplot diagonal decay profiles by calculating the log_2_ fold-change ratio between short-range (≤50 kb) and mid-range (50–100 kb) normalized contact frequencies. For each condition, diagonal decay was computed by extracting contact values at increasing distances from the diagonal using a sliding window approach (three bins each side of the centre point), with background normalization calculated from an extended window (10 bins). Values were log_2_-transformed and smoothed using a three-point moving average kernel. The intra-replicon contact index was defined as log_2_(mean contacts ≤50 kb/mean contacts 50–100 kb), in which higher values indicate increased short-range contacts. Values were plotted and statistically assessed using GraphPad Prism 10. Inter-replication fork interaction strength was quantified by extracting the sum of log_2_ contact frequencies from a 6 × 6 pixel region centred in the origin–origin interaction, representing the enrichment of contacts between paired replication origins.

### Identification of replication origins

Replication fork directionality (RFD) profiles were obtained from published Pu-seq data^43^, which quantifies the differential usage of leading and lagging strand DNA polymerases during replication. To identify replication origins, we analysed the RFD signal across the genome at 10-kb resolution. For each chromosome (excluding chrM and chrY), binned mean values were extracted and smoothed using a moving average filter (window size = 5) followed by Savitzky–Golay filtering (window = 15, polynomial order = 3) to preserve signal features while reducing noise. Replication origins and termination zones were identified by detecting inflection points in the smoothed RFD profile through second-derivative analysis. The second derivative captures the curvature of the signal, identifying the precise genomic positions at which replication fork dynamics fundamentally change. Replication origins were called as downward inflection points (second-derivative changes from positive to negative), corresponding to bidirectional fork movement away from the origin. For each putative origin, replication strength was calculated as the difference between the maximum RFD value in the downstream region (between the origin and the next termination zone) and the minimum RFD value in the upstream region (between the previous termination zone and the origin). Origins with replication strength below a threshold of 0.05 were considered weak and excluded from further analysis. When weak origins were removed, adjacent termination zones were merged by averaging their genomic positions. The remaining strong origins were reassigned to their flanking termination zones to ensure consistency between origin and termination zone annotations. Origins lacking flanking termination zones on either side were discarded^64–67^.

### Reporting summary

Further information on research design is available in the Nature Portfolio Reporting Summary linked to this article.

## Online content

Any methods, additional references, Nature Portfolio reporting summaries, source data, extended data, supplementary information, acknowledgements, peer review information; details of author contributions and competing interests; and statements of data and code availability are available at https://doi.org/10.1038/s41586-026-11034-0.

## Supplementary information

Supplementary Information This file contains Supplementary Tables 1–6 and Supplementary Figs. 1 and 2.

Reporting Summary

Peer Review File

## Source data

Source Data Fig. 1

Source Data Fig. 2

Source Data Fig. 3

Source Data Fig. 4

Source Data Extended Data Fig. 1

Source Data Extended Data Fig. 2

Source Data Extended Data Fig. 3

Source Data Extended Data Fig. 4

Source Data Extended Data Fig. 5

Source Data Extended Data Fig. 6

Source Data Extended Data Fig. 7

Source Data Extended Data Fig. 8

Source Data Extended Data Fig. 9

## Data Availability

Micro-C and Repli-C sequencing data presented in the present study were deposited in the NCBI Gene Expression Omnibus databases under the following accession IDs: GSE311634 and GSE311782. The proteomic data have been deposited in ProteomeXchange under the following accession ID: PXD071150. Source data are provided with this paper.
