# Supplementary material for: Cohesin reshapes replication fork contacts to aid fork slowing and reversal
Source: Nature. 2026 Sep 16;658(8137):1057–65. doi: 10.1038/s41586-026-11034-0 (PMC13686946; doi:10.1038/s41586-026-11034-0)
Supplement: Supplementary file 1 [file 41586_2026_11034_MOESM1_ESM.pdf]

nature portfolio
https://doi.org/10.1038/s41586-026-11034-0

Supplementary information

Cohesin reshapes replication fork contacts to aid fork slowing and reversal

In the format provided by the authors and unedited

Supplementary Table 1

Reagents table used in the study.

**Cell lines**		
**Reagent**	**Source**	**Identifier/Reference**
HCT116	ATCC	CCL-247
HCT116 OsTIR (F74G)	Kanemaki Lab	Reference #6
mAID-mClover-RAD21 HCT116 OsTIR (F74G)	Kanemaki Lab	Reference #6
WAPL-mClover-mAID HCT116 OsTIR (F74G)	Kanemaki Lab	Reference #4
CTCF-mClover-mAID HCT116 OsTIR (F74G)	Kanemaki Lab	Reference #6
mAID-mClover-NIBPL HCT116 OsTIR (F74G)	This study	This study
mAID-mClover-Sororin HCT116 OsTIR (F74G)	This study	This study
SMC1-mClover-mAID HCT116 OsTIR (F74G)	This study	This study
SMC1-mClover-mAID HCT116 OsTIR (F74G) + SMC1^{WT}-3xFLAG	This study	This study
SMC1-mClover-mAID HCT116 OsTIR (F74G) + SMC1^{3D}-3xFLAG	This study	This study
SMC1-mClover-mAID HCT116 OsTIR (F74G) + SMC1^{4E}-3xFLAG	This study	This study
FLAG-Halo-SMARCAL1 HCT116 OsTIR (F74G)	This study	This study
**Oligonucleotide**		
**Reagent**	**Sequence (5'-3')**	**Use**
NIBPL_sgRNA_Fw	CACCGCAACACCATTCCAGAAATTC	NIPBL CRISPR plasmid
NIBPL_sgRNA_Rev	AAACGAATTTCTGGAATGGTGTTGC	NIPBL CRISPR plasmid
NIBPL_HA_Fw	GCTAGAAAGGCAAGTTGAAGCTAGATT	NIPBL donor plasmid
NIBPL_HA_Rev	CAGGTAGGATGACACAGTAACTAAAATTGTAGG	NIPBL donor plasmid
NIBPL_invPCR_Fw	GGATCCAATGGGGATATGCCCCATGTC	NIPBL donor plasmid
NIBPL_invPCR_Rev	GTCGACTCTGAATTTCTGGAATGGTGTTGCC	NIPBL donor plasmid
Sororin_sgRNA_Fw	CACCGTCGCCTCCCAGACATAACTT	Sororin CRISPR plasmid
Sororin_sgRNA_Rev	AAACAAGTTATGTCTGGGAGGCGAC	Sororin CRISPR plasmid
Sororin_HA_Fw	GAGGCTCCCCGATACTACCG	Sororin donor plasmid
Sororin_HA_Rev	CACCCACAACACTCTCCTTAGCC	Sororin donor plasmid
Sororin_invPCR_Fw	GTCGACCTCCGTCTCGAGCTCCTCCA	Sororin donor plasmid
Sororin_invPCR_Rev	GGATCCTCTGGGAGGCGAACGCG	Sororin donor plasmid
SMC1_sgRNA_Fw	CACCGGCTTAGGGATCCAGACAGGGC	SMC1 CRISPR plasmid
SMC1_sgRNA_Rev	AAACGCCCTGTCTGGATCCCTAAGC	SMC1 CRISPR plasmid
SMC1_HA_Fw	CCTGTCTGGATCCCTAAGCTGT	SMC1 donor plasmid
SMC1_HA_Rev	AAGACTCCTATGAGGTCCTGAGTACC	SMC1 donor plasmid
SMC1_invPCR_Fw	AACCCCAATGAGCAGGGATCCGGTGCAGGCG	SMC1 donor plasmid
SMC1_invPCR_Rev	AGGGATCCAGACAGGGGATCCtAGTGAACCTCTTCGAGG	SMC1 donor plasmid
SMARCAL1_sgRNA_Fw	CACCGAATGTCCTTGCCTCTTACAG	SMARCAL1 CRISPR plasmid
SMARCAL1_sgRNA_Rev	AAACCTGTAAGAGGCAAGGACATTC	SMARCAL1 CRISPR plasmid
SMARCAL1_HA_Fw	TTTGAGAGTTTTGTTCTTGTTGCCC	SMARCAL1 donor plasmid
SMARCAL1_HA_Rev	TTTCACAGAGAAATGCTTATGCAGGAATG	SMARCAL1 donor plasmid
SMARCAL1_invPCR_Fw	AGTCTTCCTCTTACAGAGGAGCAGAGGAAAAAG	SMARCAL1 donor plasmid
SMARCAL1_invPCR_Rev	AGAGGCTTATGAGTTGGGTTAGC	SMARCAL1 donor plasmid
**RNAi**		
**Reagent**	**Sequence (5'-3')**	**Concentration and time**
siLuc	CGUACGCGGAAUACUUCGATT	100 nM; 48-72h
siPRIMPOL	GAGGAAACCGUUGUCCUCAGUGUAU	40 nM; 48-72h
siRECQ1	UUACCAGUUACCAGCAUUATT	40 nM; 48h
siBRCA2	UUGACUGAGGCUUGCUCAGUUTT	100 nM; 72h
siRAD51	GAGCUUGACAAACUACUUCTT	40 nM; 48-72h
**Antibody**		
**Target**	**Source, Identifier**	**Technique (concentration)**
SMC1 #1 (Rb)	Losada Lab, Reference #40	WB (1:1000), IF/PLA (1:200)
SMC1 #2 (Rb)	Thermo Fisher, PA5-29122	WB (1:1000)
NIPBL (Rb)	Losada Lab, Reference #40	WB (1:500), IF/PLA (1:200)
RAD21 (Rb)	Abcam, ab217678	WB (1:1000), IF/PLA (1:200)
Sororin (Rb)	Losada Lab, Reference #64	WB (1:1000)
WAPL (Rb)	Cell Signaling, 77428	WB (1:1000)
CTCF (Rb)	Cell Signaling, 3418	WB (1:1000)
Ac-SMC3 (Ms)	Merck, MABE1925	WB (1:1000)
RNA Pol II RPB1 (Ms)	BioLegend, 920204	WB (1:1000)
H3 (Rb)	Abcam, ab1791	WB (1:10000)
ACTIN (Ms)	Sigma-Aldrich, A2228	WB (1:5000)
FLAG (Ms)	Merck, F3165	WB (1:2000)
PRIMPOL (Rat)	Méndez Lab Reference #65	WB (No dilution, membranes submerged in the antibody-containing supernatant).
MCM2 (Ms)	Thermo Fisher, MA5-15923	IF/PLA (1:500)
RECQ1 (Rb)	Abcam, ab172607	WB (1:1000)
RAD51 (Rb)	BioAcademia, 70-002	WB (1:1000)
BRCA2 (Ms)	Sigma-Aldrich, OP95	WB (1:1000)
Tubulin (Ms)	Sigma-Aldrich, T6199	WB (1:10000)
SMARCAL1 (Rb)	Abcam, ab154226	WB (1:1000)
GAPDH (Ms)	Sigma-Aldrich, MAB374	WB (1:5000)
phospho-H2AX (Ser 139) (Rb)	Cell Signaling, 9718	IF (1:100)
phospho-ATM (Ser 1981) (Ms)	Thermo Fisher, 200-301-400	IF (1:1000)
phospho-Chk1 (Ser 317) (Rb)	Cell Signaling, 12302	WB (1:1000)
Biotin (Ms)	Jackson ImmunoResearch, 200-002-211	IF/PLA (1:200)
Digoxigenin (Ms)	MBL, M227-3	Repli-C (coupled to beads)
BrdU (CldU; Rat)	Abcam, ab6326	IF (1:150)
BrdU (IdU; Ms)	Becton Dickinson, 347580	IF (1:100)
Alexa 488 (Ms IgG1)	Invitrogen, A-21121	IF (1:300)
Alexa 555 (Rat)	Invitrogen, A-21434	IF (1:300)
Alexa 488 (Ms)	Invitrogen, A-11001	IF (1:500)
Alexa 488 (Rb)	Invitrogen, A-11034	IF (1:500)
Alexa 555 (Ms)	Invitrogen, A-21422	IF (1:500)
Alexa 555 (Rb)	Invitrogen, A-21428	IF (1:500)
Alexa 647 (Ms)	Invitrogen, A-31571	IF (1:500)
F(ab')2 Alexa 647 (Rb)	Invitrogen, A-21246	IF (1:500)
ECL IgG, HRP-linked (Ms)	Sigma-Aldrich, NA934	WB (1:5000)
ECL IgG, HRP-linked (Rb)	Sigma-Aldrich, GENA931	WB (1:5000)
IgG, Cross-Adsorbed, HRP (Rat)	Thermo Fisher, A10549	WB (1:5000)
**Publicly available data**		
**Description**	**Source**	**Identifier; Password for reviewers**
iPOND-MS	This study	PXD071150
Micro-C and Repli-C	This study	GSE311634<br>GSE311782
Pu-seq origins and termination zones	Reference #43	GSE189668
HR Repli-seq initiation zones	Reference #15	https://data.4dnucleome.org/experiment-sets/4DNESNGZM5FG/
TADs	Reference #66	GSE104334
Cohesin and CTCF ChIP-seq	Reference #67	GSE167887

Supplementary Table 2

Sequencing stats Micro-C: The amount and percentage of all read types per sample and replicate are provided. Stats corresponding to UNT data sets used in Fig. 2 and Extended Data Fig. 2, 4 and 6.

NIPBLkd_1_dedup.stats		
Total Read Pairs	408912081	100.00 %
Unmapped Read Pairs	19584022	4.79%
Mapped Read Pairs	330162661	80.74%
PCR Dup Read Pairs	140049149	34.25%
No-Dup Read Pairs	190113512	46.49%
No-Dup Cis Read Pairs	86146304	45.31%
No-Dup Trans Read Pairs	103967208	54.69%
No-Dup Valid Read Pairs (cis >= 1kb + trans)	136418599	71.76%
No-Dup Cis Read Pairs < 1kb	53694913	28.24%
No-Dup Cis Read Pairs >= 1kb	32451391	17.07%
No-Dup Cis Read Pairs >= 10kb	25256143	13.28%

SMC1kd_1_dedup.stats		
Total Read Pairs	221423546	100.00 %
Unmapped Read Pairs	10563875	4.77%
Mapped Read Pairs	179562476	81.09%
PCR Dup Read Pairs	46354907	20.93%
No-Dup Read Pairs	133207569	60.16%
No-Dup Cis Read Pairs	57210177	42.95%
No-Dup Trans Read Pairs	75997392	57.05%
No-Dup Valid Read Pairs (cis >= 1kb + trans)	98812416	74.18%
No-Dup Cis Read Pairs < 1kb	34395153	25.82%
No-Dup Cis Read Pairs >= 1kb	22815024	17.13%
No-Dup Cis Read Pairs >= 10kb	19081479	14.32%

NIPBLkd_2_dedup.stats		
Total Read Pairs	325143233	100.00 %
Unmapped Read Pairs	19531336	6.01%
Mapped Read Pairs	273926489	84.25%
PCR Dup Read Pairs	83455289	25.67%
No-Dup Read Pairs	190471200	58.58%
No-Dup Cis Read Pairs	152461705	80.04%
No-Dup Trans Read Pairs	38009495	19.96%
No-Dup Valid Read Pairs (cis >= 1kb + trans)	121630957	63.86%
No-Dup Cis Read Pairs < 1kb	68840243	36.14%
No-Dup Cis Read Pairs >= 1kb	83621462	43.90%
No-Dup Cis Read Pairs >= 10kb	63654008	33.42%

SMC1kd_2_dedup.stats		
Total Read Pairs	249251935	100.00 %
Unmapped Read Pairs	12342784	4.95%
Mapped Read Pairs	209059500	83.87%
PCR Dup Read Pairs	75939478	30.47%
No-Dup Read Pairs	133120022	53.41%
No-Dup Cis Read Pairs	102569277	77.05%
No-Dup Trans Read Pairs	30550745	22.95%
No-Dup Valid Read Pairs (cis >= 1kb + trans)	99377800	74.65%
No-Dup Cis Read Pairs < 1kb	33742222	25.35%
No-Dup Cis Read Pairs >= 1kb	68827055	51.70%
No-Dup Cis Read Pairs >= 10kb	54394314	40.86%

SMC1kd_SMC14Edox_1_dedup.stats		
Total Read Pairs	89125996	100.00 %
Unmapped Read Pairs	5015966	5.63%
Mapped Read Pairs	73487886	82.45%
PCR Dup Read Pairs	29817118	33.46%
No-Dup Read Pairs	43670768	49.00%
No-Dup Cis Read Pairs	28993720	66.39%
No-Dup Trans Read Pairs	14677048	33.61%
No-Dup Valid Read Pairs (cis >= 1kb + trans)	25824397	59.13%
No-Dup Cis Read Pairs < 1kb	17846371	40.87%
No-Dup Cis Read Pairs >= 1kb	11147349	25.53%
No-Dup Cis Read Pairs >= 10kb	9169104	21.00%

SMC1kd_SMC1dox_1_dedup.stats		
Total Read Pairs	163220498	100.00 %
Unmapped Read Pairs	8933869	5.47%
Mapped Read Pairs	134404550	82.35%
PCR Dup Read Pairs	67335230	41.25%
No-Dup Read Pairs	67069320	41.09%
No-Dup Cis Read Pairs	46003552	68.59%
No-Dup Trans Read Pairs	21065768	31.41%
No-Dup Valid Read Pairs (cis >= 1kb + trans)	43772757	65.26%
No-Dup Cis Read Pairs < 1kb	23296563	34.74%
No-Dup Cis Read Pairs >= 1kb	22706989	33.86%
No-Dup Cis Read Pairs >= 10kb	18500365	27.58%

SMC1kd_SMC14Edox_2_dedup.stats		
Total Read Pairs	425196443	100.00 %
Unmapped Read Pairs	22968261	5.40%
Mapped Read Pairs	361314694	84.98%
PCR Dup Read Pairs	229679580	54.02%
No-Dup Read Pairs	131635114	30.96%
No-Dup Cis Read Pairs	104281072	79.22%
No-Dup Trans Read Pairs	27354042	20.78%
No-Dup Valid Read Pairs (cis >= 1kb + trans)	80953854	61.50%
No-Dup Cis Read Pairs < 1kb	50681260	38.50%
No-Dup Cis Read Pairs >= 1kb	53599812	40.72%
No-Dup Cis Read Pairs >= 10kb	41813733	31.76%

SMC1kd_SMC1dox_2_dedup.stats		
Total Read Pairs	383001237	100.00 %
Unmapped Read Pairs	19803333	5.17%
Mapped Read Pairs	321368727	83.91%
PCR Dup Read Pairs	133502849	34.86%
No-Dup Read Pairs	187865878	49.05%
No-Dup Cis Read Pairs	148242869	78.91%
No-Dup Trans Read Pairs	39623009	21.09%
No-Dup Valid Read Pairs (cis >= 1kb + trans)	141928022	75.55%
No-Dup Cis Read Pairs < 1kb	45937856	24.45%
No-Dup Cis Read Pairs >= 1kb	102305013	54.46%
No-Dup Cis Read Pairs >= 10kb	79149690	42.13%

WT_1_dedup.stats		
Total Read Pairs	271980368	100.00 %
Unmapped Read Pairs	11794046	4.34%
Mapped Read Pairs	220123780	80.93%
PCR Dup Read Pairs	68388919	25.14%
No-Dup Read Pairs	151734861	55.79%
No-Dup Cis Read Pairs	70757219	46.63%
No-Dup Trans Read Pairs	80977642	53.37%
No-Dup Valid Read Pairs (cis >= 1kb + trans)	116170567	76.56%
No-Dup Cis Read Pairs < 1kb	35564294	23.44%
No-Dup Cis Read Pairs >= 1kb	35192925	23.19%
No-Dup Cis Read Pairs >= 10kb	29172704	19.23%

WT-NIPBL_1_dedup.stats		
Total Read Pairs	142954863	100.00 %
Unmapped Read Pairs	7114814	4.98%
Mapped Read Pairs	116491615	81.49%
PCR Dup Read Pairs	28443965	19.90%
No-Dup Read Pairs	88047650	61.59%
No-Dup Cis Read Pairs	40503166	46.00%
No-Dup Trans Read Pairs	47544484	54.00%
No-Dup Valid Read Pairs (cis >= 1kb + trans)	61159669	69.46%
No-Dup Cis Read Pairs < 1kb	26887981	30.54%
No-Dup Cis Read Pairs >= 1kb	13615185	15.46%
No-Dup Cis Read Pairs >= 10kb	10932433	12.42%

WT_2_dedup.stats		
Total Read Pairs	243539966	100.00 %
Unmapped Read Pairs	12279324	5.04%
Mapped Read Pairs	204369201	83.92%
PCR Dup Read Pairs	107336309	44.07%
No-Dup Read Pairs	97032892	39.84%
No-Dup Cis Read Pairs	78302599	80.70%
No-Dup Trans Read Pairs	18730293	19.30%
No-Dup Valid Read Pairs (cis >= 1kb + trans)	68033720	70.11%
No-Dup Cis Read Pairs < 1kb	28999172	29.89%
No-Dup Cis Read Pairs >= 1kb	49303427	50.81%
No-Dup Cis Read Pairs >= 10kb	39449413	40.66%

WT-NIPBL_2_dedup.stats		
Total Read Pairs	359764978	100.00 %
Unmapped Read Pairs	23733999	6.60%
Mapped Read Pairs	305057605	84.79%
PCR Dup Read Pairs	125705125	34.94%
No-Dup Read Pairs	179352480	49.85%
No-Dup Cis Read Pairs	148872723	83.01%
No-Dup Trans Read Pairs	30479757	16.99%
No-Dup Valid Read Pairs (cis >= 1kb + trans)	96342413	53.72%
No-Dup Cis Read Pairs < 1kb	83010067	46.28%
No-Dup Cis Read Pairs >= 1kb	65862656	36.72%
No-Dup Cis Read Pairs >= 10kb	50370485	28.08%

Supplementary Table 3

Sequencing stats Micro-C: The amount and percentage of all read types per sample and replicate are provided.
Stats corresponding to SMC1-degron HU, and WAPL and CTCF data sets used in Fig. 2 and Extended Data Fig. 2, 3 and 4.

SMC1_HU_1_stats.txt		
Total Read Pairs	171912536	100.00%
Unmapped Read Pairs	9352278	5.44%
Mapped Read Pairs	141583087	82.36%
PCR Dup Read Pairs	63778653	37.10%
No-Dup Read Pairs	77804434	45.26%
No-Dup Cis Read Pairs	50166879	64.48%
No-Dup Trans Read Pairs	27637555	35.52%
No-Dup Valid Read Pairs (cis >= 1kb + trans)	49884158	64.11%
No-Dup Cis Read Pairs < 1kb	27920276	35.89%
No-Dup Cis Read Pairs >= 1kb	22246603	28.59%
No-Dup Cis Read Pairs >= 10kb	18257076	23.47%

SMC1_HU_Auxin_1_stats.txt		
Total Read Pairs	160735856	100.00%
Unmapped Read Pairs	6502944	4.05%
Mapped Read Pairs	128467096	79.92%
PCR Dup Read Pairs	36599963	22.77%
No-Dup Read Pairs	91867133	57.15%
No-Dup Cis Read Pairs	38607679	42.03%
No-Dup Trans Read Pairs	53259454	57.97%
No-Dup Valid Read Pairs (cis >= 1kb + trans)	72305422	78.71%
No-Dup Cis Read Pairs < 1kb	19561711	21.29%
No-Dup Cis Read Pairs >= 1kb	19045968	20.73%
No-Dup Cis Read Pairs >= 10kb	15699754	17.09%

SMC1_HU_2_stats.txt		
Total Read Pairs	160321193	100.00%
Unmapped Read Pairs	12483565	7.79%
Mapped Read Pairs	137863683	85.99%
PCR Dup Read Pairs	37316926	23.28%
No-Dup Read Pairs	100546757	62.72%
No-Dup Cis Read Pairs	94274831	93.76%
No-Dup Trans Read Pairs	6271926	6.24%
No-Dup Valid Read Pairs (cis >= 1kb + trans)	32662550	32.48%
No-Dup Cis Read Pairs < 1kb	67884207	67.52%
No-Dup Cis Read Pairs >= 1kb	26390624	26.25%
No-Dup Cis Read Pairs >= 10kb	19710568	19.60%

SMC1_HU_Auxin_2_stats.txt		
Total Read Pairs	177305615	100.00%
Unmapped Read Pairs	12189478	6.87%
Mapped Read Pairs	148448055	83.72%
PCR Dup Read Pairs	44152929	24.90%
No-Dup Read Pairs	104295126	58.82%
No-Dup Cis Read Pairs	92814951	88.99%
No-Dup Trans Read Pairs	11480175	11.01%
No-Dup Valid Read Pairs (cis >= 1kb + trans)	48676466	46.67%
No-Dup Cis Read Pairs < 1kb	55618660	53.33%
No-Dup Cis Read Pairs >= 1kb	37196291	35.66%
No-Dup Cis Read Pairs >= 10kb	29111962	27.91%

Micro-C_WAPL_stats.txt		
Total Read Pairs	214235911	100.00%
Unmapped Read Pairs	12895170	6.02%
Mapped Read Pairs	181747934	84.84%
PCR Dup Read Pairs	63716382	29.74%
No-Dup Read Pairs	118031552	55.09%
No-Dup Cis Read Pairs	104164504	88.25%
No-Dup Trans Read Pairs	13867048	11.75%
No-Dup Valid Read Pairs (cis >= 1kb + trans)	62694490	53.12%
No-Dup Cis Read Pairs < 1kb	55337062	46.88%
No-Dup Cis Read Pairs >= 1kb	48827442	41.37%
No-Dup Cis Read Pairs >= 10kb	36829913	31.20%

Micro-C_WAPL_Auxin_stats.txt		
Total Read Pairs	206214204	100.00%
Unmapped Read Pairs	15188877	7.37%
Mapped Read Pairs	176527370	85.60%
PCR Dup Read Pairs	52671753	25.54%
No-Dup Read Pairs	123855617	60.06%
No-Dup Cis Read Pairs	115010469	92.86%
No-Dup Trans Read Pairs	8845148	7.14%
No-Dup Valid Read Pairs (cis >= 1kb + trans)	40033695	32.32%
No-Dup Cis Read Pairs < 1kb	83821922	67.68%
No-Dup Cis Read Pairs >= 1kb	31188547	25.18%
No-Dup Cis Read Pairs >= 10kb	23897537	19.29%

Micro-C_CTCF_stats.txt		
Total Read Pairs	689747470	100.00%
Unmapped Read Pairs	43175512	6.26%
Mapped Read Pairs	583770236	84.64%
PCR Dup Read Pairs	237014177	34.36%
No-Dup Read Pairs	346756059	50.27%
No-Dup Cis Read Pairs	306687371	88.44%
No-Dup Trans Read Pairs	40068688	11.56%
No-Dup Valid Read Pairs (cis >= 1kb + trans)	189318862	54.60%
No-Dup Cis Read Pairs < 1kb	157437197	45.40%
No-Dup Cis Read Pairs >= 1kb	149250174	43.04%
No-Dup Cis Read Pairs >= 10kb	114396433	32.99%

Micro-C_CTCF_Auxin_stats.txt		
Total Read Pairs	401439687	100.00%
Unmapped Read Pairs	27017372	6.73%
Mapped Read Pairs	341362534	85.03%
PCR Dup Read Pairs	109902928	27.38%
No-Dup Read Pairs	231459606	57.66%
No-Dup Cis Read Pairs	210111541	90.78%
No-Dup Trans Read Pairs	21348065	9.22%
No-Dup Valid Read Pairs (cis >= 1kb + trans)	109986584	47.52%
No-Dup Cis Read Pairs < 1kb	121473022	52.48%
No-Dup Cis Read Pairs >= 1kb	88638519	38.30%
No-Dup Cis Read Pairs >= 10kb	68460085	29.58%

Supplementary Table 4

Sequencing stats Repli-C: The amount and percentage of all read types per sample and replicate are provided. Stats corresponding to UNT and HU data sets used in Fig. 2 and Extended Data Fig. 2 and 4.

UNT_1_dedup.stats		
Total Read Pairs	585371674	100.00%
Unmapped Read Pairs	25253122	4.31%
Mapped Read Pairs	475226021	81.18%
PCR Dup Read Pairs	192828926	32.94%
No-Dup Read Pairs	282397095	48.24%
No-Dup Cis Read Pairs	128564588	45.53%
No-Dup Trans Read Pairs	153832507	54.47%
No-Dup Valid Read Pairs (cis >= 1kb + trans)	207011844	73.31%
No-Dup Cis Read Pairs < 1kb	75385251	26.69%
No-Dup Cis Read Pairs >= 1kb	53179337	18.83%
No-Dup Cis Read Pairs >= 10kb	35539548	12.58%

HU_1_dedup.stats		
Total Read Pairs	545430950	100.00%
Unmapped Read Pairs	27945157	5.12%
Mapped Read Pairs	444687446	81.53%
PCR Dup Read Pairs	177475128	32.54%
No-Dup Read Pairs	267212318	48.99%
No-Dup Cis Read Pairs	135404885	50.67%
No-Dup Trans Read Pairs	131807433	49.33%
No-Dup Valid Read Pairs (cis >= 1kb + trans)	183381513	68.63%
No-Dup Cis Read Pairs < 1kb	83830805	31.37%
No-Dup Cis Read Pairs >= 1kb	51574080	19.30%
No-Dup Cis Read Pairs >= 10kb	33166302	12.41%

UNT_2_dedup.stats		
Total Read Pairs	519915420	100.00%
Unmapped Read Pairs	22098515	4.25%
Mapped Read Pairs	427615021	82.25%
PCR Dup Read Pairs	174718497	33.61%
No-Dup Read Pairs	252896524	48.64%
No-Dup Cis Read Pairs	131821979	52.12%
No-Dup Trans Read Pairs	121074545	47.88%
No-Dup Valid Read Pairs (cis >= 1kb + trans)	179978480	71.17%
No-Dup Cis Read Pairs < 1kb	72918044	28.83%
No-Dup Cis Read Pairs >= 1kb	58903935	23.29%
No-Dup Cis Read Pairs >= 10kb	38278795	15.14%

HU_2_dedup.stats		
Total Read Pairs	449345895	100.00%
Unmapped Read Pairs	20996839	4.67%
Mapped Read Pairs	371867775	82.76%
PCR Dup Read Pairs	216585158	48.20%
No-Dup Read Pairs	155282617	34.56%
No-Dup Cis Read Pairs	98218949	63.25%
No-Dup Trans Read Pairs	57063668	36.75%
No-Dup Valid Read Pairs (cis >= 1kb + trans)	103848863	66.88%
No-Dup Cis Read Pairs < 1kb	51433754	33.12%
No-Dup Cis Read Pairs >= 1kb	46785195	30.13%
No-Dup Cis Read Pairs >= 10kb	28663881	18.46%

UNT_Auxin_1_dedup.stats		
Total Read Pairs	340455518	100.00%
Unmapped Read Pairs	14840036	4.36%
Mapped Read Pairs	276086350	81.09%
PCR Dup Read Pairs	91678384	26.93%
No-Dup Read Pairs	184407966	54.17%
No-Dup Cis Read Pairs	82980524	45.00%
No-Dup Trans Read Pairs	101427442	55.00%
No-Dup Valid Read Pairs (cis >= 1kb + trans)	133459927	72.37%
No-Dup Cis Read Pairs < 1kb	50948039	27.63%
No-Dup Cis Read Pairs >= 1kb	32032485	17.37%
No-Dup Cis Read Pairs >= 10kb	20691948	11.22%

HU_Auxin_1_dedup.stats		
Total Read Pairs	411752534	100.00%
Unmapped Read Pairs	19907090	4.83%
Mapped Read Pairs	333668704	81.04%
PCR Dup Read Pairs	142427639	34.59%
No-Dup Read Pairs	191241065	46.45%
No-Dup Cis Read Pairs	94766360	49.55%
No-Dup Trans Read Pairs	96474705	50.45%
No-Dup Valid Read Pairs (cis >= 1kb + trans)	131644958	68.84%
No-Dup Cis Read Pairs < 1kb	59596107	31.16%
No-Dup Cis Read Pairs >= 1kb	35170253	18.39%
No-Dup Cis Read Pairs >= 10kb	21468365	11.23%

UNT_Auxin_2_dedup.stats		
Total Read Pairs	490863447	100.00%
Unmapped Read Pairs	21648056	4.41%
Mapped Read Pairs	404818409	82.47%
PCR Dup Read Pairs	258050578	52.57%
No-Dup Read Pairs	146767831	29.90%
No-Dup Cis Read Pairs	87941232	59.92%
No-Dup Trans Read Pairs	58826599	40.08%
No-Dup Valid Read Pairs (cis >= 1kb + trans)	98474650	67.10%
No-Dup Cis Read Pairs < 1kb	48293181	32.90%
No-Dup Cis Read Pairs >= 1kb	39648051	27.01%
No-Dup Cis Read Pairs >= 10kb	23895630	16.28%

HU_Auxin_2_dedup.stats		
Total Read Pairs	617192282	100.00%
Unmapped Read Pairs	28377313	4.60%
Mapped Read Pairs	506939849	82.14%
PCR Dup Read Pairs	339028233	54.93%
No-Dup Read Pairs	167911616	27.21%
No-Dup Cis Read Pairs	96540225	57.49%
No-Dup Trans Read Pairs	71371391	42.51%
No-Dup Valid Read Pairs (cis >= 1kb + trans)	115090528	68.54%
No-Dup Cis Read Pairs < 1kb	52821088	31.46%
No-Dup Cis Read Pairs >= 1kb	43719137	26.04%
No-Dup Cis Read Pairs >= 10kb	25953660	15.46%

Supplementary Table 5

Sequencing stats Repli-C: The amount and percentage of all read types per sample and replicate are provided. Stats corresponding to UNT and HU data sets used in Fig. 2 and Extended Data Fig. 7.

UNT_WT_1_dedup.stats		
Total Read Pairs	176540053	100.00%
Unmapped Read Pairs	9390257	5.32%
Mapped Read Pairs	147285556	83.43%
PCR Dup Read Pairs	120245608	68.11%
No-Dup Read Pairs	27039948	15.32%
No-Dup Cis Read Pairs	21458925	79.36%
No-Dup Trans Read Pairs	5581023	20.64%
No-Dup Valid Read Pairs (cis >= 1kb + trans)	15290770	56.55%
No-Dup Cis Read Pairs < 1kb	11749178	43.45%
No-Dup Cis Read Pairs >= 1kb	9709747	35.91%
No-Dup Cis Read Pairs >= 10kb	5431939	20.09%

HU_WT_1_dedup.stats		
Total Read Pairs	170215404	100.00%
Unmapped Read Pairs	8645765	5.08%
Mapped Read Pairs	141397492	83.07%
PCR Dup Read Pairs	115448759	67.83%
No-Dup Read Pairs	25948733	15.24%
No-Dup Cis Read Pairs	19650976	75.73%
No-Dup Trans Read Pairs	6297757	24.27%
No-Dup Valid Read Pairs (cis >= 1kb + trans)	15276230	58.87%
No-Dup Cis Read Pairs < 1kb	10672503	41.13%
No-Dup Cis Read Pairs >= 1kb	8978473	34.60%
No-Dup Cis Read Pairs >= 10kb	4826511	18.60%

UNT_WT_2_dedup.stats		
Total Read Pairs	193077797	100.00%
Unmapped Read Pairs	9839014	5.10%
Mapped Read Pairs	159686591	82.71%
PCR Dup Read Pairs	128607198	66.61%
No-Dup Read Pairs	31079393	16.10%
No-Dup Cis Read Pairs	22294320	71.73%
No-Dup Trans Read Pairs	8785073	28.27%
No-Dup Valid Read Pairs (cis >= 1kb + trans)	18139586	58.37%
No-Dup Cis Read Pairs < 1kb	12939807	41.63%
No-Dup Cis Read Pairs >= 1kb	9354513	30.10%
No-Dup Cis Read Pairs >= 10kb	5168604	16.63%

HU_WT_2_dedup.stats		
Total Read Pairs	158596114	100.00%
Unmapped Read Pairs	8206856	5.17%
Mapped Read Pairs	132142042	83.32%
PCR Dup Read Pairs	113867556	71.80%
No-Dup Read Pairs	18274486	11.52%
No-Dup Cis Read Pairs	14633785	80.08%
No-Dup Trans Read Pairs	3640701	19.92%
No-Dup Valid Read Pairs (cis >= 1kb + trans)	10122646	55.39%
No-Dup Cis Read Pairs < 1kb	8151840	44.61%
No-Dup Cis Read Pairs >= 1kb	6481945	35.47%
No-Dup Cis Read Pairs >= 10kb	3332022	18.23%

UNT_WT_3_dedup.stats		
Total Read Pairs	871833535	100.00%
Unmapped Read Pairs	44103048	5.06%
Mapped Read Pairs	721098166	82.71%
PCR Dup Read Pairs	317356593	36.40%
No-Dup Read Pairs	403741573	46.31%
No-Dup Cis Read Pairs	225882102	55.95%
No-Dup Trans Read Pairs	177859471	44.05%
No-Dup Valid Read Pairs (cis >= 1kb + trans)	273088191	67.64%
No-Dup Cis Read Pairs < 1kb	130653382	32.36%
No-Dup Cis Read Pairs >= 1kb	95228720	23.59%
No-Dup Cis Read Pairs >= 10kb	68335897	16.93%

HU_WT_3_dedup.stats		
Total Read Pairs	642217089	100.00%
Unmapped Read Pairs	38758821	6.04%
Mapped Read Pairs	525020670	81.75%
PCR Dup Read Pairs	364798475	56.80%
No-Dup Read Pairs	160222195	24.95%
No-Dup Cis Read Pairs	106208170	66.29%
No-Dup Trans Read Pairs	54014025	33.71%
No-Dup Valid Read Pairs (cis >= 1kb + trans)	101389146	63.28%
No-Dup Cis Read Pairs < 1kb	58833049	36.72%
No-Dup Cis Read Pairs >= 1kb	47375121	29.57%
No-Dup Cis Read Pairs >= 10kb	32725938	20.43%

UNT_WT_4_dedup.stats		
Total Read Pairs	881670697	100.00%
Unmapped Read Pairs	40212108	4.56%
Mapped Read Pairs	728598531	82.64%
PCR Dup Read Pairs	317060240	35.96%
No-Dup Read Pairs	411538291	46.68%
No-Dup Cis Read Pairs	197667718	48.03%
No-Dup Trans Read Pairs	213870573	51.97%
No-Dup Valid Read Pairs (cis >= 1kb + trans)	283113173	68.79%
No-Dup Cis Read Pairs < 1kb	128425118	31.21%
No-Dup Cis Read Pairs >= 1kb	69242600	16.83%
No-Dup Cis Read Pairs >= 10kb	47901341	11.64%

HU_WT_4_dedup.stats		
Total Read Pairs	749999426	100.00%
Unmapped Read Pairs	39863137	5.32%
Mapped Read Pairs	601537470	80.21%
PCR Dup Read Pairs	402400362	53.65%
No-Dup Read Pairs	199137108	26.55%
No-Dup Cis Read Pairs	103439412	51.94%
No-Dup Trans Read Pairs	95697696	48.06%
No-Dup Valid Read Pairs (cis >= 1kb + trans)	127327213	63.94%
No-Dup Cis Read Pairs < 1kb	71809895	36.06%
No-Dup Cis Read Pairs >= 1kb	31629517	15.88%
No-Dup Cis Read Pairs >= 10kb	21569536	10.83%

Supplementary Table 6

Sequencing stats Repli-C: The amount and percentage of all read types per sample and replicate are provided.
Stats corresponding to UNT and HU data sets used in Fig. 2 and Extended Data Fig. 7.

UNT_4E_1_dedup.stats		
Total Read Pairs	206932208	100.00%
Unmapped Read Pairs	11763480	5.68%
Mapped Read Pairs	171072955	82.67%
PCR Dup Read Pairs	115552821	55.84%
No-Dup Read Pairs	55520134	26.83%
No-Dup Cis Read Pairs	39466298	71.08%
No-Dup Trans Read Pairs	16053836	28.92%
No-Dup Valid Read Pairs (cis >= 1kb + trans)	33912225	61.08%
No-Dup Cis Read Pairs < 1kb	21607909	38.92%
No-Dup Cis Read Pairs >= 1kb	17858389	32.17%
No-Dup Cis Read Pairs >= 10kb	9756733	17.57%

HU_4E_1_dedup.stats		
Total Read Pairs	195681759	100.00%
Unmapped Read Pairs	11177725	5.71%
Mapped Read Pairs	162895741	83.25%
PCR Dup Read Pairs	134935570	68.96%
No-Dup Read Pairs	27960171	14.29%
No-Dup Cis Read Pairs	22483388	80.41%
No-Dup Trans Read Pairs	5476783	19.59%
No-Dup Valid Read Pairs (cis >= 1kb + trans)	14763373	52.80%
No-Dup Cis Read Pairs < 1kb	13196798	47.20%
No-Dup Cis Read Pairs >= 1kb	9286590	33.21%
No-Dup Cis Read Pairs >= 10kb	4700131	16.81%

UNT_4E_2_dedup.stats		
Total Read Pairs	191368973	100.00%
Unmapped Read Pairs	10436922	5.45%
Mapped Read Pairs	159370346	83.28%
PCR Dup Read Pairs	127074553	66.40%
No-Dup Read Pairs	32295793	16.88%
No-Dup Cis Read Pairs	25776737	79.81%
No-Dup Trans Read Pairs	6519056	20.19%
No-Dup Valid Read Pairs (cis >= 1kb + trans)	18487381	57.24%
No-Dup Cis Read Pairs < 1kb	13808412	42.76%
No-Dup Cis Read Pairs >= 1kb	11968325	37.06%
No-Dup Cis Read Pairs >= 10kb	6271413	19.42%

HU_4E_2_dedup.stats		
Total Read Pairs	130001373	100.00%
Unmapped Read Pairs	7553974	5.81%
Mapped Read Pairs	108815724	83.70%
PCR Dup Read Pairs	93527603	71.94%
No-Dup Read Pairs	15288121	11.76%
No-Dup Cis Read Pairs	13424594	87.81%
No-Dup Trans Read Pairs	1863527	12.19%
No-Dup Valid Read Pairs (cis >= 1kb + trans)	7474312	48.89%
No-Dup Cis Read Pairs < 1kb	7813809	51.11%
No-Dup Cis Read Pairs >= 1kb	5610785	36.70%
No-Dup Cis Read Pairs >= 10kb	2707607	17.71%

UNT_4E_3_dedup.stats		
Total Read Pairs	762418002	100.00%
Unmapped Read Pairs	41903058	5.50%
Mapped Read Pairs	625572646	82.05%
PCR Dup Read Pairs	322373158	42.28%
No-Dup Read Pairs	303199488	39.77%
No-Dup Cis Read Pairs	171718099	56.64%
No-Dup Trans Read Pairs	131481389	43.36%
No-Dup Valid Read Pairs (cis >= 1kb + trans)	193419437	63.79%
No-Dup Cis Read Pairs < 1kb	109780051	36.21%
No-Dup Cis Read Pairs >= 1kb	61938048	20.43%
No-Dup Cis Read Pairs >= 10kb	43311015	14.28%

HU_4E_3_dedup.stats		
Total Read Pairs	687530785	100.00%
Unmapped Read Pairs	41315651	6.01%
Mapped Read Pairs	560457709	81.52%
PCR Dup Read Pairs	408845934	59.47%
No-Dup Read Pairs	151611775	22.05%
No-Dup Cis Read Pairs	97366415	64.22%
No-Dup Trans Read Pairs	54245360	35.78%
No-Dup Valid Read Pairs (cis >= 1kb + trans)	89820059	59.24%
No-Dup Cis Read Pairs < 1kb	61791716	40.76%
No-Dup Cis Read Pairs >= 1kb	35574699	23.46%
No-Dup Cis Read Pairs >= 10kb	23042485	15.20%

UNT_4E_4_dedup.stats		
Total Read Pairs	614898892	100.00%
Unmapped Read Pairs	31383264	5.10%
Mapped Read Pairs	503383982	81.86%
PCR Dup Read Pairs	303383579	49.34%
No-Dup Read Pairs	200000403	32.53%
No-Dup Cis Read Pairs	116419936	58.21%
No-Dup Trans Read Pairs	83580467	41.79%
No-Dup Valid Read Pairs (cis >= 1kb + trans)	128949019	64.47%
No-Dup Cis Read Pairs < 1kb	71051384	35.53%
No-Dup Cis Read Pairs >= 1kb	45368552	22.68%
No-Dup Cis Read Pairs >= 10kb	31353546	15.68%

HU_4E_4_dedup.stats		
Total Read Pairs	709071388	100.00%
Unmapped Read Pairs	40603328	5.73%
Mapped Read Pairs	582465681	82.14%
PCR Dup Read Pairs	492896871	69.51%
No-Dup Read Pairs	89568810	12.63%
No-Dup Cis Read Pairs	61446059	68.60%
No-Dup Trans Read Pairs	28122751	31.40%
No-Dup Valid Read Pairs (cis >= 1kb + trans)	53885807	60.16%
No-Dup Cis Read Pairs < 1kb	35683003	39.84%
No-Dup Cis Read Pairs >= 1kb	25763056	28.76%
No-Dup Cis Read Pairs >= 10kb	16570956	18.50%

Supplementary Figure 1

Figure 1

f

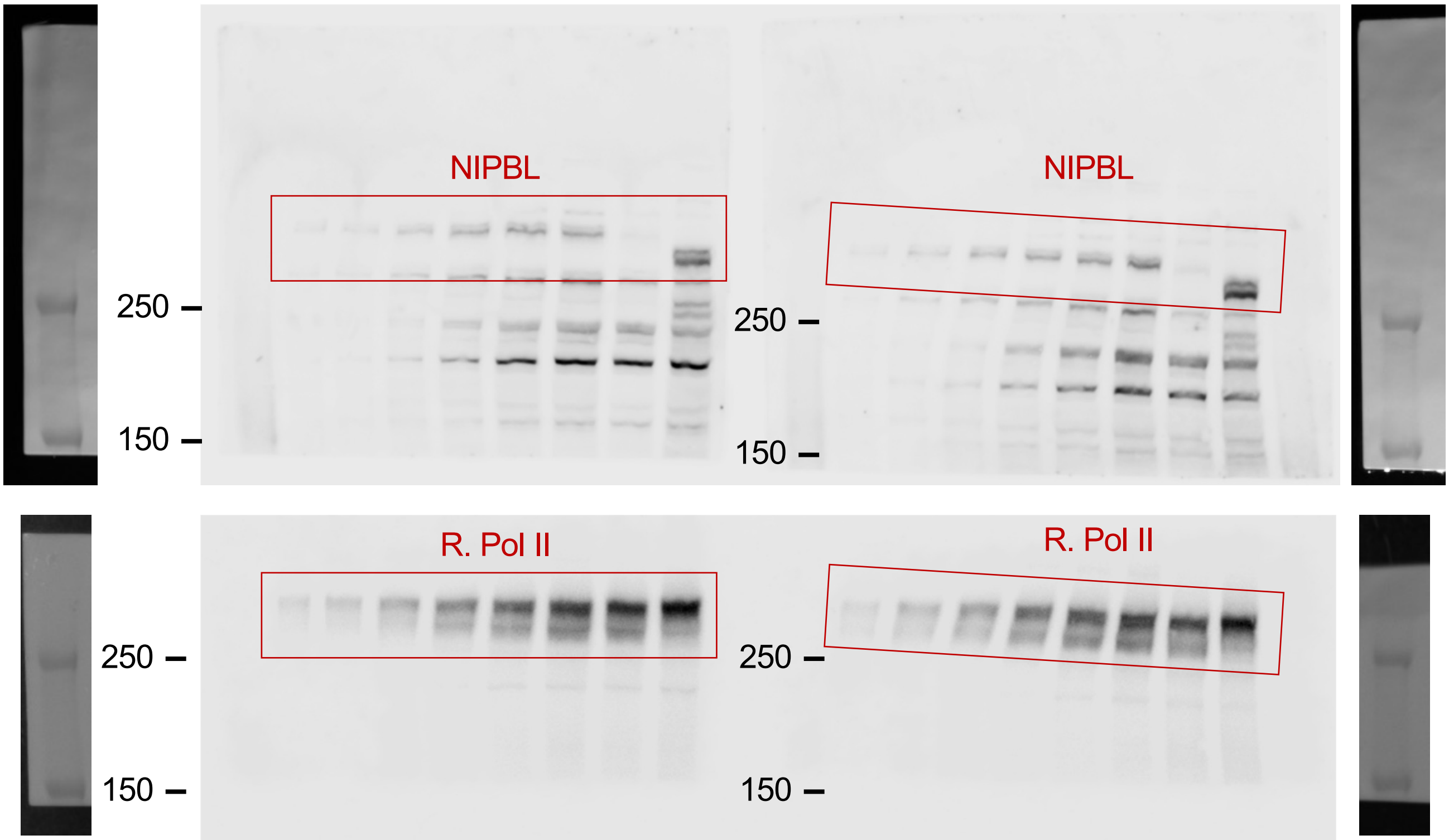

Supplementary Figure 1: Uncropped immunoblots corresponding to Fig. 1f. Uncropped membranes with molecular weight markers are displayed. Red boxes indicate the cropped regions shown in the Figure. Data are representative of three independent biological replicates.

ED Figure 3

a

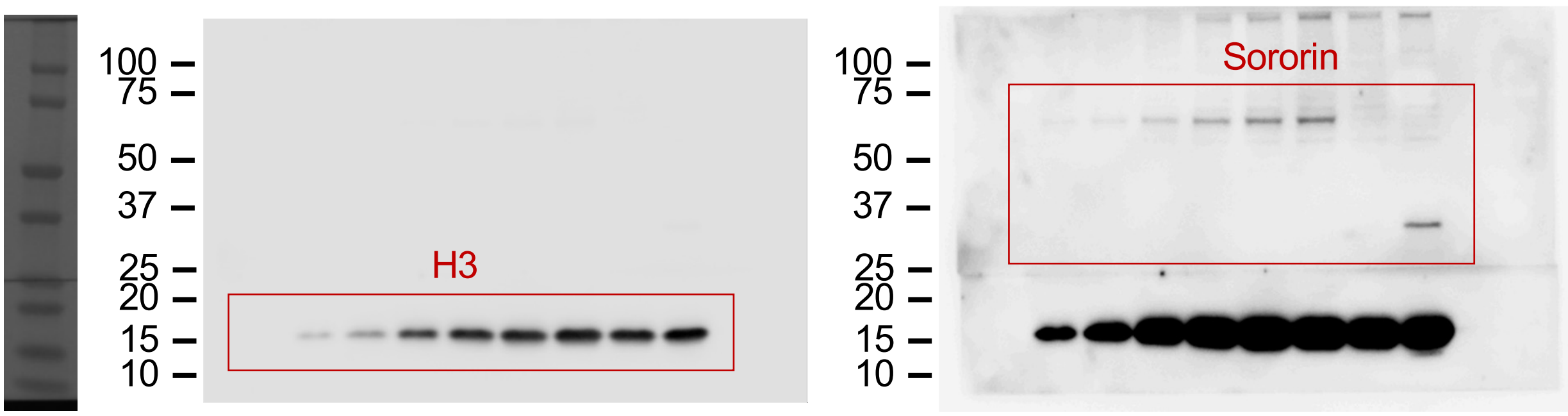

e

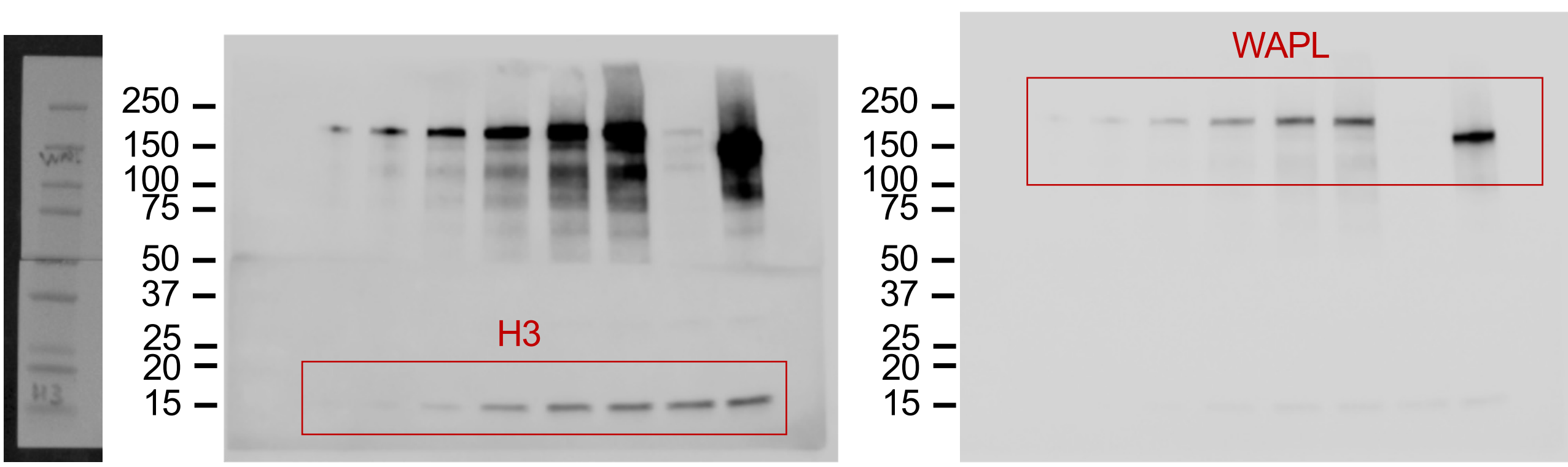

k

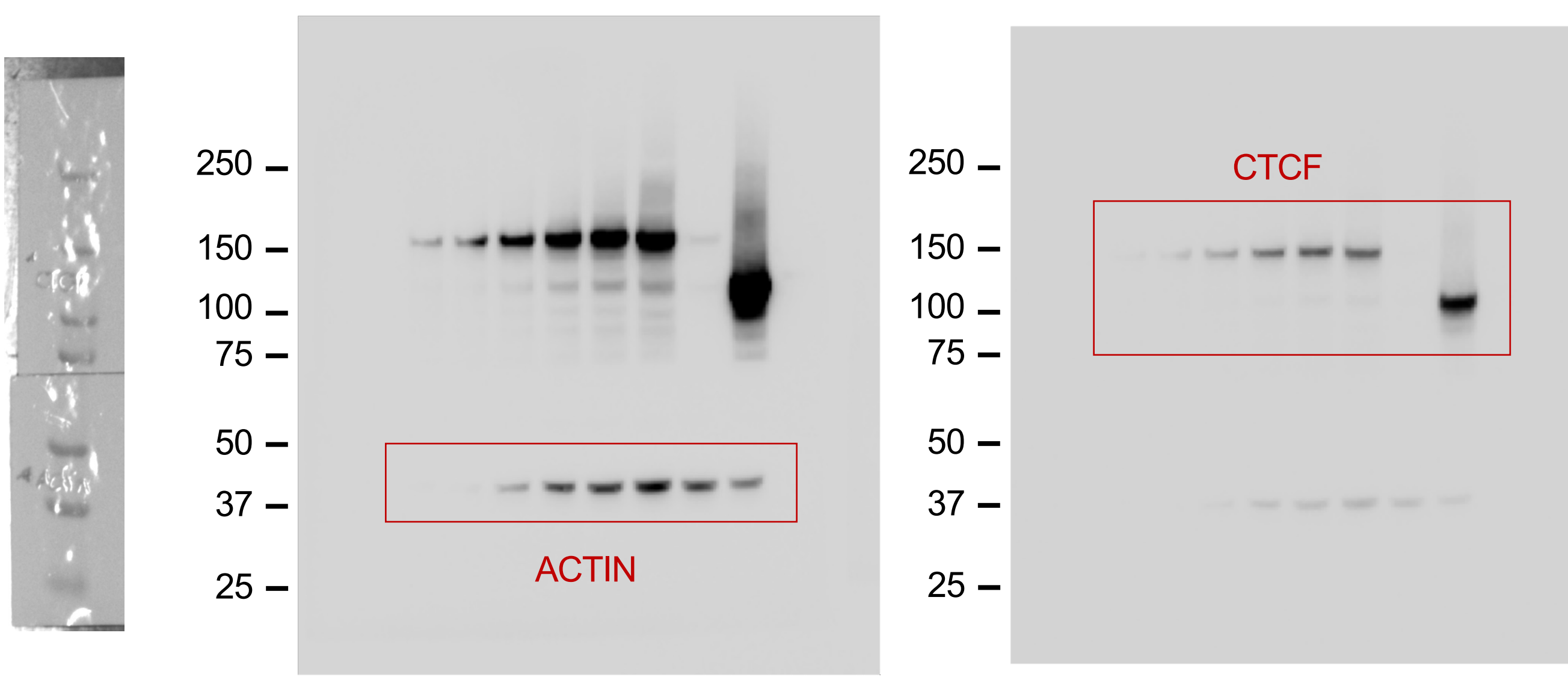

Uncropped immunoblots corresponding to ED Fig. 3a, e and k. Uncropped membranes with molecular weight markers are displayed. Red boxes indicate the cropped regions shown in the Figure. Data are representative of three independent biological replicates.

ED Figure 4

a

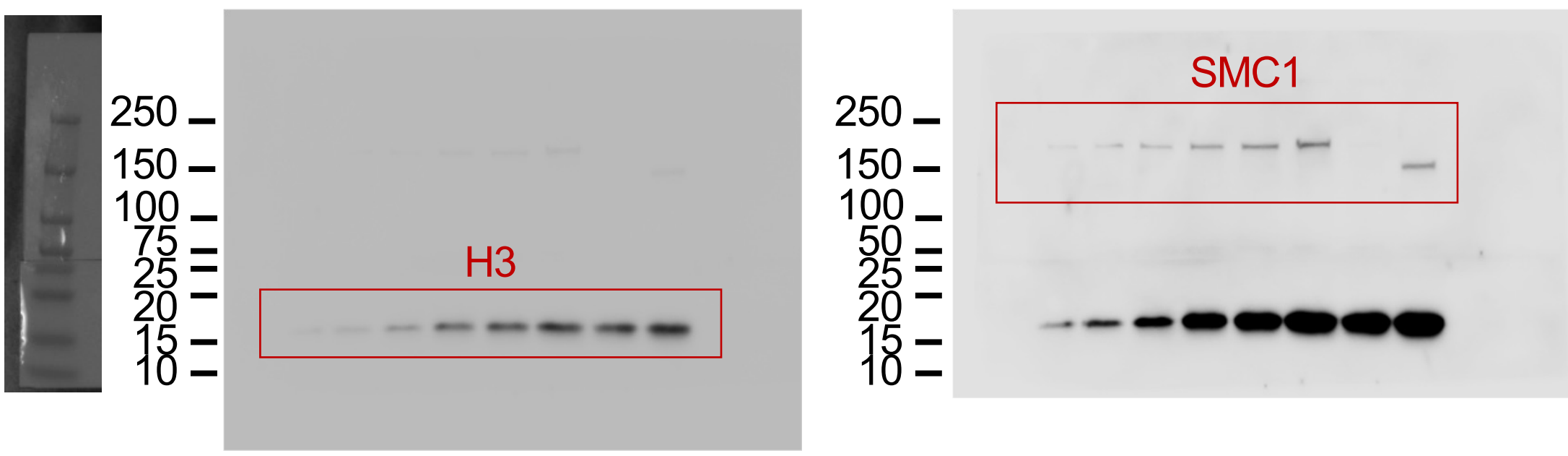

b

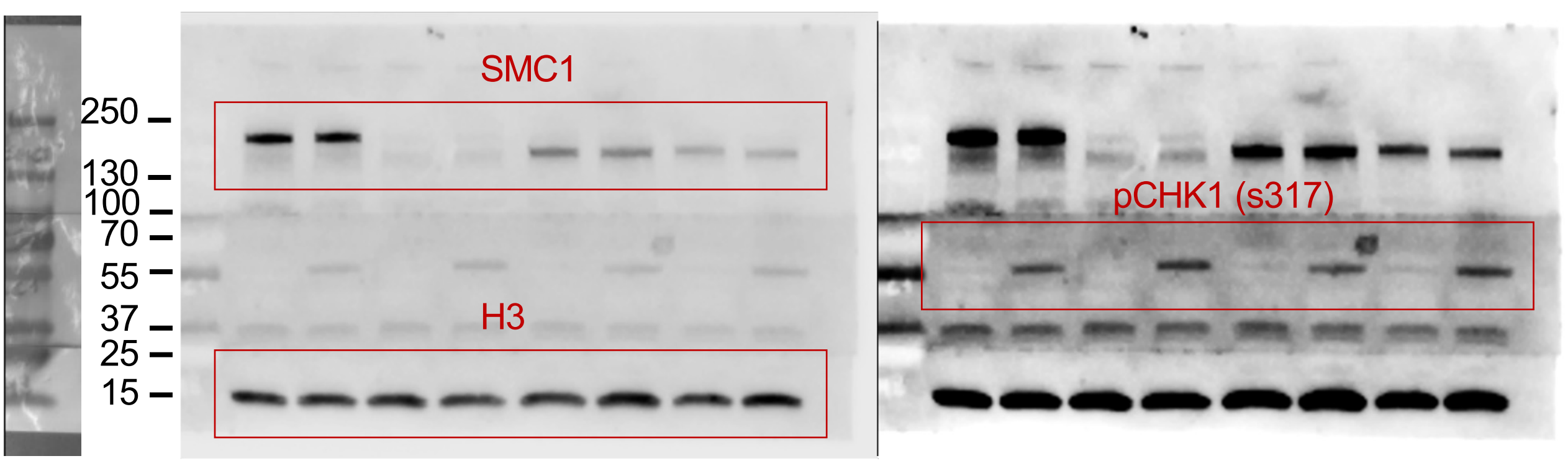

Uncropped immunoblots corresponding to ED Fig. 4a and b. Uncropped membranes with molecular weight markers are displayed. Red boxes indicate the cropped regions shown in the Figure. Data are representative of three independent biological replicates.

ED Figure 5

c

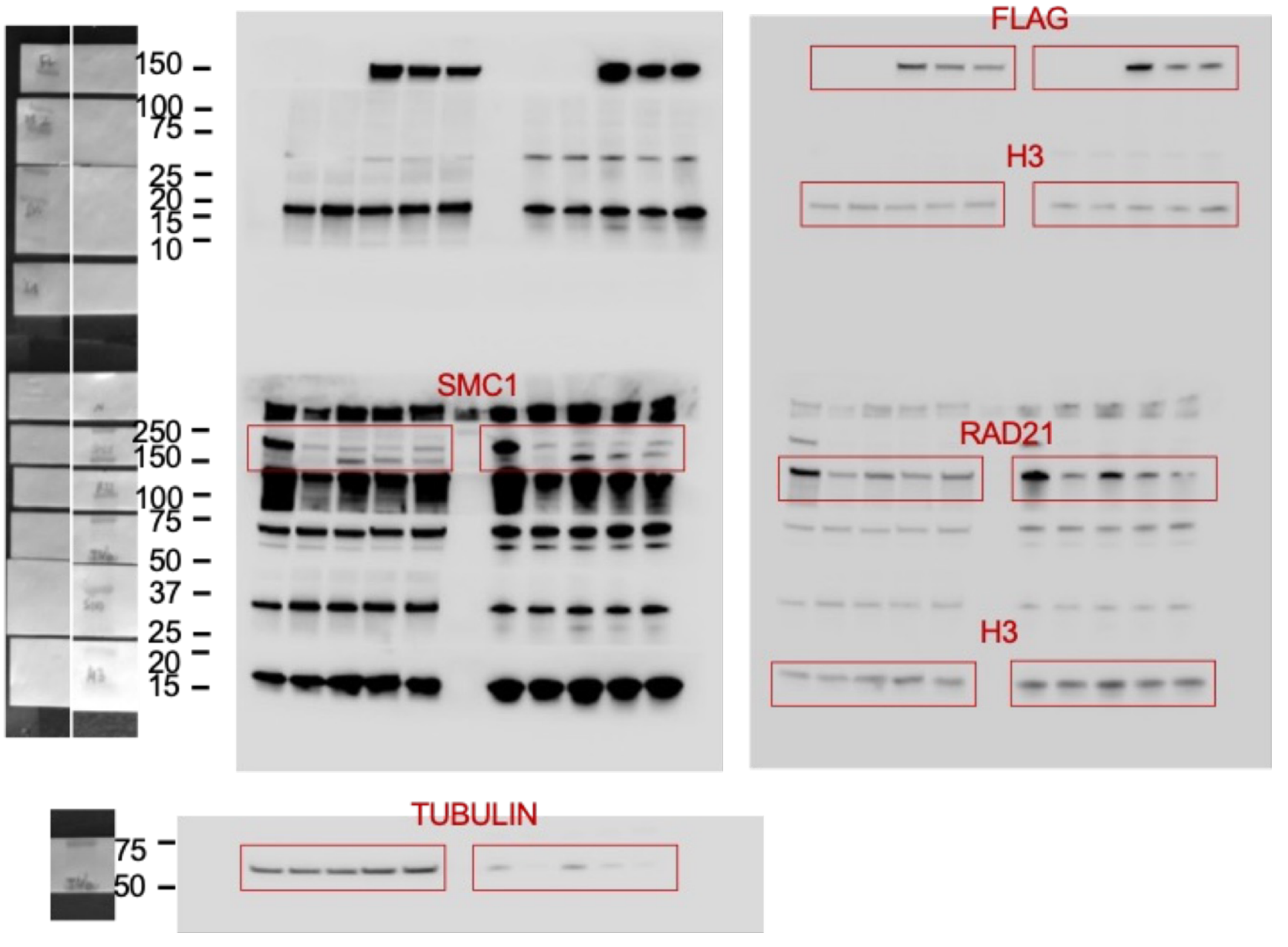

d

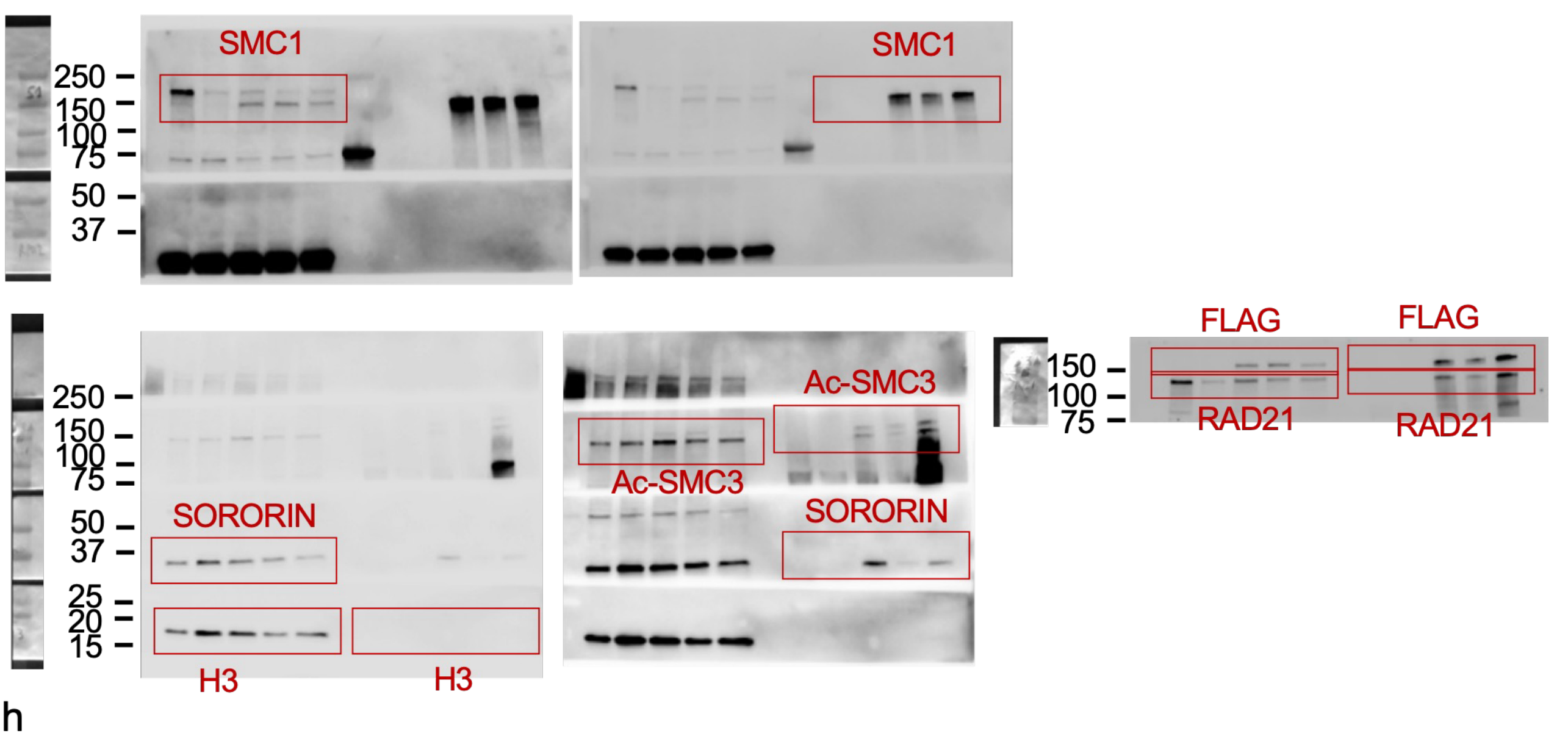

h

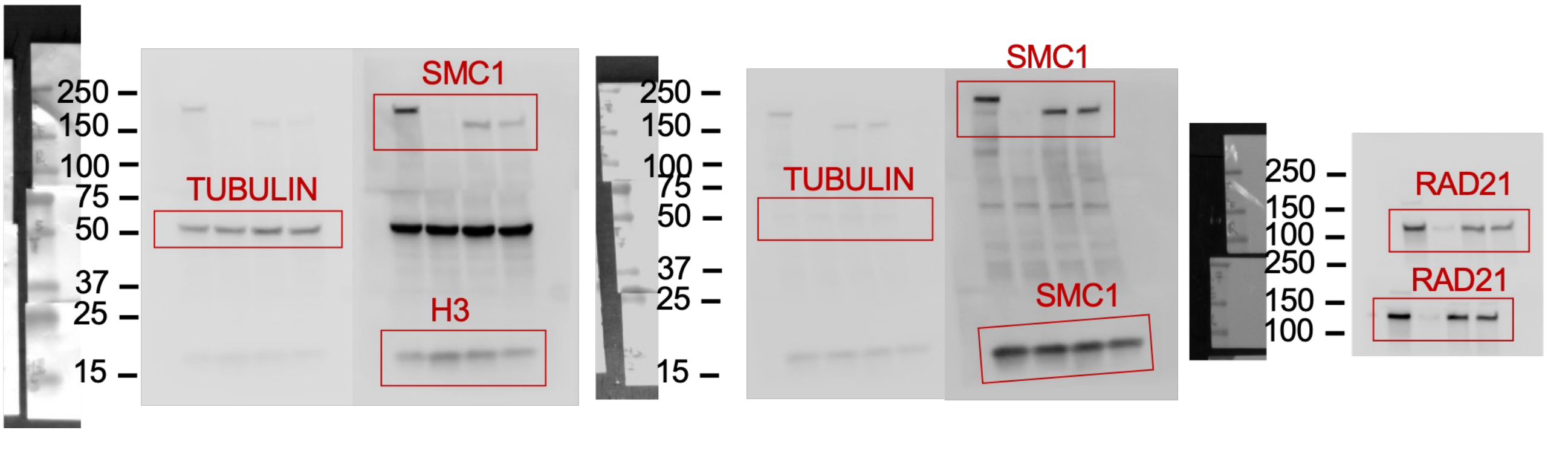

Uncropped immunoblots corresponding to ED Fig. 5c, d and h. Uncropped membranes with molecular weight markers are displayed. Red boxes indicate the cropped regions shown in the Figure. Data are representative of three independent biological replicates.

ED Figure 8

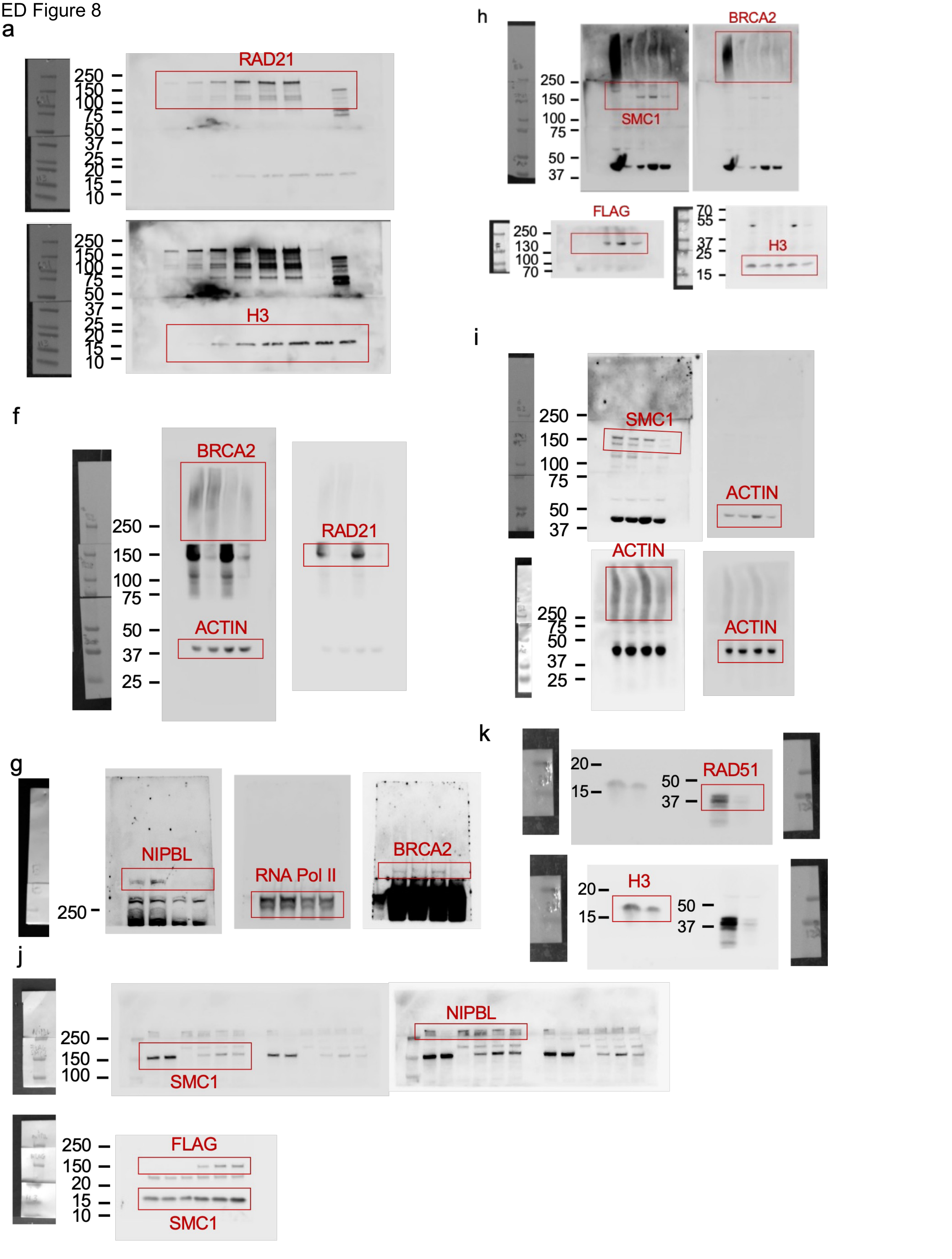

Uncropped immunoblots corresponding to ED Fig. 8a and f-k. Uncropped membranes with molecular weight markers are displayed. Red boxes indicate the cropped regions shown in the Figure. Data are representative of three independent biological replicates.

ED Figure 9

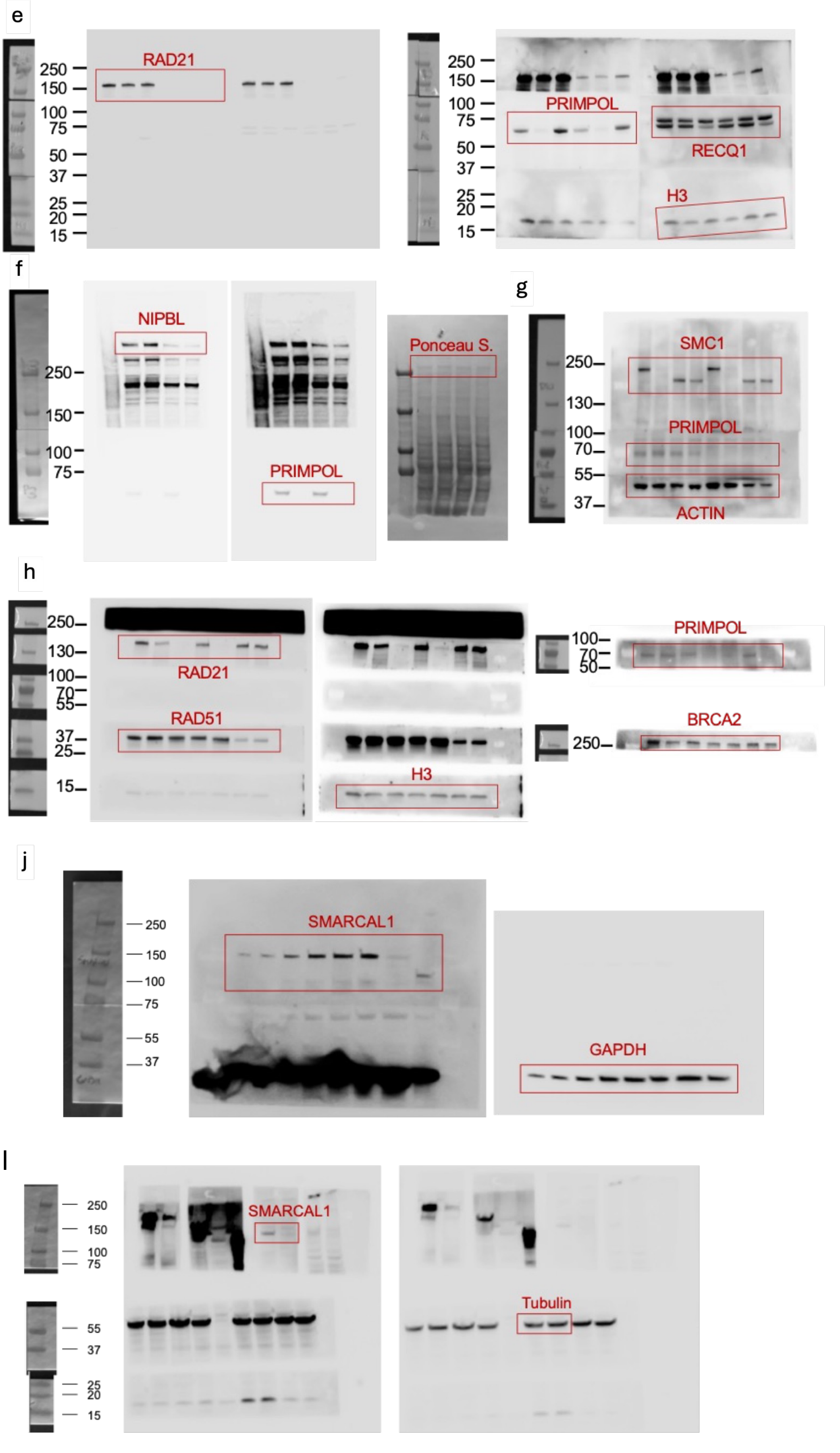

Uncropped immunoblots corresponding to ED Fig. 9e-h, j and l. Uncropped membranes with molecular weight markers are displayed. Red boxes indicate the cropped regions shown in the Figure. Data are representative of three independent biological replicates.

Supplementary Figure 2

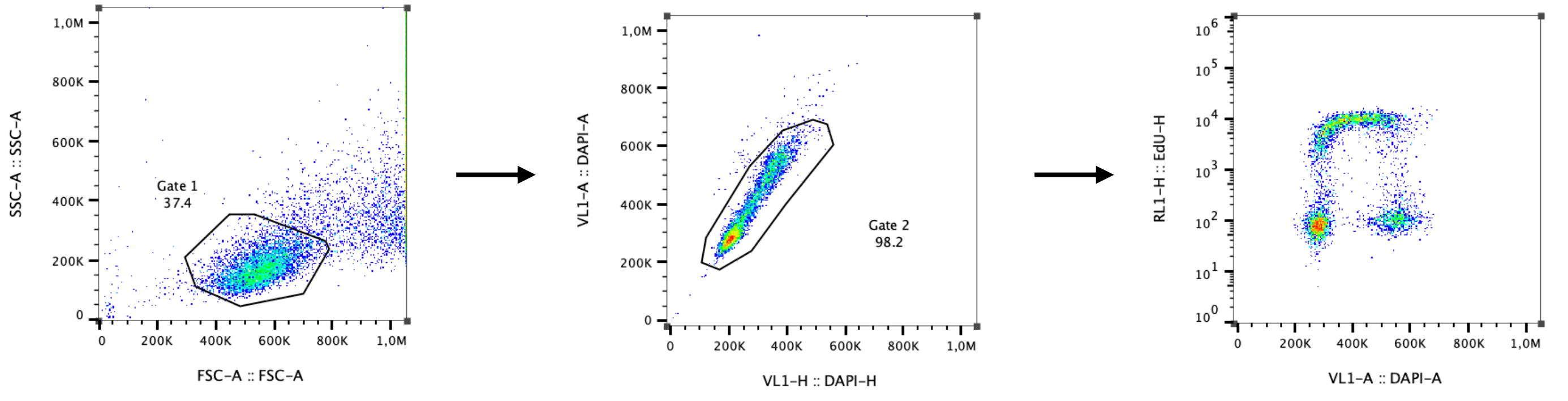

Supplementary Figure 2: Flow cytometry gating strategy. Cells were gated based on size and complexity (FSC-A vs. SSC-A). Cell doublets and aggregates were subsequently excluded using DAPI-H vs. DAPI-A. The resulting single-cell population was then either analyzed for DAPI-A and EdU-H intensities to generate the plots shown in Extended Data Fig. 2b, 3b, 3f, 3l, 5e and 5g.
