# Supplementary material for: Cohesin reshapes replication fork contacts to aid fork slowing and reversal
Source: Nature. 2026 Sep 16;658(8137):1057–65. doi: 10.1038/s41586-026-11034-0 (PMC13686946; doi:10.1038/s41586-026-11034-0)
Supplement: Supplementary file 2 [file 41586_2026_11034_MOESM2_ESM.pdf]

nature portfolio

Corresponding author(s): Massimo Lopes

Last updated by author(s): Aug 6, 2026

Reporting Summary

Nature Portfolio wishes to improve the reproducibility of the work that we publish. This form provides structure for consistency and transparency in reporting. For further information on Nature Portfolio policies, see our Editorial Policies and the Editorial Policy Checklist.

Statistics

For all statistical analyses, confirm that the following items are present in the figure legend, table legend, main text, or Methods section.

n/a	Confirmed	
[ ]	[x]	The exact sample size (*n*) for each experimental group/condition, given as a discrete number and unit of measurement
[ ]	[x]	A statement on whether measurements were taken from distinct samples or whether the same sample was measured repeatedly
[ ]	[x]	The statistical test(s) used AND whether they are one- or two-sided<br>*Only common tests should be described solely by name; describe more complex techniques in the Methods section.*
[x]	[ ]	A description of all covariates tested
[ ]	[x]	A description of any assumptions or corrections, such as tests of normality and adjustment for multiple comparisons
[ ]	[x]	A full description of the statistical parameters including central tendency (e.g. means) or other basic estimates (e.g. regression coefficient) AND variation (e.g. standard deviation) or associated estimates of uncertainty (e.g. confidence intervals)
[ ]	[x]	For null hypothesis testing, the test statistic (e.g. *F*, *t*, *r*) with confidence intervals, effect sizes, degrees of freedom and *P* value noted<br>*Give P values as exact values whenever suitable.*
[x]	[ ]	For Bayesian analysis, information on the choice of priors and Markov chain Monte Carlo settings
[x]	[ ]	For hierarchical and complex designs, identification of the appropriate level for tests and full reporting of outcomes
[x]	[ ]	Estimates of effect sizes (e.g. Cohen's *d*, Pearson's *r*), indicating how they were calculated

Our web collection on statistics for biologists contains articles on many of the points above.

Software and code

Policy information about availability of computer code

Data collection	No code has been generated in this study.<br>The following instruments has been used for data collection in the study:<br>Orbitrap Exploris 480 mass spectrometer (Thermo Fisher)<br>ACQUITY UPLC M-Class System (Waters)<br>Leica DM6 B microscope (Leica)<br>Attune NxT Flow Cytometer (Thermo Fisher)<br>Leica DM6000 microscope (Leica)<br>Talos 120 transmission electron microscope (FEI)<br>CMOS camera BM-Ceta (Thermo Fisher Scientific)<br>Plate reader (Company not specified in the text)<br>Illumina NovaSeq X Plus
Data analysis	No code was generated in this study.<br>The following software were used to analyse data:<br>R (4.3.3)<br>PROSTAR package (1.34.6)<br>Perseus (1.6.10.50)<br>GraphPad Prism (10)<br>Cell Profiler (4.2.8)

FlowJo (10.0.8)
LAS AF software (3.2)
ImageJ (1.54r)
MAPS 3 software (3.28)
MAPS offline viewer (3.28)
Fiji (1.54r)
Trim Galore (2.3.0)
BWA-MEM (0.7.17)
pairtools suite (1.1.3)
Juicer Tools (1.22.01)
HiCExplorer (3.7.6)
Chromosight (1.6.3)
FUN (Liu, Yang et al. Science, 2024)
Format:
coolpuppy (1.1.0)

For manuscripts utilizing custom algorithms or software that are central to the research but not yet described in published literature, software must be made available to editors and reviewers. We strongly encourage code deposition in a community repository (e.g. GitHub). See the Nature Portfolio guidelines for submitting code & software for further information.

Data

Policy information about availability of data

All manuscripts must include a data availability statement. This statement should provide the following information, where applicable:

- Accession codes, unique identifiers, or web links for publicly available datasets
- A description of any restrictions on data availability
- For clinical datasets or third party data, please ensure that the statement adheres to our policy

Micro-C and Repli-C sequencing data presented in this study are deposited and publicly available at the NCBI Gene Expression Omnibus (GEO) databases under the following accession IDs: GSE311634 and GSE311782.
The raw proteomic data has been deposited and is publicly available in ProteomeXchange under the following accession ID: PXD071150.

Research involving human participants, their data, or biological material

Policy information about studies with human participants or human data. See also policy information about sex, gender (identity/presentation), and sexual orientation and race, ethnicity and racism.

Reporting on sex and gender	n/a
Reporting on race, ethnicity, or other socially relevant groupings	n/a
Population characteristics	n/a
Recruitment	n/a
Ethics oversight	n/a

Note that full information on the approval of the study protocol must also be provided in the manuscript.

Field-specific reporting

Please select the one below that is the best fit for your research. If you are not sure, read the appropriate sections before making your selection.

[x] Life sciences [ ] Behavioural & social sciences [ ] Ecological, evolutionary & environmental sciences

For a reference copy of the document with all sections, see nature.com/documents/nr-reporting-summary-flat.pdf

Life sciences study design

All studies must disclose on these points even when the disclosure is negative.

Sample size	No statistical method was used to determine sample size. Sample size were chosen for all experiments according to throughput and technical possibilities on the individual assays. For electron microscopy, n>70; DNA fibers, n>100; Immunofluorescence of fixed cells, n>100; flow cytometry n=10000 in the final gate. Those numbers were chosen to obtain statistical power, in conformity to accepted standard sample size in a number of previous publications using the same approaches.
Data exclusions	In SIRF experiments (where indicated), EdU-negative cells were excluded from the analyses to focus our observation on S-phase.

Replication	For all experiments, the number of biological replicates is indicated and, without any exception, reproduced trends of the representative data shown in the figures.
Randomization	Randomization was not relevant for this study, as experiments were performed on cultured cell lines derived from a homogenous population.
Blinding	Blinding was only applied for DNA fiber and Electron Microscopy analysis since they are the only two assays analysed entirely by hand. For other assays such as immunofluoresence, fields of view were selected arbitrarily to ensure representative sampling across the entire coverslip.

Reporting for specific materials, systems and methods

We require information from authors about some types of materials, experimental systems and methods used in many studies. Here, indicate whether each material, system or method listed is relevant to your study. If you are not sure if a list item applies to your research, read the appropriate section before selecting a response.

Materials & experimental systems

n/a	Involved in the study	
☐	☒	Antibodies
☐	☒	Eukaryotic cell lines
☒	☐	Palaeontology and archaeology
☒	☐	Animals and other organisms
☒	☐	Clinical data
☒	☐	Dual use research of concern
☒	☐	Plants

Methods

n/a	Involved in the study	
☒	☐	ChIP-seq
☐	☒	Flow cytometry
☒	☐	MRI-based neuroimaging

Antibodies

Antibodies used	SMC1 #1 (Rb) Not commercially available; Ana Losada Lab, (Spanish Cancer Research Centre, CNIO) Reference #42<br>SMC1 #2 (Rb) Thermo Fisher, PA5-29122<br>NIPBL (Rb) Not commercially available; Ana Losada Lab, (Spanish Cancer Research Centre, CNIO) Reference #42<br>RAD21 (Rb) Abcam, ab217678<br>Sororin (Rb) Not commercially available; Ana Losada Lab, (Spanish Cancer Research Centre, CNIO) Reference #67<br>WAPL (Rb) Cell Signaling, 77428<br>CTCF (Rb) Cell Signaling, 3418<br>Ac-SMC3 (Ms) Merck, MABE1925<br>RNA Pol II RPB1 (Ms) BioLegend, 920204<br>H3 (Rb) Abcam, ab1791<br>ACTIN (Ms Sigma-Aldrich, A2228<br>FLAG (Ms) Merck, F3165<br>PRIMPOL (Rat) Not commercially available; Juan Méndez Lab, (Spanish Cancer Research Centre, CNIO) Reference #68<br>PRIMPOL (Rb) Proteintech, 29824-1-AP<br>MCM2 (Ms) Thermos Fisher, MA5-15923<br>RECQ1 (Rb) Abcam, ab172607<br>RAD51 (Rb) BioAcademia, 70-002<br>BRCA2 (Ms) Sigma-Aldrich, OP95<br>Tubulin (Ms) Sigma-Aldrich, T6199<br>SMARCAL1 (Rb), Abcam, ab154226<br>GAPDH (Ms), Sigma-Aldrich, MAB374<br>phospho-H2AX (Ser 139) (Rb), Cell Signaling, 9718<br>phospho-ATM (Ser 1981) (Ms), Thermo Fisher, 200-301-400<br>phospho-Chk1 (Ser 317) (Rb) Cell Signaling, 12302<br>Biotin (Ms) Jackson ImmunoResearch, 200-002-211<br>Digoxigenin (Ms) MBL, M227-3<br>BrdU (CldU; Rat) Abcam, ab6326<br>BrdU (IdU; Ms) Becton Dickinson, 347580<br>Alexa 488 (Ms IgG1) Invitrogen, A-21121<br>Alexa 555 (Rat) Invitrogen, A-21434<br>Alexa 488 (Ms) Invitrogen, A-11001<br>Alexa 488 (Rb) Invitrogen, A-11034<br>Alexa 555 (Ms) Invitrogen, A-21422<br>Alexa 555 (Rb) Invitrogen, A-21428<br>Alexa 647 (Ms) Invitrogen, A-31571<br>F(ab')2 Alexa 647 (Rb) Invitrogen, A-21246<br>ECL IgG, HRP-linked (Ms) Sigma-Aldrich, NA934<br>ECL IgG, HRP-linked (Rb) Sigma-Aldrich, GENA931<br>IgG, Cross-Adsorbed, HRP (Rat) Thermo Fisher, A10549
Validation	All antibodies used in this study are commercially available and have been validated by the manufacturer, except for the SMC1 #1, the NIPBL, Sororin and PRIMPOL antibodies which have been generated and validated by the indicated laboratory and have been reported in the indicated publications included in the reference list of the manuscript.

Eukaryotic cell lines

Policy information about cell lines and Sex and Gender in Research

Cell line source(s)	HCT116 was purchased from ATCC; HCT116 OsTIR (F74G), mAID-mClover-RAD21 HCT116 OsTIR (F74G), WAPL-mClover-mAID HCT116 OsTIR (F74G) and CTCF-mClover-mAID HCT116 OsTIR (F74G) were generated in the laboratory of Dr. Masato Kanemaki. mAID-mClover-NIBPL HCT116 OsTIR (F74G), mAID-mClover-Sororin HCT116 OsTIR (F74G), SMC1-mClover-mAID HCT116 OsTIR (F74G), SMC1-mClover-mAID HCT116 OsTIR (F74G) + SMC1WT-3xFLAG, SMC1-mClover-mAID HCT116 OsTIR (F74G) + SMC13D-3xFLAG, SMC1-mClover-mAID HCT116 OsTIR (F74G) + SMC14E-3xFLAG were generated in house. FLAG-Halo-SMARCAL1 HCT116 OsTIR (F74G) were generated in house. HEK293T were purchased from ATCC (CRL-3216).
Authentication	None of the cell lines were authenticated in house for this manuscript.
Mycoplasma contamination	All cell lines used in this study are subject to our regular mycoplasma testing in house, and all of them consistently reported negative results during the course of the study.
Commonly misidentified lines<br>(See ICLAC register)	No commonly misidentified cell lines were used in this study.

Plants

Seed stocks	*Report on the source of all seed stocks or other plant material used. If applicable, state the seed stock centre and catalogue number. If plant specimens were collected from the field, describe the collection location, date and sampling procedures.*
Novel plant genotypes	*Describe the methods by which all novel plant genotypes were produced. This includes those generated by transgenic approaches, gene editing, chemical/radiation-based mutagenesis and hybridization. For transgenic lines, describe the transformation method, the number of independent lines analyzed and the generation upon which experiments were performed. For gene-edited lines, describe the editor used, the endogenous sequence targeted for editing, the targeting guide RNA sequence (if applicable) and how the editor was applied.*
Authentication	*Describe any authentication procedures for each seed stock used or novel genotype generated. Describe any experiments used to assess the effect of a mutation and, where applicable, how potential secondary effects (e.g. second site T-DNA insertions, mosiacism, off-target gene editing) were examined.*

Flow Cytometry

Plots

Confirm that:

- [x] The axis labels state the marker and fluorochrome used (e.g. CD4-FITC).
- [x] The axis scales are clearly visible. Include numbers along axes only for bottom left plot of group (a 'group' is an analysis of identical markers).
- [x] All plots are contour plots with outliers or pseudocolor plots.
- [x] A numerical value for number of cells or percentage (with statistics) is provided.

Methodology

Sample preparation	Cell lines were labeled with 25 µM EdU for 30 min, harvested by standard trypsinization, washed with PBS once and fixed for 12 min with 4% formaldehyde in PBS. Cells were permeabilized with 0.25% Triton-X in PBS, washed twice with PBS, and EdU was labeled by incubating in click reaction (0.1 M Tris pH 8.5, 0.1 M sodium-L-ascorbate, 2 mM CuSO4 and 0.1 mM Alexa Fluor-linked azide (Thermo Fisher)) for 1 h at RT. Cells were washed twice with PBS and DNA was stained with 1 µg/mL DAPI in PBS for 1 h. Samples were run on Attune NxT Flow Cytometer (Thermo Fisher) and analyzed using FlowJo software V.10.0.8 (FlowJo, LLC). At least 10000 cells were measured per condition. For gating strategy, see Supplementary Fig. 2.
Instrument	Samples were run on Attune NxT Flow Cytometer (Thermo Fisher)
Software	Samples were analyzed using FlowJo software V.10.0.8 (FlowJo, LLC).
Cell population abundance	Single cell population was around 40% of the total
Gating strategy	Cells were gated based on size and complexity (FSC-A vs. SSC-A). Cell doublets and aggregates were subsequently excluded using DAPI-H vs. DAPI-A.

- [x] Tick this box to confirm that a figure exemplifying the gating strategy is provided in the Supplementary Information.
