# Supplementary material for: Cohesin reshapes replication fork contacts to aid fork slowing and reversal
Source: Nature. 2026 Sep 16;658(8137):1057–65. doi: 10.1038/s41586-026-11034-0 (PMC13686946; doi:10.1038/s41586-026-11034-0)
Supplement: Supplementary file 3 [file 41586_2026_11034_MOESM3_ESM.pdf]

nature portfolio

Peer Review File

Cohesin reshapes replication fork contacts to aid fork slowing and reversal

Corresponding Author: Professor Massimo Lopes

Any redactions in this file are there to maintain patient confidentiality, the confidentiality of unpublished data, or to remove third-party material.

This file contains all reviewer reports in order by version, followed by all author rebuttals in order by version.

Version 0:

Reviewer comments:

Referee #1

(Remarks to the Author)

In their manuscript, González-Acosta and colleagues address the important question of how human cells stabilize stressed replication forks within the context of complex 3D genome architecture. They reveal a cohesin-mediated loop extrusion mechanism that organizes stalled forks into defined spatial domains, thereby safeguarding genome integrity under replication stress.

More specifically, the authors first employed quantitative iPOND-MS to demonstrate cohesin enrichment at HU-arrested replication forks, validated by SIRF imaging. NIPBL depletion reduced this cohesin accumulation and weakened chromatin loops (TADs) without disrupting cell cycle progression, distinguishing stress-specific cohesin loading from canonical cohesion establishment. Depletion of Sororin and WAPL had no measurable effect, confirming that fork-associated cohesin operates independently of cohesion regulators. SMC1 separation-of-function mutants (SMC1-3D and SMC1-4E) confirmed that loop extrusion and not cohesion drives cohesin function at stalled forks.
Using Repli-C, a Micro-C variant targeting nascent DNA, they observed fountain-like 3D interaction profiles around replicating regions, reflecting sister fork coupling and forming independently of cohesin. Cohesin-mediated loop extrusion was, however, essential for inter-replicon contacts that intensify during stress, enabling higher-order coordination between adjacent forks.
Functionally, RAD21 depletion blocked fork reversal (visualized by electron microscopy) and unleashed PRIMPOL-dependent repriming. Cohesin also supported fork resection in BRCA2-deficient cells, consistent with its role in fork reversal.

Collectively, these results position cohesin as a central player in replication-associated 3D chromatin organization and a key regulator of fork plasticity via NIPBL-dependent loading and loop extrusion, independently of sister chromatid cohesion. The manuscript is well written with high-quality data, though mechanistic aspects require strengthening, especially regarding the impact of loop extrusion on replication fork reversal and on the inhibition of PRIMPOL-mediated repriming of DNA synthesis.

Specific issues:

1. The authors used iPOND-MS to analyze replication fork–associated factors under two conditions: 100 µM HU for 1 h and 2 mM HU for 2 h. The enrichment of cohesin components at replication sites in both cases is particularly interesting. While treatment with 2 mM HU causes a severe (70–80%) depletion of dNTP pools and leads to fork stalling, 100 µM HU results in only a modest (10–20%) reduction. Nevertheless, forks slow down under the milder condition through an active mechanism involving fork reversal. The recruitment of cohesin and related factors under both conditions is thus compelling, but additional context on these distinct modes of fork impediment would help a broader audience appreciate the difference. The authors cite a study indicating that fork slowing in 50 µM HU is mediated by oxidative stress (ref. 35); they should also reference an earlier report describing fork slowdown at 100 µM HU (PMID: 24347643).

2. Page 4, the sentence "Since cohesin is recruited to stalled replication forks via NIPBL-dependent loop extrusion…"

requires additional explanation for non-specialist readers. While the authors demonstrate that NIPBL is required to recruit cohesin to stressed forks and to mediate cohesin-dependent fork tethering, the mechanistic link is not immediately obvious. The authors should either cite ref. #2 here or briefly note that human cohesin extrudes DNA loops only when loaded by NIPBL/MAU2 (e.g., Davidson et al., PMID: 31753851).

3. The Repli-C and Micro-C data are highly convincing and well presented. However, the authors should cite earlier yeast studies reporting similar cohesin accumulation at HU-stalled forks. For example, Jeppsson et al. recently showed that in budding yeast, cohesin accumulates at stalled forks in a Scc2/NIPBL-dependent manner, acting as loop extrusion barriers (PMID: 35687682). Although their conclusions differ from those here, this precedent warrants discussion. Similarly, the Jost lab proposed replication-dependent, cohesin-independent "fountain" formation (PMID: 41430292), which should also be addressed and cited.

4. The authors use nascent DNA resection in BRCA2-defective cells as an indirect readout for fork reversal proficiency. Since fork reversal also mediates slowdown in control cells treated with 100 µM HU, they should directly assess whether cohesin/NIPBL depletion prevents this slowdown.

5. The data convincingly place NIPBL/cohesin upstream of RAD51 in promoting fork reversal while restraining PRIMPOL repriming in a RECQ1-independent manner. However, the underlying mechanism (how loop extrusion biases reversal over repriming through fork tethering) remains underexplored. The authors should speculate on this (e.g., topological constraints, sequestration of CMG helicase…).

6. NIPBL depletion sensitizes cells to replication stress via impaired fork reversal and unchecked PRIMPOL activity, leading to fitness loss. How does this phenotype compare to SMARCAL1-defective cells under RS? Moreover, does PRIMPOL knockdown rescue HU sensitivity in NIPBL-defective cells? These experiments would strengthen the model by formally demonstrating that cohesin-mediated loop extrusion primarily favors fork reversal to restrain PRIMPOL-dependent repriming.

Referee #2

(Remarks to the Author)
This article provides an impressive amount of high-quality, well-controlled experiments to investigate the role of cohesin in the replication stress response. The authors demonstrate that cohesin accumulates at stressed replication forks in a manner dependent on NIPBL, a component of the cohesin loader that is also required for loop extrusion by cohesin. They show that previously described "fountain" structures, that presumably reflect a coupling of newly replicated DNA stretches located at symmetrical positions around replication initiation zones, are reinforced by cohesin depletion, suggesting that cohesin may normally limit the proximity of sister forks. Fork stalling by hydroxyurea (HU) results in restricted intra-replicon interactions but also in increased interactions between newly replicated DNA stretches from neighboring replicons, the latter depending on loop extrusion by cohesin. Furthermore, loop extrusion by cohesin is shown to mediate the slowdown of stressed forks by preventing PRIMPOL action and promoting replication fork reversal. Thus, loop extrusion by cohesin is shown to play a crucial role in the cellular response to replication stress.

My criticisms are only relatively minor

1. In the first paragraph of the main text, it is stated that DNA replication initiation takes place "at highly defined initiation zones". Recent evidence by single-molecule analysis (PMID: 41577668; PMID: 40346587) instead suggests that initiation zones previously identified in cell populations are only enriched 2-fold in initiation events and that 80% of initiation events actually occur in a dispersed manner between these zones. This statement should therefore be rectified. This has implications for the interpretation of interactions between so-called "sister" forks which may in fact originate from different replication initiation sites. I therefore encourage the authors to revise some oversimplified interpretations of their data. Repli-C do not reveal interactions between sister forks but only interactions between newly replicated DNA stretches. Finding the interacting stretches at symmetrical positions around initiation zones does not imply that these stretches were synthesised by sister forks initiated at the same site.

2. In the second paragraph, when the authors explain that NIPBL-MAU2 is required for loop extrusion, they should explain that NIPBL-MAU2 is also required to load cohesin on chromatin.

3. Given that NIPBL is required for cohesin loading, it is puzzing that NIPBL depletion reduced but did not abolish the SMC1 SIRF signal in the presence or absence of HU (Fig. 1f). Could this residual loading be due to incomplete depletion of NIPBL (Fig. 1e, depletion appears to be 95% but not 100%) ? Furthermore, in Fig 1f, the SMC1 SIRF signal seems higher in the presence than in the absence of HU even in NIPBL-depleted cells, but the authors do not report whether this is statistically significant or not. If this difference is still significant, this could again suggest a residual activity of NIPBL-MAU2 due to incomplete depletion of NIPBL.

4. Extended data Fig 1h includes a control from MCM2_ cells but I could not find a description of these cells or a reference in the text or in the Methods. Please explain how these cells were obtained or constructed. I would have expected that cells lacking MCM2 are not viable. Please explain.

5. ED Fig 3i, the authors show that CTCF depletion decreased cohesin enrichment near forks in the absence of HU (although no statistical testing is reported for the comparison of these two points) but not in the presence of HU. They

conclude that the effect of NIPBL depletion on cohesin recruitment is not a secondary effect of TAD disorganisation and that loop extrusion is required for cohesin recruitment at stalled forks. Can they exclude that the cohesin loading activity of NIPBL, rather than its loop extrusion activity, is the critical factor required for cohesin association with stalled forks?

6. An enhancement of the fountain signal in the absence of cohesin, as shown in Fig 2c, has been previously reported in budding yeast by D'Asaro et al (PMID: 41430292). This reference should be quoted and discussed.

7. What is the meaning of "EV" in ED Fig5?

8. The scheme shown in Figure 4g (with cohesin loop extrusion) lacks explanations and I did not immediately understand it. Please specify what the double arrows and red crosses represent, explain that yellow loops are shortened due to fork reversal, put a specific mark on the novel, stress-induced interaction between the two adjacent replicons (e.g. by changing the color of the newly-interacting MCMs) and write a few explanatory sentences in the Figure legend.

Referee #3

(Remarks to the Author)
The manuscript 'Cohesin-mediated replication fork tethering enables fork slowing and reversal' by González-Acosta reports that in response to replication stress, cohesin accumulates at stalled replication forks, strengthens inter-replicon contacts and promotes fork slowing and reversal. The authors propose that these phenomena are dependent on cohesin's loop extruding activity but not on cohesin's ability to mediate cohesion between replicated sister chromatids. Unrestrained DNA replication observed in response to replication stress in the absence of cohesin was shown to be dependent on the DNA primase and polymerase PRIMPOL, leading to the hypothesis that loop extrusion by cohesin shields stalled replication forks from PRIMPOL, allowing for fork reversal to take place and genome stability to be maintained.

This is an interesting hypothesis that would reveal a previously unknown function for cohesin-mediated loop extrusion and its role in cellular responses to replication stress. The described experiments are state of the art, the data of high quality, the manuscript is well written and the number of experimental approaches that have been taken to test the authors' hypothesis is impressive. However, there are several important aspects that require clarification.

1. The authors conclude that impaired cohesin loading cannot account for the defect in cohesin accumulation at stalled forks in the presence of HU after NIPBL depletion. This is based on the finding that depletion of NIPBL leads to a large reduction in EdU-SMC1 SIRF signal but only a minor reduction in the level of chromatin bound SMC1 (to 72.8 – 91.3 %).

To increase confidence in this conclusion, it would be important to repeat this chromatin-binding and stalled fork recruitment comparison ± NIPBL depletion when performing SIRF on a different core cohesin subunit, e.g. RAD21. This is especially important since RAD21 SIRF was performed in all the experiments which did not show a significant defect in SIRF recruitment following HU treatment ± auxin (i.e. the Sororin, WAPL and CTCF degron experiments), while SMC1 SIRF was not performed in these latter experiments. Such an experiment could also strengthen the authors' conclusion that cohesion-related functions of cohesin are not important for its accumulation at stalled replication forks.

2. At higher doses and upon prolonged incubation times HU can induce replication fork collapse and lead to formation of DNA double strand breaks (DSBs). Can the authors exclude that HU induced the formation of DSBs in their experiments? This is an important point since DSBs have been reported to recruit cohesin and to function as sites at which one-sided cohesin mediated loop extrusion is initiated (Arnould et al., 2021; Teleoni et al., 2025). In the reported experiments, HU treatment could therefore also have altered cohesin's behaviour through DSB induction.

Related to this, why were different regimens of HU treatment used in different experiments (2 mM, 100 µM, 50 µM, 4 mM HU). It would be helpful to explain the rationale for this.

2. The authors performed Sororin, CTCF and Wapl depletions and showed that these perturbations did not affect the accumulation of cohesin at stalled replication forks. Although these depletions appear to be efficient (Extended Data Fig. 3) there is no data in the manuscript to show that the depletions were sufficient to induce the previously published phenotypes of these depletion conditions (i.e. loss of sister chromatid cohesin upon Sororin depletion, loss of TADs and loops upon CTCF depletion and increase in interaction length and number and size of loops upon Wapl depletion). If the specific cell lines that have been used in this study have been previously published, these papers should be cited. If not, the above analyses should be performed and the results included.

3. While the effect of HU on origin fountain structures as detected by Repli-C is detectable in aggregate genome wide analyses, the effect on the individual fountains shown in Fig. 2c is small and a complete comparison between Micro-C and Repli-C for the same genomic region in all conditions is missing. Could the authors show both Micro-C and Repli-C in the presence and absence of HU and SMC1 depletion for the same region, e.g. plot the same region as shown in Fig. 2b but for all conditions and not only wildtype?

4. The authors have extensively studied the contribution of loop extruding cohesin versus cohesive cohesin on the accumulation of cohesin at stalled replication forks using the SIRF assay (NIPBL depletion, Sororin depletion, SMC1-3D expression, SMC1-4E expression). The authors have also identified replication origin fountains and inter-replisome contacts using Repli-C and have shown that these structures are affected to a greater or lesser extent following SMC1 depletion or

SMC1-4E expression. Can the authors test whether Sororin depletion or SMC1-3D expression affects these structures as well? This would provide increased confidence that these structures are influenced by loop extrusion and not cohesive cohesin.

5. To support the hypothesis that the loop extrusion and not the cohesion function of cohesin contributes to replication fork reversal and slowing the authors used cohesin containing SMC1-3D and SMC1-4E as mutants that are selectively defective in cohesion and loop extrusion, respectively. However, the SMC1-3D mutant is not only defective in cohesion but has also been reported to have some defects in loop extrusion (Nagasaka et al., 2023). This raises the question whether the loop extrusion activities of these mutants really correlate with their accumulation at stalled replication forks and the formation of fountains and inter-replisome contacts. To test how good these correlations are it would be important to analyse the phenotypes of these mutants also in Micro-C experiments and to compare these data to the effects of these mutants at stalled replication forks.

6. The authors' proposal that loop extrusion mediated fork slowdown upon HU treatment protects the forks from restart by PRIMPOL is based on rather complicated combinatorial depletions followed by DNA fibre analysis only and thus remains speculative. It would be useful to strengthen these conclusions experimentally. For example, the authors conclude that cohesin shields DNA from PRIMPOL activity, but they do not show PRIMPOL accumulation when cohesin is depleted. iPOND experiments ± cohesin should be performed to support the claim directly.

Minor points

1. 'Length' spelt incorrectly in y axis Fig. 2d.

2. Page 4, 'Moreover, SMC1 depletion drastically increased the fountain length, while HU consistently reduced it independently of cohesin'. The language should be toned down here. From the graphs presented in Fig. 2d, SMC1 depletion led to less than a ~ 1.5x increase in fountain length.

3. SMC1 spelt incorrectly in Extended Data Fig. 6a upper right most panel.

Version 1:

Reviewer comments:

Referee #1

(Remarks to the Author)
In my view, the authors have done an excellent job revising the manuscript and have addressed most, if not all, of the concerns raised by the three reviewers. They have added key experiments that strengthen their conclusions and made a substantial effort to improve the accessibility of the work for non-specialist readers, both in the main text and through new or improved graphical models. Regarding the issue of accessibility for non-specialized readers, I would be somewhat cautious about reviewer #2's point #1. While they are entirely right to emphasize that recent single-molecule studies suggest that fewer than 20% of initiation events occur within initiation zones identified by population-based approaches (Pu-seq, SNS-seq, OK-seq…), being too cautious in using Pu-seq data to interpret Repli-C results could be potentially confusing to readers. Since Repli-C is also a population-based assay, comparing the positions identified by these methods is fully appropriate.

Referee #2

(Remarks to the Author)
I am satisfied with the authors'answers to my comments.

Referee #3

(Remarks to the Author)
The authors have carefully revised their manuscript, in many cases by performing additional experiments and analyses. As a result, the authors have addressed and clearly answered almost all my comments and questions.

A single exception was my suggestion to perform Micro-C experiments with cells expressing the mutated cohesin subunits SMC1-4E and SMC1-3D to test directly if the effects of these mutations on fork restart really correlate with their effects on genome architecture. The purpose of these experiments would have been to obtain additional evidence that it is cohesin's loop extrusion and not its sister chromatid cohesion function in controlling replication fork slowdown upon HU treatement. However, the authors provide other lines of evidence in support of a loop extrusion not cohesion role of cohesin in this process. I therefore agree with the authors that these Micro-C experiments are not absolutely essential.

The revision has further strengthened this manuscript, which is impressive with respect to the amount and quality of data, resulting in a study that is exceptionally rigorous and advances both the genome architecture and replication stress fields conceptually in important ways. I would therefore now fully support publication of this interesting paper in Nature.

Open Access This Peer Review File is licensed under a Creative Commons Attribution 4.0 International License, which permits use, sharing, adaptation, distribution and reproduction in any medium or format, as long as you give appropriate credit to the original author(s) and the source, provide a link to the Creative Commons license, and indicate if changes were made.
In cases where reviewers are anonymous, credit should be given to 'Anonymous Referee' and the source.
The images or other third party material in this Peer Review File are included in the article's Creative Commons license, unless indicated otherwise in a credit line to the material. If material is not included in the article's Creative Commons license and your intended use is not permitted by statutory regulation or exceeds the permitted use, you will need to obtain permission directly from the copyright holder.
To view a copy of this license, visit https://creativecommons.org/licenses/by/4.0/

Referees' comments:

- We thank all three reviewers for the in-depth analysis of our manuscript and their great support towards the data presented here. We believe their comments have been instrumental to strengthen the conclusions presented in the study.

For those comments where new data has been included, we provide the modified text lines here. The figure legends of those new panels have been also updated and can be seen in the manuscript or supplementary information documents.

Referee #1 (Remarks to the Author):

In their manuscript, González-Acosta and colleagues address the important question of how human cells stabilize stressed replication forks within the context of complex 3D genome architecture. They reveal a cohesin-mediated loop extrusion mechanism that organizes stalled forks into defined spatial domains, thereby safeguarding genome integrity under replication stress.

More specifically, the authors first employed quantitative iPOND-MS to demonstrate cohesin enrichment at HU-arrested replication forks, validated by SIRF imaging. NIPBL depletion reduced this cohesin accumulation and weakened chromatin loops (TADs) without disrupting cell cycle progression, distinguishing stress-specific cohesin loading from canonical cohesion establishment. Depletion of Sororin and WAPL had no measurable effect, confirming that fork-associated cohesin operates independently of cohesion regulators. SMC1 separation-of-function mutants (SMC1-3D and SMC1-4E) confirmed that loop extrusion and not cohesion drives cohesin function at stalled forks.

Using Repli-C, a Micro-C variant targeting nascent DNA, they observed fountain-like 3D interaction profiles around replicating regions, reflecting sister fork coupling and forming independently of cohesin. Cohesin-mediated loop extrusion was, however, essential for inter-replicon contacts that intensify during stress, enabling higher-order coordination between adjacent forks.

Functionally, RAD21 depletion blocked fork reversal (visualized by electron microscopy) and unleashed PRIMPOL-dependent repriming. Cohesin also supported fork resection in BRCA2-deficient cells, consistent with its role in fork reversal.

Collectively, these results position cohesin as a central player in replication-associated 3D chromatin organization and a key regulator of fork plasticity via NIPBL-dependent loading and loop extrusion, independently of sister chromatid cohesion. The manuscript is well written with high-quality data, though mechanistic aspects require strengthening, especially regarding the impact of loop extrusion on replication fork reversal and on the inhibition of PRIMPOL-mediated repriming of DNA synthesis.

- We thank the reviewer for the support and the detailed analysis of our manuscript, which has helped us putting our results in context for non-experts and strengthening the conclusions of our data. In an effort to make the manuscript easier to understand for non-experts, we have included new drawings – explained within the respective figure legends – within the new figures 1a, 3a, 3f and 4e.

Specific issues:

1. The authors used iPOND-MS to analyze replication fork–associated factors under two conditions: 100 μM HU for 1 h and 2 mM HU for 2 h. The enrichment of cohesin components at replication sites in both cases is particularly interesting. While treatment with 2 mM HU causes a severe (70–80%) depletion of dNTP pools and leads to fork stalling, 100 μM HU results in only a modest (10–20%) reduction. Nevertheless, forks slow down under the milder condition through an active mechanism involving fork reversal. The recruitment of cohesin and related factors under both conditions is thus compelling, but additional context on these distinct modes of fork impediment would help a broader audience appreciate the difference. The authors cite a study indicating that fork slowing in 50 μM HU is mediated by oxidative stress (ref. 35); they should also reference an earlier report describing fork slowdown at 100 μM HU (PMID: 24347643).

- As suggested, we have updated the first paragraph of the *Results* section (page 3), including for non-experts a more detailed explanation on the effects of different HU treatments and a citation of the suggested report (new ref. 40):

"…we characterized the proteome landscape of replication forks when cells experience two different treatments with the ribonucleotide reductase (RNR) inhibitor Hydroxyurea (HU): 2 hours at 2 mM, which stalls all ongoing replication forks by severely depleting the cellular nucleotide pool[39]; and 1 hour at 100 μM, which induces fork

slowdown[40] through mild reduction of cellular nucleotides and by promoting TIMELESS dissociation at forks via reactive oxygen species (ROS) and disruption of oligomerized PRDX2[41]."

2. Page 4, the sentence "Since cohesin is recruited to stalled replication forks via NIPBL-dependent loop extrusion…" requires additional explanation for non-specialist readers. While the authors demonstrate that NIPBL is required to recruit cohesin to stressed forks and to mediate cohesin-dependent fork tethering, the mechanistic link is not immediately obvious. The authors should either cite ref. #2 here or briefly note that human cohesin extrudes DNA loops only when loaded by NIPBL/MAU2 (e.g., Davidson et al., PMID: 31753851).

- We thank the reviewer for pointing this out. We have included citations of the suggested references (new ref. 17 and 18) while introducing cohesin loop-extrusion activity in page 2:

"The cohesin complex, composed by SMC1, SMC3, RAD21 and either STAG1 or STAG2, organizes the genome into TADs and chromatin loops thanks to its ability to extrude DNA, bringing together distant DNA elements[17–19]."

We also included a citation of ref. 2 in the sentence indicated in the comment that now reads as follows:

"If cohesin is recruited to stalled replication forks via NIPBL-dependent loop extrusion[2], we hypothesized that it may be required to reorganize chromatin contacts at replicating regions in response to RS."

3. The Repli-C and Micro-C data are highly convincing and well presented. However, the authors should cite earlier yeast studies reporting similar cohesin accumulation at HU-stalled forks. For example, Jeppsson et al. recently showed that in budding yeast, cohesin accumulates at stalled forks in a Scc2/NIPBL-dependent manner, acting as loop extrusion barriers (PMID: 35687682). Although their conclusions differ from those here, this precedent warrants discussion. Similarly, the Jost lab proposed replication-dependent, cohesin-independent "fountain" formation (PMID: 41430292), which should also be addressed and cited.

- We thank the reviewer for pointing out those two studies. Although cohesin regulation, genome organization and the replication stress response differ considerably in yeast and humans, we agree that those studies should be cited in the context of our findings. Two sentences have been included in the text where those studies are referenced:

"This is in line with a recent report showing cohesin-independent fountain structures at early firing origins in Hi-C maps in yeast[50]." (new reference 50, PMID: 41430292) in the Results section (page 5).

"In yeast, stalled replication forks were shown to act as barriers for cohesin-mediated loop extrusion in Hi-C experiments[52]." (new reference 52, PMID: 35687682) in the Results section (page 5).

4. The authors use nascent DNA resection in BRCA2-defective cells as an indirect readout for fork reversal proficiency. Since fork reversal also mediates slowdown in control cells treated with 100 μM HU, they should directly assess whether cohesin/NIPBL depletion prevents this slowdown.

- As shown in figure 3b-d, forks do not slowdown upon treatment with 50 μM HU in Rad21-, NIPBL- or SMC1-depleted cells. In this condition, nucleotide levels are only slightly reduced (10%), and the slowdown is primarily driven by ROS-mediated replisome re-arrangement (PMID: 29123070) and active fork reversal (PMID: 32442397). However, nucleotide depletion is expectedly more pronounced upon 100μM HU, which may per se contribute to the more marked slowdown expected in these conditions. To directly address the reviewer's question, we have performed DNA fibers at 100 μM HU in the presence or absence of Rad21 and NIPBL (shown below, for reviewers only). We do observe a higher reduction in fork speed compared to 50μM (figures 3b and c) in line with higher depletion of nucleotides. However, even at this higher HU concentration, forks still undergo unrestrained fork progression, albeit incomplete (i.e. not reaching a median ratio of 1), due to lower residual nucleotide levels.

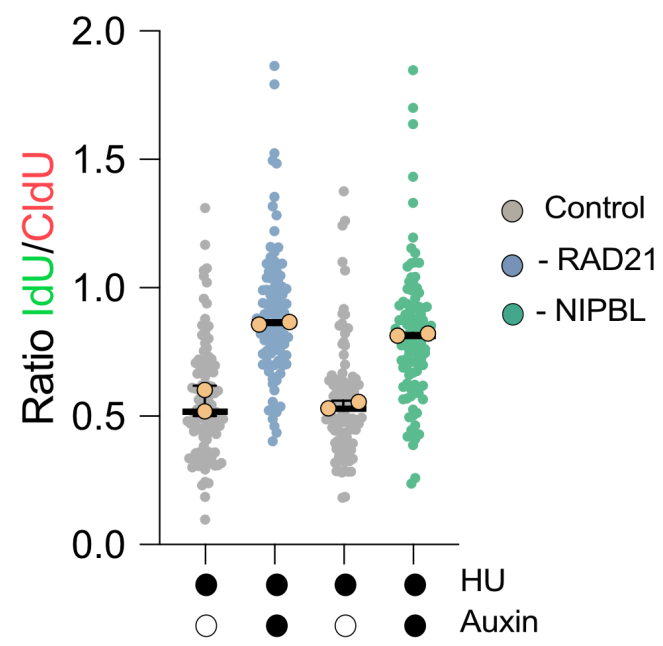

5. The data convincingly place NIPBL/cohesin upstream of RAD51 in promoting fork reversal while restraining PRIMPOL repriming in a RECQ1-independent manner. However, the underlying mechanism (how loop extrusion biases reversal over repriming through fork tethering) remains underexplored. The authors should speculate on this (e.g., topological constraints, sequestration of CMG helicase…).

- We agree with the reviewer that this aspect needed further speculation, to boost interpretation and discussion of our key findings. Hence, we have modified as follows the last paragraph of the Discussion, including different scenarios that we envision as possible mechanisms by which cohesin might regulate fork progression.

"Taken all together, we have observed that cohesin limits sister fork coupling as forks progress away from the origins, restraining fountain extension within replicons. When S-phase is challenged by fork stalling agents, cohesin accumulates in proximity to stalled forks through loop extrusion. Mechanistically, we propose that cohesin interacts with the CMG-helicases belonging to adjacent replicons through the MCM complex[21]. This might restrain the uncoupling of helicase and polymerase activities and limit the exposure of large ssDNA stretches, which have been shown to promote PRIMPOL repriming *in vitro*[70]. Supercoiling generated by cohesin-mediated loop extrusion may further restrict helicase progression during uncoupling, which might be favored in the absence of loop extrusion[53]. Preventing PRIMPOL action may also stabilize short ssDNA stretches arising during uncoupled DNA synthesis, thereby facilitating the more complex biochemical reactions and DNA rearrangements required to drive fork reversal and ultimately maintain genome stability (**Fig. 4h**). These results identify loop-extruding cohesin as a critical mediator of the cellular response to replication stress and to cancer chemotherapeutic treatments**.**" (new ref. 69, PMID: 28396594) at the end of the Results section (page 9).

6. NIPBL depletion sensitizes cells to replication stress via impaired fork reversal and unchecked PRIMPOL activity, leading to fitness loss. How does this phenotype compare to SMARCAL1-defective cells under RS? Moreover, does PRIMPOL knockdown rescue HU sensitivity in NIPBL-defective cells? These experiments would strengthen the model by formally demonstrating that cohesin-mediated loop extrusion primarily favors fork reversal to restrain PRIMPOL-dependent repriming.

- We thank the reviewer for raising this important point. SMARCAL1-defective cells were previously reported to be sensitive to different genotoxic treatments (PMID: 19793862). To recapitulate this result, we generated a cell line with endogenous SMARCAL1 fused to a FLAG-Halo tag. Upon treatment with Halo-PROTAC3 (HP3), SMARCAL1 can be depleted to around 5% of its endogenous levels in 24h. We conducted DNA fiber assays in cells with and without SMARCAL1 exposed to mild HU treatment (50 μM) during IdU labeling, assessing IdU/CldU ratio to evaluate fork progression. We confirmed that SMARCAL1-depleted cells displayed unrestrained fork progression upon this treatment. We also exposed cells to HP3 (or vehicle as control) and left them untreated or treated them with 90 nM MMC for 24h and then released, with 150 μM HU or 50 nM Aph for 9 days. As shown below, SMARCAL1-depleted cells displayed higher sensitivity to all these agents, particularly to MMC or Aph.

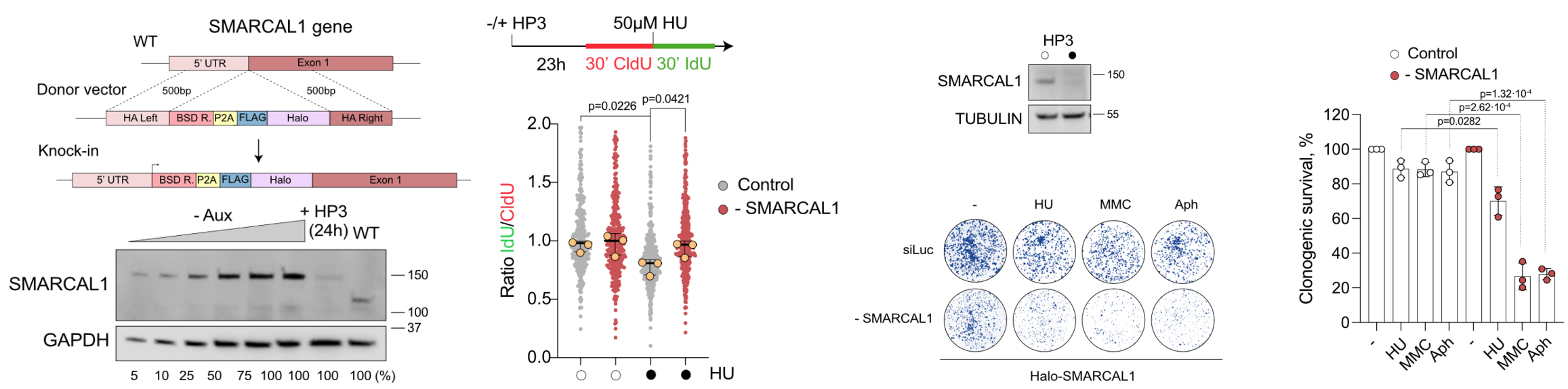

These results have now been included in Extended Data Figure 9 (j-n) and their description has been added to the *Results* section on page 8: "This sensitivity is comparable to that observed upon fork reversal-inactivation, induced by acute depletion of endogenously tagged Halo-SMARCAL1 after 24h-treatment with Halo-PROTAC3 (HP3), as validated in DNA fiber assays (**Extended Data Fig. 9j-n**).". The Methods section has been updated to describe the generation of the SMARCAL1 degron cell line.

Regarding HU sensitivity of NIPBL-depleted cells upon PrimPol depletion, we had indeed tested this possibility including PrimPol siRNA in our original sensitivity assays but decided not to include these results based on the mild effects observed upon PrimPol depletion, as described below.

While PrimPol can be efficiently depleted by siRNA, it is slowly re-expressed from day 4 to day 9, reaching ca. 60% of the initial levels by the end of the experiment. This partial depletion may justify the significant, but mild rescue of sensitivity that is observed. However, it is also likely that besides excessive PrimPol-mediated repriming, other deregulations contribute to long-term sensitivity to DNA damaging agents upon defective loop

extrusion. We consider this an open question for future studies, which does not affect the key conclusions of the current one, and for this reason we would prefer not to include these data. Nevertheless, we have refined our conclusions on these sensitivity data, acknowledging this complexity:

"Such sensitivity to genotoxic agents may partly reflect unleashed PRIMPOL action and toxic accumulation of ssDNA gaps; however, we note that cohesin mediated-loop extrusion also plays a role in the repair of DSBs, which are likely to arise upon the prolonged treatments used for these experiments[66–69]." (new reference 66-69, PMID: 33597753, PMID: 37024496, PMID: 41343653, PMID: 41343630) in the Results section (page 8).

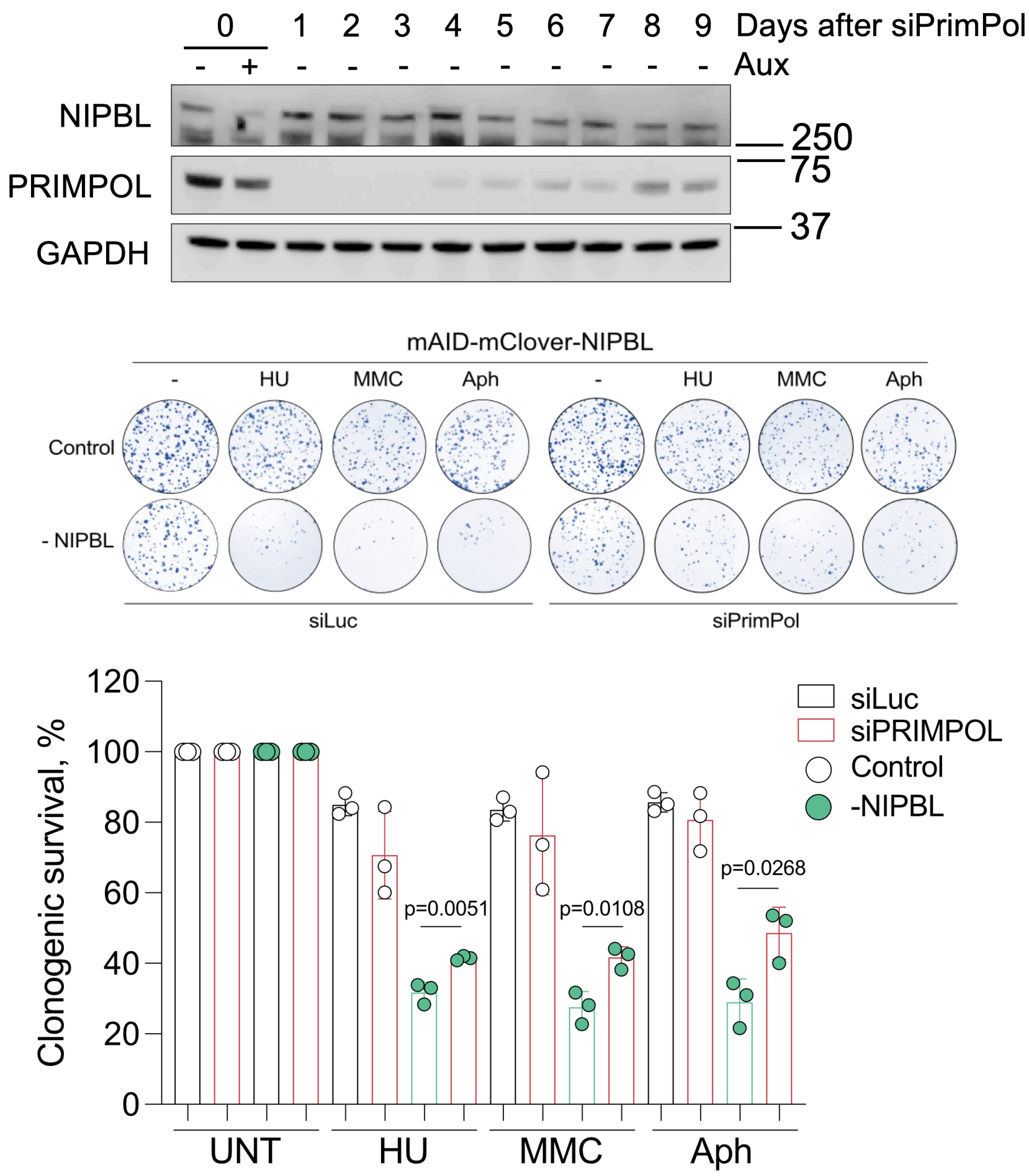

Referee #2 (Remarks to the Author):

This article provides an impressive amount of high-quality, well-controlled experiments to investigate the role of cohesin in the replication stress response. The authors demonstrate that cohesin accumulates at stressed replication forks in a manner dependent on NIPBL, a component of the cohesin loader that is also required for loop extrusion by cohesin. They show that previously described "fountain" structures, that presumably reflect a coupling of newly replicated DNA stretches located at symmetrical positions around replication initiation zones, are reinforced by cohesin depletion, suggesting that cohesin may normally limit the proximity of sister forks. Fork stalling by hydroxyurea (HU) results in restricted intra-replicon interactions but also in increased interactions between newly replicated DNA stretches from neighboring replicons, the latter depending on loop extrusion by cohesin. Furthermore, loop extrusion by cohesin is shown to mediate the slowdown of stressed forks by preventing PRIMPOL action and promoting replication fork reversal. Thus, loop extrusion by cohesin is shown to play a crucial role in the cellular response to replication stress.

- We appreciate the reviewer's positive feedback and thorough review, which helped considering new interpretations of our data and strengthening our conclusions.

My criticisms are only relatively minor

1. In the first paragraph of the main text, it is stated that DNA replication initiation takes place "at highly defined initiation zones". Recent evidence by single-molecule analysis (PMID: 41577668; PMID: 40346587) instead suggests that initiation zones previously identified in cell populations are only enriched 2-fold in initiation events and that 80% of initiation events actually occur in a dispersed manner between these zones. This statement should therefore be rectified. This has implications for the interpretation of interactions between so-called "sister" forks which may in fact originate from different replication initiation sites. I therefore encourage the authors to revise some oversimplified interpretations of their data. Repli-C do not reveal interactions between sister forks but only interactions between newly replicated DNA stretches. Finding the interacting stretches at symmetrical positions around initiation zones does not imply that these stretches were synthesised by sister forks initiated at the same site.

- We thank the reviewer for pointing this out and for encouraging us to consider alternative interpretations of our data. We agree that our model might oversimplify complex processes such as coordinated DNA synthesis. We have now modified as follows the first paragraph of the main text (page 2) to acknowledge this complexity, citing the corresponding literature:

"Initiation of DNA replication takes place at DNA replication origins that spread along the genome[13,14] and are modestly enriched in initiation zones (IZ) defined by population studies[15]. Recent Hi-C analyses of DNA replication contacts revealed that newly replicated DNA stretches within the same IZ – possibly representing sister forks of the same replicon and/or converging forks from neighboring replicons – are physically coupled during DNA synthesis[5]." (new ref. 13, 14, PMID: 34157308, PMID: 40346587).

2. In the second paragraph, when the authors explain that NIPBL-MAU2 is required for loop extrusion, they should explain that NIPBL-MAU2 is also required to load cohesin on chromatin.

- We have now included this in the text (second paragraph of the main text, page 2):

"This function requires its association with the cohesin chromatin loader NIPBL-MAU2, which promotes ATP hydrolysis by the SMC1/3 head domains[2,20]."

3. Given that NIPBL is required for cohesin loading, it is puzzing that NIPBL depletion reduced but did not abolish the SMC1 SIRF signal in the presence or absence of HU (Fig. 1f). Could this residual loading be due to incomplete depletion of NIPBL (Fig. 1e, depletion appears to be 95% but not 100%)? Furthermore, in Fig 1f, the SMC1 SIRF signal seems higher in the presence than in the absence of HU even in NIPBL-depleted cells, but the authors do not report whether this is statistically significant or not. If this difference is still significant, this could again suggest a residual activity of NIPBL-MAU2 due to incomplete depletion of NIPBL.

- We agree with the reviewer that the residual levels of NIPBL might still promote a certain level of cohesin presence at replication forks. Moreover, cohesin reduction on chromatin is not highly affected by acute NIPBL depletion (Extended Data Figure 2g and i, the latter requested by Reviewer#3), which is in line with recently published reports cited in the text. The residual presence of cohesin at replication forks might be due to replication forks meeting cohesin rings on chromatin positioned ahead of the replication fork, which might contribute to cohesion and is not affected by NIPBL depletion. Statistical analysis across 3 replicates comparing UNT and HU-treated cells in cells depleted from NIPBL reveals no statistically significant difference, which

argues in favor of a strong impairment of NIPBL activity upon auxin treatment. We have also provided this comparison for the new Extended Data Figure 2h, a Rad21 SIRF experiment requested by Reviewer#3, with similar results. Nonetheless, we have updated the figure including this comparison (see below) and included a sentence in the text (Results section; page 4) to consider this possibility:

"Finally, NIPBL depletion abolished cohesin SMC1 and RAD21 accumulation at stalled forks upon 2 mM HU treatment (**Fig. 1g and h, and Extended Data Fig. 2h**) with a marginal, non-significant, increase of cohesin at stalled forks upon HU treatment in NIPBL-depleted cells, likely reflecting incomplete NIPBL depletion in our degron system. This effect cannot be explained by impaired cohesin loading in the absence of NIPBL, since cohesin levels on chromatin are ~75-90% of those in the presence of NIPBL (**Extended Data Fig. 2g and i**), consistent with previous reports[45,46].".

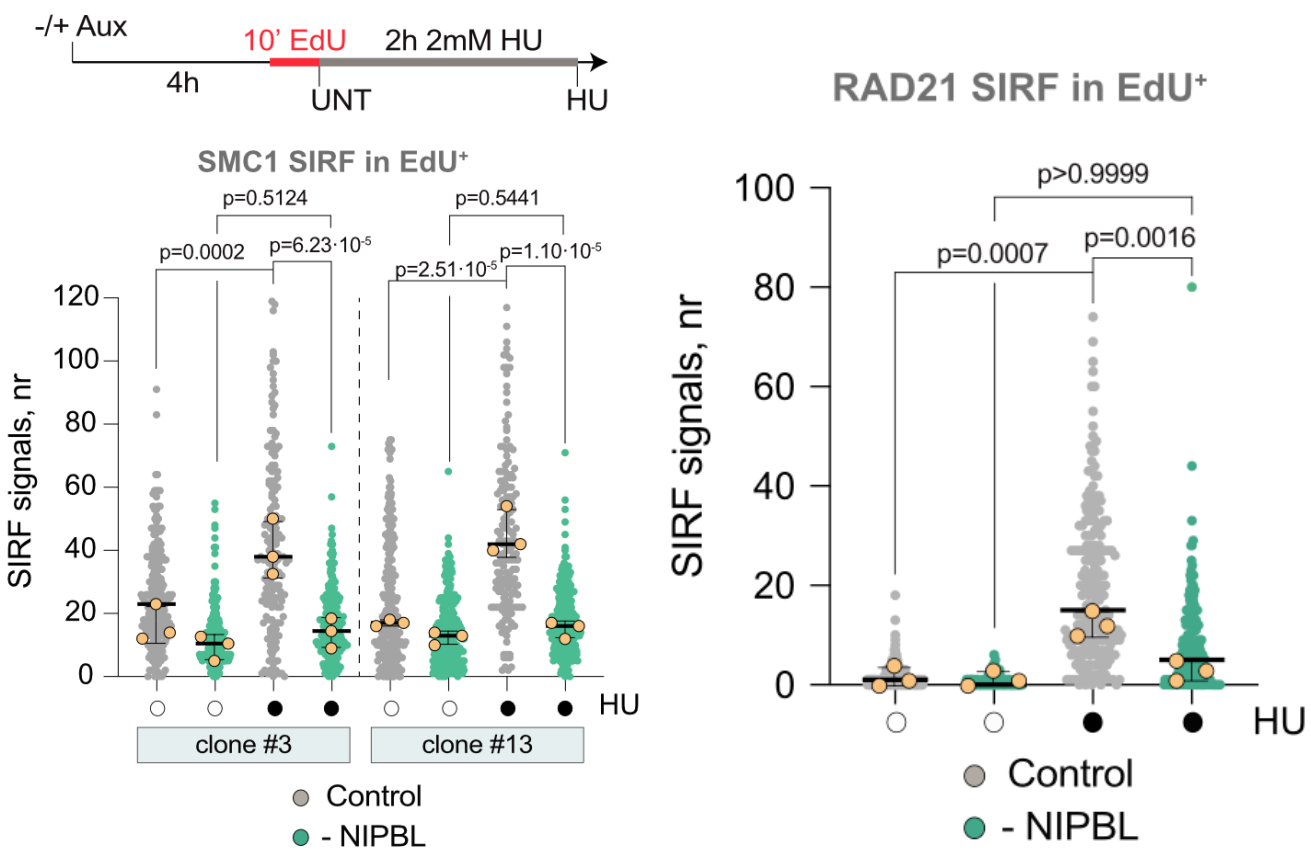

4. Extended data Fig 1h includes a control from MCM2_ cells but I could not find a description of these cells or a reference in the text or in the Methods. Please explain how these cells were obtained or constructed. I would have expected that cells lacking MCM2 are not viable. Please explain.

- We apologize for the confusion. The $MCM2^-$ cells refers to those cells in the population which are MCM2 negative (those that are in G2) and hence no MCM2 primary antibody should contribute to form PLA foci, showing that our assay is specific. A modification in the figure legend has been included to avoid any confusion:

"MCM2::SMC1 PLA upon 2 mM HU. MCM2- cells (G2 cells lacking MCM2 staining) from the UNT condition serve as negative a control." in the Extended Data Fig. 1 figure legend (page 9 of Supplementary information).

5. ED Fig 3i, the authors show that CTCF depletion decreased cohesin enrichment near forks in the absence of HU (although no statistical testing is reported for the comparison of these two points) but not in the presence of HU. They conclude that the effect of NIPBL depletion on cohesin recruitment is not a secondary effect of TAD disorganisation and that loop extrusion is required for cohesin recruitment at stalled forks. Can they exclude that the cohesin loading activity of NIPBL, rather than its loop extrusion activity, is the critical factor required for cohesin association with stalled forks?

- We thank the reviewer for pointing that out. Although CTCF depletion decreased cohesin presence at forks in the untreated (UNT) condition in two of the replicates, the third one does not show the same trend. We have added the statistical comparison between control and CTCF-depletion in the absence of HU, showing no significant difference (0.5853). Yet, we agree that there might be a trend in the direction indicated by the referee, and we have included a sentence in the Results section (page 4) discussing this possibility.

"…we analyzed cohesin recruitment at HU-stalled replication forks after CTCF elimination using a degron cell line previously generated[6,47]. As expected, CTCF depletion impaired TAD formation and weakened insulation scores[48]. We observed a non-significant trend towards decreased cohesin enrichment near forks in the absence of RS but no effect on cohesin recruitment to stalled forks (**Extended Data Fig. 3k-p**).".

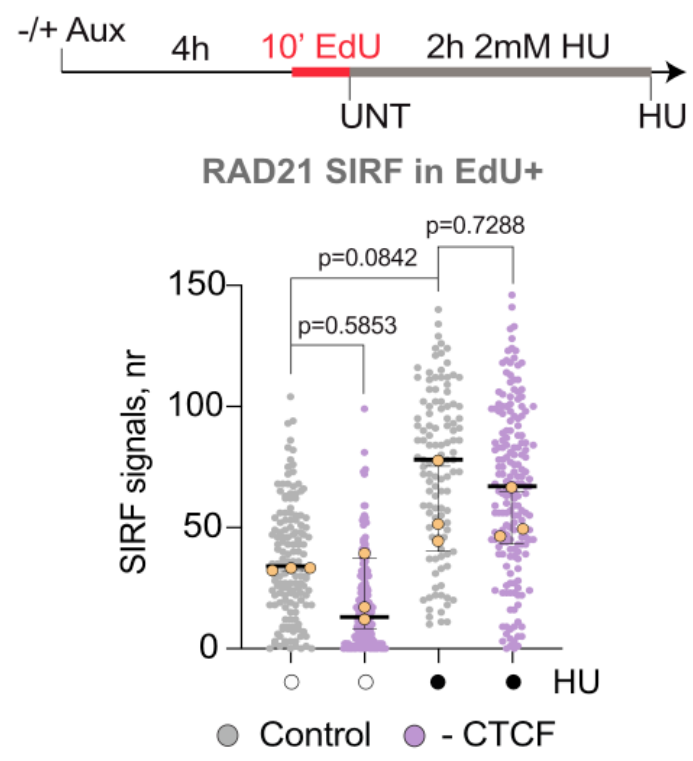

We cannot formally exclude that NIPBL uses its loading activity to accumulate cohesin at stalled forks, as – to our knowledge – no separation of function mutants are available to address this possibility. However, results from the SMC1-4E mutant indirectly indicate that those cohesin rings can be loaded on chromatin with a similar efficiency than the SMC1-WT but fail to accumulate at stalled replication forks. These results support loop extrusion as the key activity required for this function. Since in this early section of the manuscript we have not yet addressed separation of functions using the SMC1 mutants, we have now modified the text in the Results section (page 4) to mention both alternatives:

“This result rules out an indirect effect of disrupted TAD organization in the absence of NIPBL and rather indicates that cohesin loading and/or loop extrusion are essential for cohesin accumulation at stalled forks.”.

Moreover, we have modified the title of this Results section to emphasize the dependency on NIBPL (and not on the loop-extrusion activity) at this point of the manuscript:

“Cohesin accumulates at stressed replication forks in a NIPBL-dependent manner”

And we start the following part of results with the following sentence in page 4:

“If cohesin is recruited to stalled replication forks via NIPBL-dependent loop extrusion[2], we hypothesized that it may be required to reorganize chromatin contacts at replicating regions in response to RS.”

6. An enhancement of the fountain signal in the absence of cohesin, as shown in Fig 2c, has been previously reported in budding yeast by D'Asaro et al (PMID: 41430292). This reference should be quoted and discussed.

- We have now included a sentence in the text (Results section; page 5) referring to the suggested citation and putting it in the context of our studies.

“This suggests that these replicative structures are largely cohesin-independent; their extended detection in the absence of cohesin may reflect the disruption of alternative contacts that replicating regions establish with other regions in a cohesin-dependent manner. This is in line with a recent report in yeast, showing cohesin-independent fountain structures at early firing origins in Hi-C maps[50].” (new reference 50, PMID: 41430292).

7. What is the meaning of "EV" in ED Fig5?

- For consistency we have now changed EV (which means “empty vector”) to “-“ as in the other panels.

8. The scheme shown in Figure 4g (with cohesin loop extrusion) lacks explanations and I did not immediately understand it. Please specify what the double arrows and red crosses represent, explain that yellow loops are shortened due to fork reversal, put a specific mark on the novel, stress-induced interaction between the two adjacent replicons (e.g. by changing the color of the newly-interacting MCMs) and write a few explanatory sentences in the Figure legend.

- We thank the reviewer for the input on how to improve our model and make it more understandable. We now indicate that double arrows next to cohesin mean that the complex is moving while the double cross means that it is stopped when fork stalling is induced. We also indicate that fork stalling might promote disruption of coordinated synthesis and fountain shortening maintaining the detailed fork reversal drawing below. Finally, we have changed the color of the interacting MCM complexes (to dark blue) and included a few explanatory sentences in Figure 4 legend (page 18):

“Fountain-like structures derived from coordinated DNA synthesis – likely from sister forks – interact with mobile cohesin placed behind and ahead of ongoing replication forks. Upon fork stalling, coordinated DNA synthesis is disrupted and loop-extruding cohesin becomes arrested at both locations enhancing interactions inside the replicon and promoting inter-replicon contacts (dark blue CMG complexes), thereby limiting fork uncoupling and favoring fork reversal. In the absence of cohesin-mediated protective DNA contacts are not established, allowing the access of PRIMPOL primase, and promoting unrestrained fork progression.”.

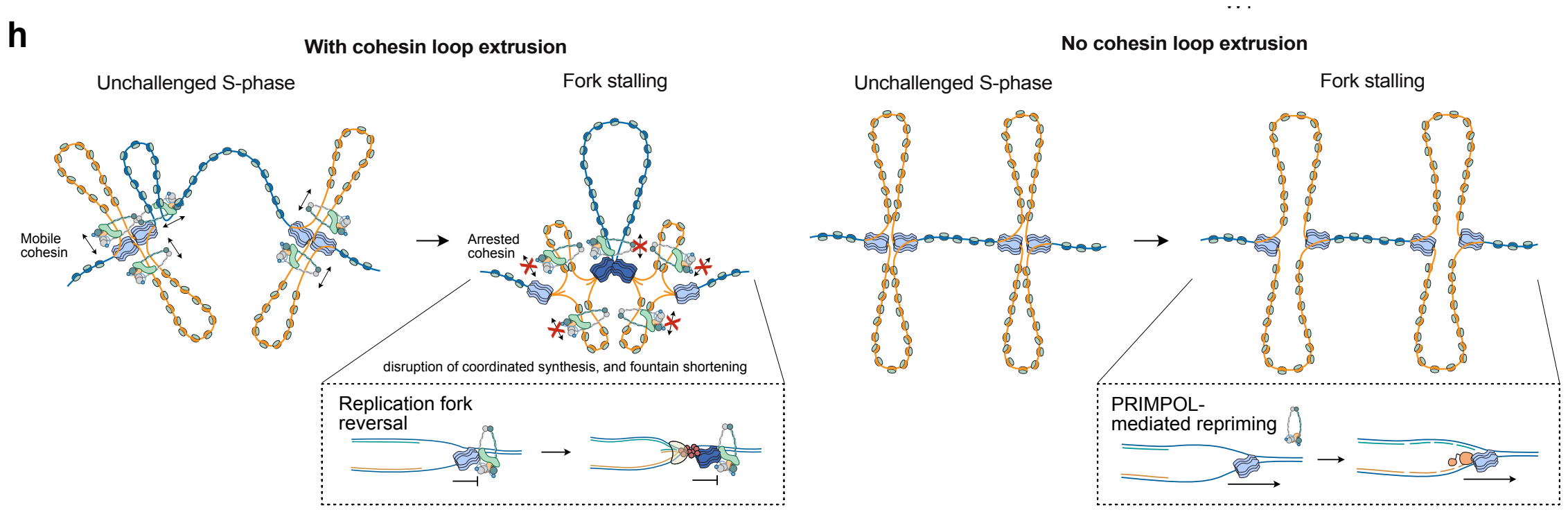

Referee #3 (Remarks to the Author):

The manuscript ‘Cohesin-mediated replication fork tethering enables fork slowing and reversal’ by González-Acosta reports that in response to replication stress, cohesin accumulates at stalled replication forks, strengthens inter-replicon contacts and promotes fork slowing and reversal. The authors propose that these phenomena are dependent on cohesin’s loop extruding activity but not on cohesin’s ability to mediate cohesion between replicated sister chromatids. Unrestrained DNA replication observed in response to replication stress in the absence of cohesin was shown to be dependent on the DNA primase and polymerase PRIMPOL, leading to the hypothesis that loop extrusion by cohesin shields stalled replication forks from PRIMPOL, allowing for fork reversal to take place and genome stability to be maintained.

This is an interesting hypothesis that would reveal a previously unknown function for cohesin-mediated loop extrusion and its role in cellular responses to replication stress. The described experiments are state of the art, the data of high quality, the manuscript is well written and the number of experimental approaches that have been taken to test the authors’ hypothesis is impressive. However, there are several important aspects that require clarification.

- We thank the reviewer for their exhaustive analysis of our manuscript and for proposing key experiments to test our hypothesis. We believe that this input has made a great positive impact on our manuscript.

1. The authors conclude that impaired cohesin loading cannot account for the defect in cohesin accumulation at stalled forks in the presence of HU after NIPBL depletion. This is based on the finding that depletion of NIPBL leads to a large reduction in EdU-SMC1 SIRF signal but only a minor reduction in the level of chromatin bound SMC1 (to 72.8 – 91.3 %).

To increase confidence in this conclusion, it would be important to repeat this chromatin-binding and stalled fork recruitment comparison ± NIPBL depletion when performing SIRF on a different core cohesin subunit, e.g. RAD21. This is especially important since RAD21 SIRF was performed in all the experiments which did not show a significant defect in SIRF recruitment following HU treatment ± auxin (i.e. the Sororin, WAPL and CTCF degron experiments), while SMC1 SIRF was not performed in these latter experiments. Such an experiment could also strengthen the authors’ conclusion that cohesion-related functions of cohesin are not important for its accumulation at stalled replication forks.

- We agree with the reviewer that the proposed experiments would complete our study and avoid any bias towards detection of a specific cohesin subunit in every case, helping to draw conclusions about the whole complex. As requested, we carried out Rad21 SIRF in NIPBL-degron cells and we included an SMC1 SIRF in Sororin-degron cells for completeness. As shown in the new Extended Data Figure 2h and the new Extended Data Figure 3d, depletion of NIPBL leads to a defective recruitment of RAD21 as previously observed for SMC1, with no strong impact on its chromatin bound levels (Extended Data Fig. 2i). On the other hand, Sororin depletion did not have any effect on the accumulation of SMC1 at stalled replication forks upon HU treatment, as previously observed with RAD21. See all included experiments below:

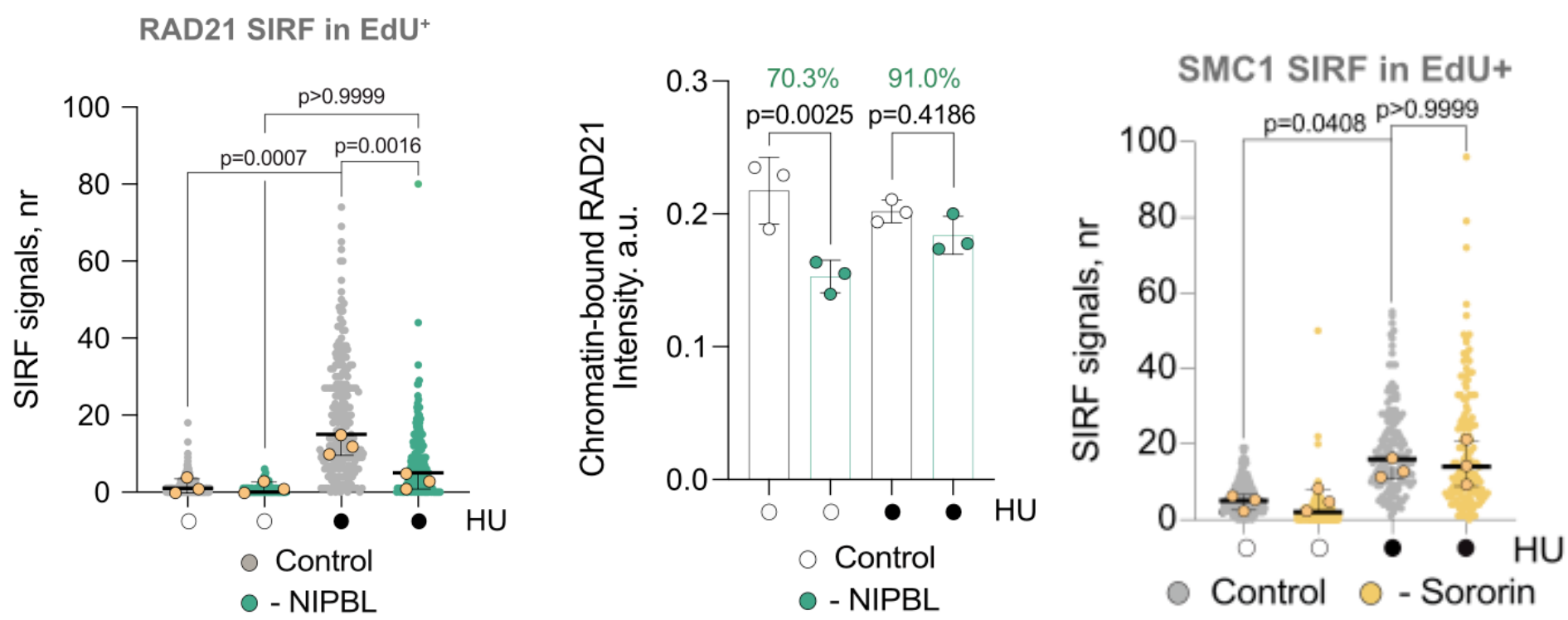

We have modified the text (Results section, page 4) to include these results:

“Finally, NIPBL depletion abolished cohesin SMC1 and RAD21 accumulation at stalled forks upon 2 mM HU treatment (Fig. 1g and h, and Extended Data Fig. 2h) with a marginal, non-significant increase of cohesin at stalled forks upon HU treatment in NIPBL-depleted cells, likely reflecting incomplete NIPBL depletion in our degron system. This cannot be explained by impaired cohesin loading in the absence of NIPBL, since cohesin levels on chromatin are ~75-90% of those in the presence of NIPBL (Extended Data Fig. 2g and i), consistent with previous reports[45,46].” .

"Since Sororin (CDCA5) was enriched at stalled forks in the iPOND-MS data (Fig. 1a), we tested whether the Sororin-mediated establishment of cohesion contributes to the observed accumulation of cohesin at forks. Using a degron strategy, validated by the loss of cohesion upon Sororin depletion (Extended Data Fig. S3a-c), we discarded this possibility for RAD21 and SMC1 subunits (Extended Data Fig. S3d)."

2. At higher doses and upon prolonged incubation times HU can induce replication fork collapse and lead to formation of DNA double strand breaks (DSBs). Can the authors exclude that HU induced the formation of DSBs in their experiments? This is an important point since DSBs have been reported to recruit cohesin and to function as sites at which one-sided cohesin mediated loop extrusion is initiated (Arnould et al., 2021; Teleoni et al., 2025). In the reported experiments, HU treatment could therefore also have altered cohesin's behaviour through DSB induction.

- We agree with the reviewer that this is an important point, in light of the latest studies about cohesin and DSB homology search in HR (PMID: 41343653, PMID: 41343630). Although a certain degree of DSB might be expected after prolonged, high HU treatments, 2mM 2h HU has not been reported to induce significant levels of DSBs (PMID: 20188668) and even less is expected upon 1h 100μM HU treatment. We observe cohesin accumulation at forks upon both treatments, essentially ruling out a role for DSB in this accumulation. To assess this important point directly in our system, we carried out immunofluorescence stainings with phospho-H2AX ser139 (γH2AX), i.e. a marker of DSB/replication stress, and phospho-ATM Thr1981 (pATM), i.e. a classical marker of DSB signaling, upon 2mM HU or 2 Gy γ-irradiation, used as a positive control. Foci count of both markers revealed that γ-irradiation induces a higher amount of pATM foci than 2mM HU treatments. Despite marked induction of γH2AX in S-phase cells in 2mM HU, we observed non-significant increase of pATM foci, suggesting only minimal levels of DSBs in these experimental conditions. We have included these data in the new Extended Data Figure 1i, and added a sentence in the text (Results section, page 3) discussing these results:

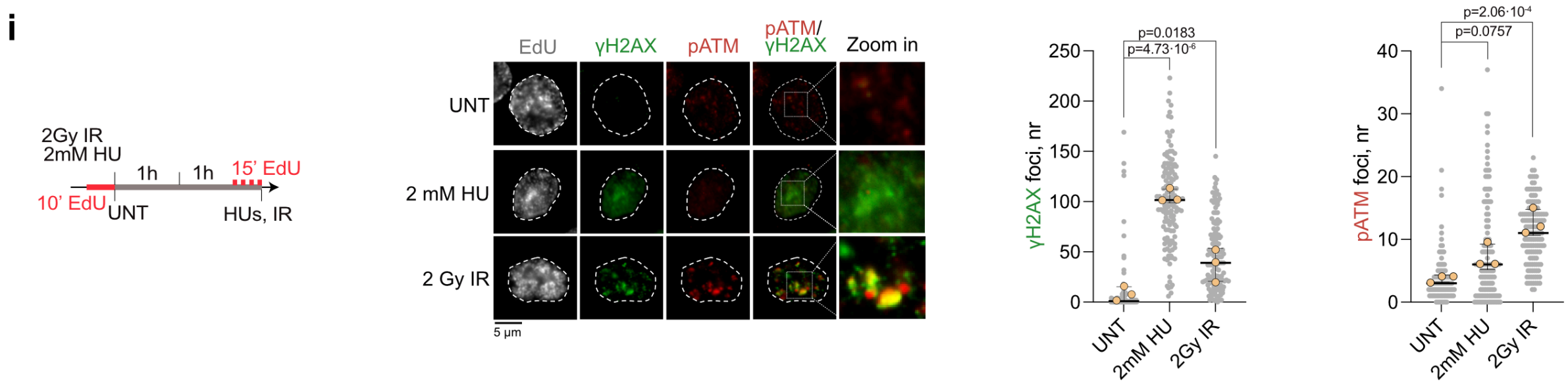

"We ruled out the possibility that double strand breaks (DSBs) were responsible for cohesin accumulation at forks by analyzing γH2AX (Replication stress or DSB marker) and pATM foci (DSB signaling marker) in S-phase cells treated with 2mM HU or γ-irradiation. While γH2AX was strongly induced upon 2mM HU, we observed a mild, non-significant increase for pATM. In contrast, both markers were significantly increased upon γ-irradiation (Extended Data Fig. 1i). This is in line with previous reports showing fork collapse only at prolonged times of high HU treatment[43]." (new ref. 43 PMID: 20188668) in the.

Related to this, why were different regimens of HU treatment used in different experiments (2 mM, 100 μM, 50 μM, 4 mM HU). It would be helpful to explain the rationale for this.

- This difference reflects the different effects of HU reported at each concentration and how those assays have been consistently performed in the literature.

A sentence was included already in the text for HU 2mM and 100μM and has now been modified for clarity following the request of Reviewer #1 in the Results section (page 3):

"…two different treatments with the ribonucleotide reductase (RNR) inhibitor Hydroxyurea (HU): 2 hours at 2 mM, which stalls all ongoing replication forks by severely depleting the cellular nucleotide pool[39]; and 1 hour at 100 μM, which induces fork slowdown[40] through mild reduction of cellular nucleotides[41] and by promoting TIMELESS dissociation at forks via reactive oxygen species (ROS) and disruption of oligomerized PRDX2[41].".

We have now included two additional sentences in the Results section (page 7), explaining why different HU concentrations (50μM and 4mM) were used in different experimental setups:

“Single molecule analysis of replication fork progression in the presence of 50 μM HU (which induces fork-reversal dependent slowdown via ROS, independently of nucleotide depletion), 200 nM MMC or 100 nM Aph, showed that these treatments reduced fork rate only in the presence of RAD21 (**Fig. 3a and b**).”.

“BRCA2 knock down in mAID-mClover-RAD21 resulted in nascent DNA degradation upon 3-hour 4 mM HU treatment (which impedes any DNA synthesis), as observed by the lower IdU/CldU ratio of single replication tracks (**Extended Data Fig. 8f**).”

2. The authors performed Sororin, CTCF and Wapl depletions and showed that these perturbations did not affect the accumulation of cohesin at stalled replication forks. Although these depletions appear to be efficient (Extended Data Fig. 3) there is no data in the manuscript to show that the depletions were sufficient to induce the previously published phenotypes of these depletion conditions (i.e. loss of sister chromatid cohesin upon Sororin depletion, loss of TADs and loops upon CTCF depletion and increase in interaction length and number and size of loops upon Wapl depletion). If the specific cell lines that have been used in this study have been previously published, these papers should be cited. If not, the above analyses should be performed and the results included.

- We agree with the reviewer that those experiments are important to confirm the effect of the depletions on cohesin functions. We assessed sister chromatid cohesion in metaphase spreads prepared after Sororin depletion and Micro-C experiments upon WAPL and CTCF depletions. As shown below and in the new Extended Data Figure 3c, g-i and m-o, upon acute Sororin depletion, 60% of metaphases cells displayed unpaired sister chromatids. Upon WAPL depletion, genomic contacts shifted to a higher range, decreasing TAD insulation and increasing loop length. Upon CTCF depletion the genome looks disorganized with no clear corner peaks, impaired TAD insulation and reduced number of loops. Hence, all depletions induced the expected and previously reported effects on cohesion or chromatin organization.

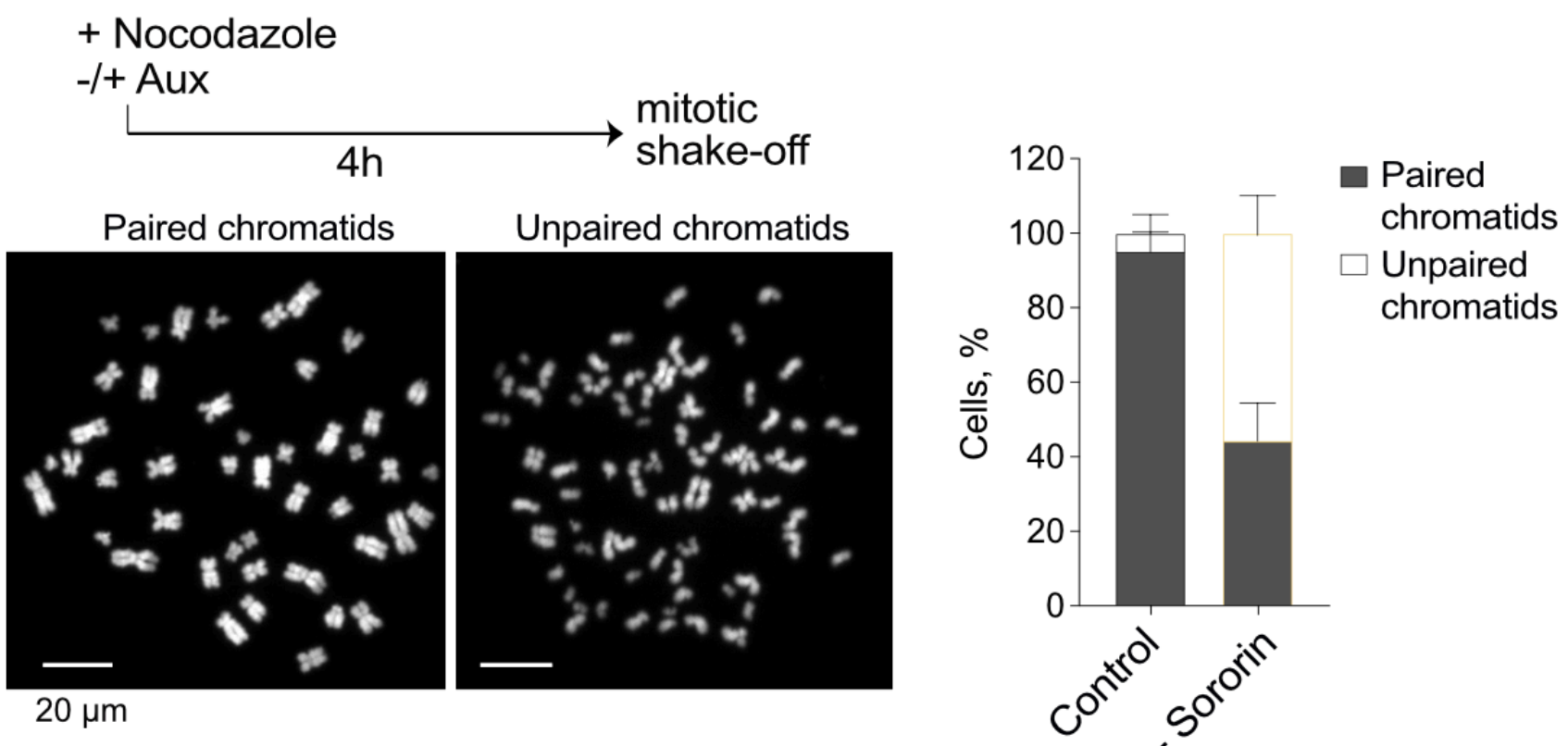

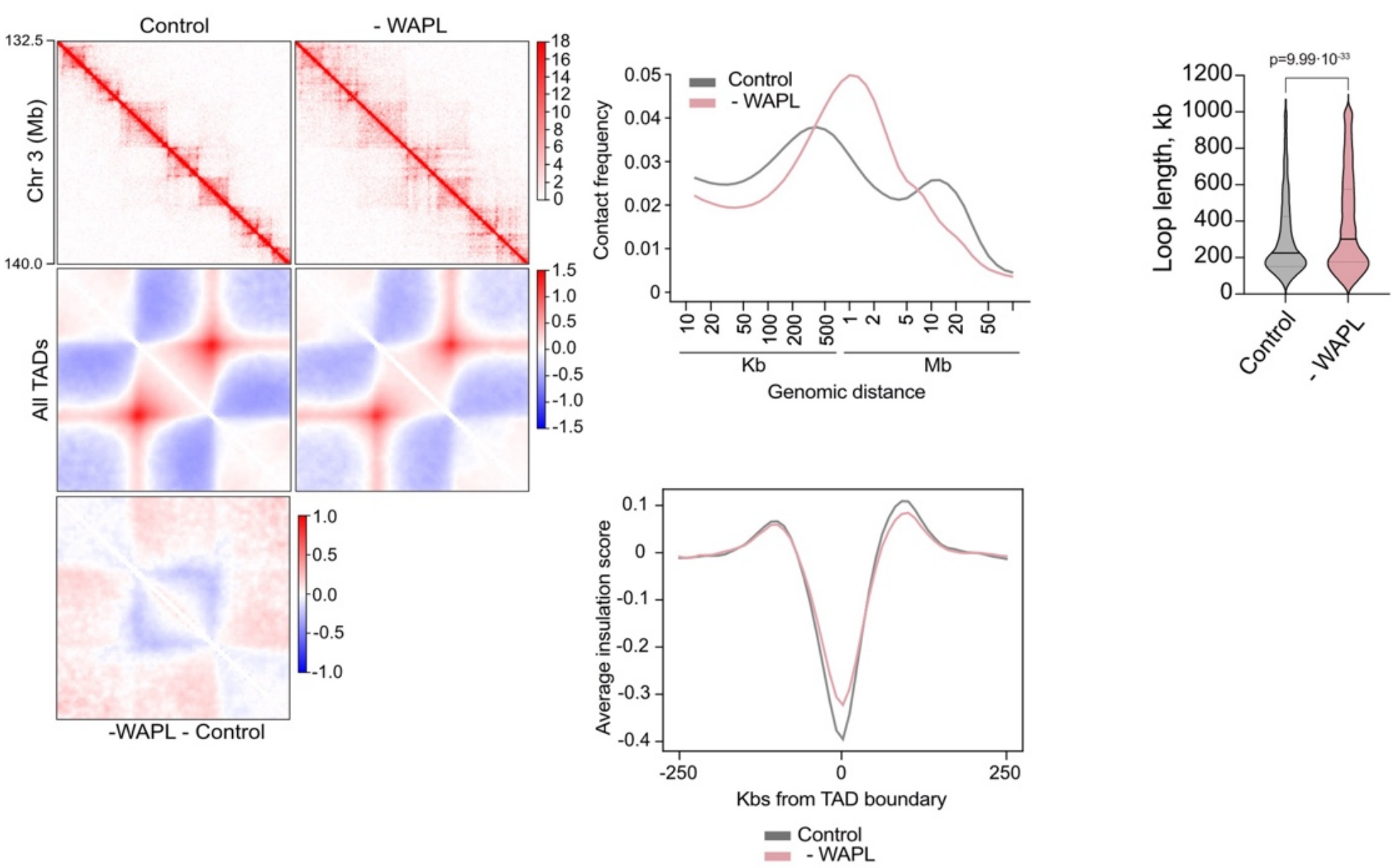

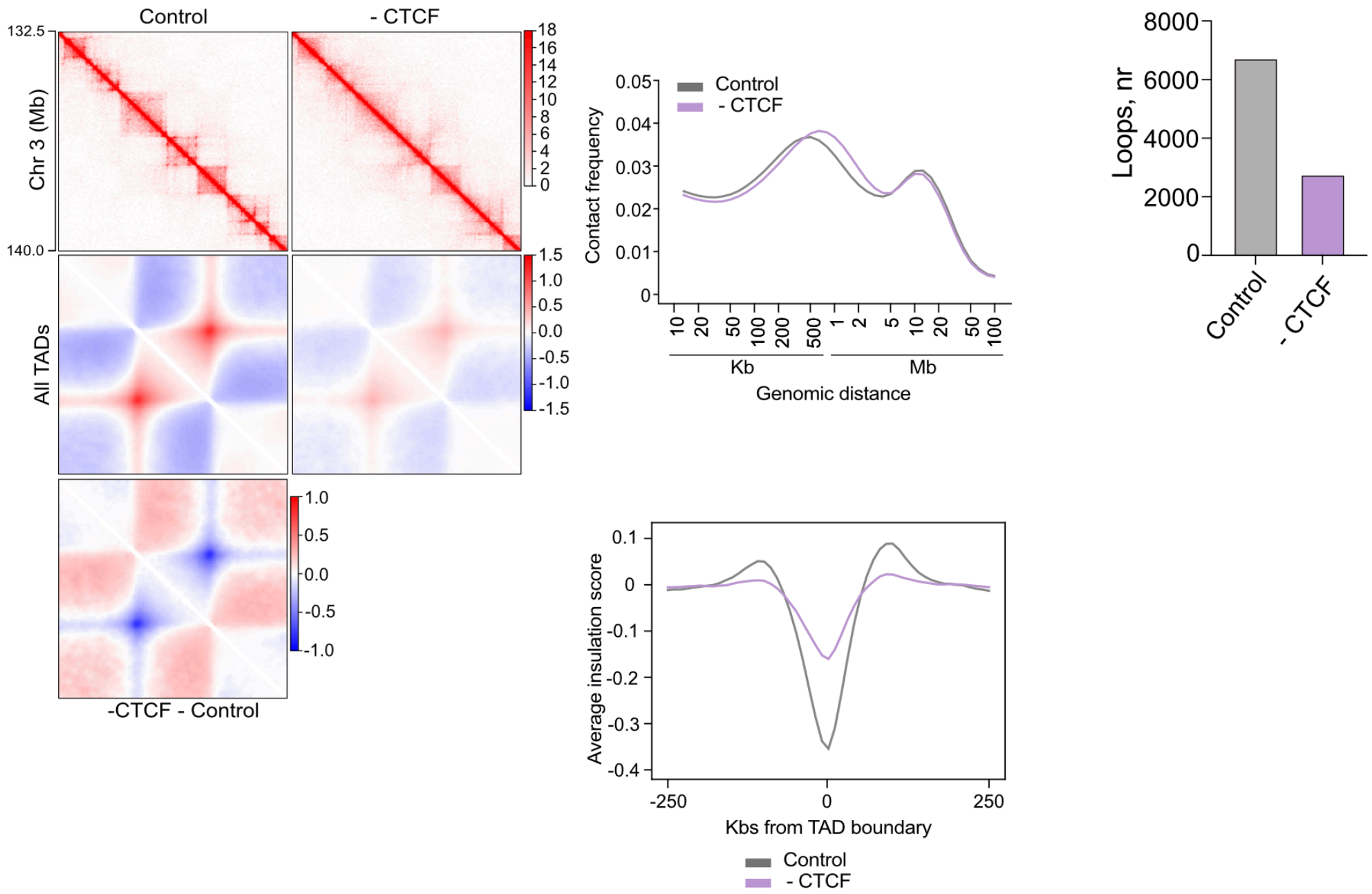

The following sentences have been included in the text in the Results section (page 4).:

“Since Sororin (CDCA5) was enriched at stalled forks in the iPOND-MS data (**Fig. 1a),** we tested whether the Sororin-mediated establishment of cohesion contributes to the observed accumulation of cohesin at forks. Using a degron strategy, validated by the loss of cohesion upon Sororin depletion (**Extended Data Fig. S3a-c**), we discarded this possibility for RAD21 and SMC1 subunits (**Extended Data Fig. S3d**).”

“Likewise, we could not find any effect of WAPL depletion on cohesin recruitment at stalled forks, even if WAPL depletion proved to shift genomic contacts to a longer range, decreasing TAD insulation and increasing loop length (**Extended Data Fig. 3e-j**).”

“…we analyzed cohesin recruitment at HU-stalled replication forks after CTCF elimination using a degron cell line previously generated[6,47]. As expected, CTCF depletion impaired TAD formation and weakened insulation scores[48]. We observed a non-significant trend towards decreased cohesin enrichment near forks in the absence of RS but no effect on cohesin recruitment to stalled forks (**Extended Data Fig. 3k-p**).”.

3. While the effect of HU on origin fountain structures as detected by Repli-C is detectable in aggregate genome wide analyses, the effect on the individual fountains shown in Fig. 2c is small and a complete comparison between Micro-C and Repli-C for the same genomic region in all conditions is missing. Could the authors show both Micro-C and Repli-C in the presence and absence of HU and SMC1 depletion for the same region, e.g. plot the same region as shown in Fig. 2b but for all conditions and not only wildtype?

- We thank the reviewer for raising this point and we agree that including these panels makes our figure more complete. We have included Micro-C maps of cells treated with HU in the presence and absence of SMC1 and plotted the difference Repli-C - Micro-C for all conditions in the new Figure 2b.

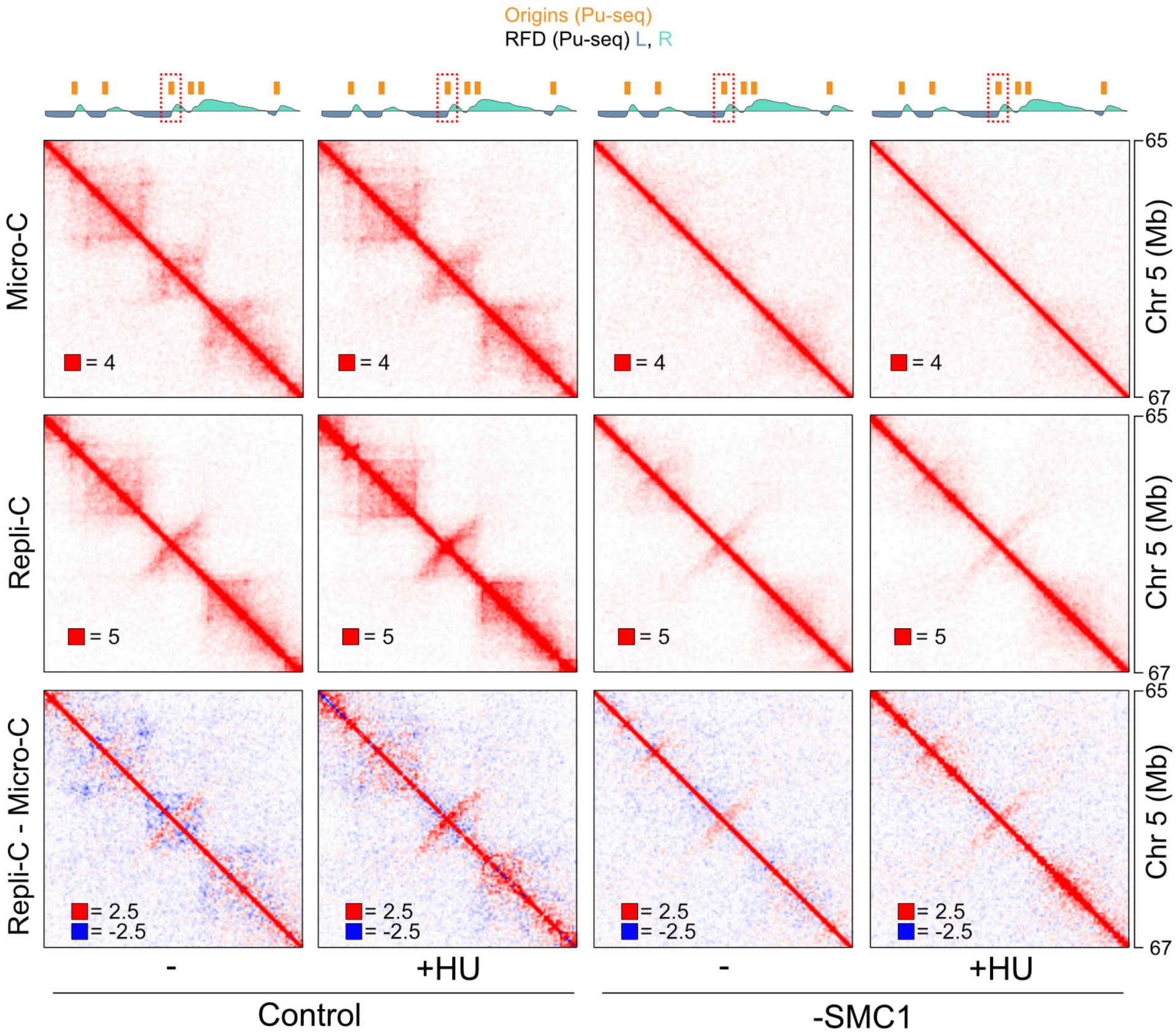

4. The authors have extensively studied the contribution of loop extruding cohesin versus cohesive cohesin on the accumulation of cohesin at stalled replication forks using the SIRF assay (NIPBL depletion, Sororin depletion, SMC1-3D expression, SMC1-4E expression). The authors have also identified replication origin fountains and inter-replisome contacts using Repli-C and have shown that these structures are affected to a greater or lesser extent following SMC1 depletion or SMC1-4E expression. Can the authors test whether Sororin depletion or SMC1-3D expression affects these structures as well? This would provide increased confidence that these structures are influenced by loop extrusion and not cohesive cohesin.

- We agree with the reviewer that this experiment would further support the conclusions derived from our extensive data ruling out a role for cohesive cohesin on the response to replication stress driven by loop extrusion. To this end we performed Repli-C in cells treated or not with HU in the presence and absence of Sororin.

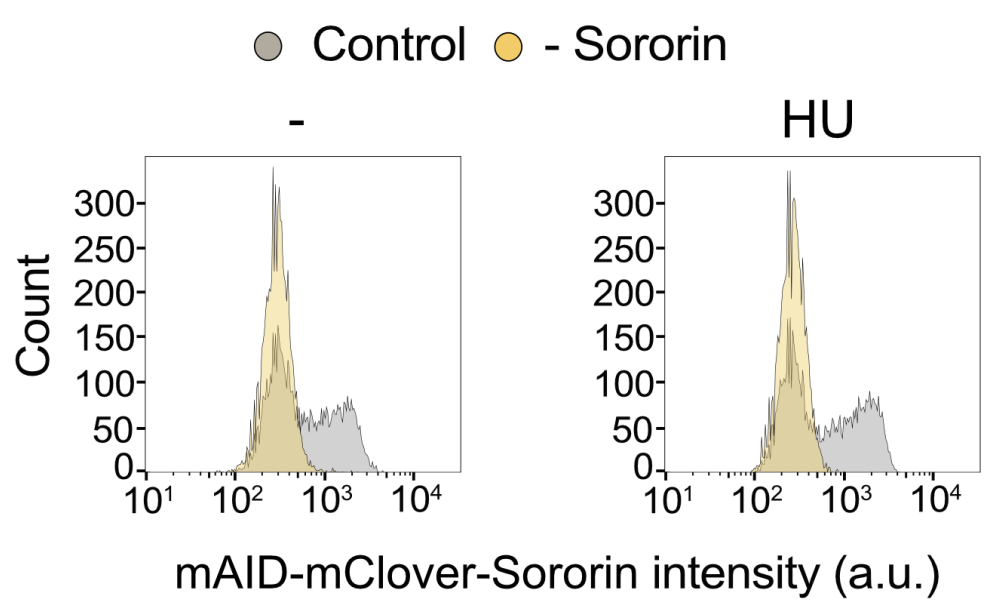

As shown below, (top panels), we observed enrichment of Repli-C contacts close to Pu-seq origin positions in untreated cells that were further enriched after HU treatment. Importantly, no differences were apparent between the conditions with and without Sororin. Of note, while the fountain-like structure could be detected in Repli-C aggregated plots when compared to Micro-C, it was less obvious than the structure observed in the control condition of the SMC1 degron cell line (from Figure 2d, show in the right column for comparison), reflecting some technical limitations in the new experiment.

A similar observation could be made for the inter-replicon contacts (lower panels). To improve the visualization of Repli-C-specific structures, we subtracted half of the aggregated signal of the corresponding Micro-C from the

Repli-C aggregate. Even after this correction, the enrichment in the Sororin experiment remained lower than the one obtained in the SMC1-depletion experiment (panels on the left). Despite these suboptimal conditions, we could still clearly observe an increase in inter-replicon contacts upon HU treatment, and this increase persisted in the absence of Sororin.

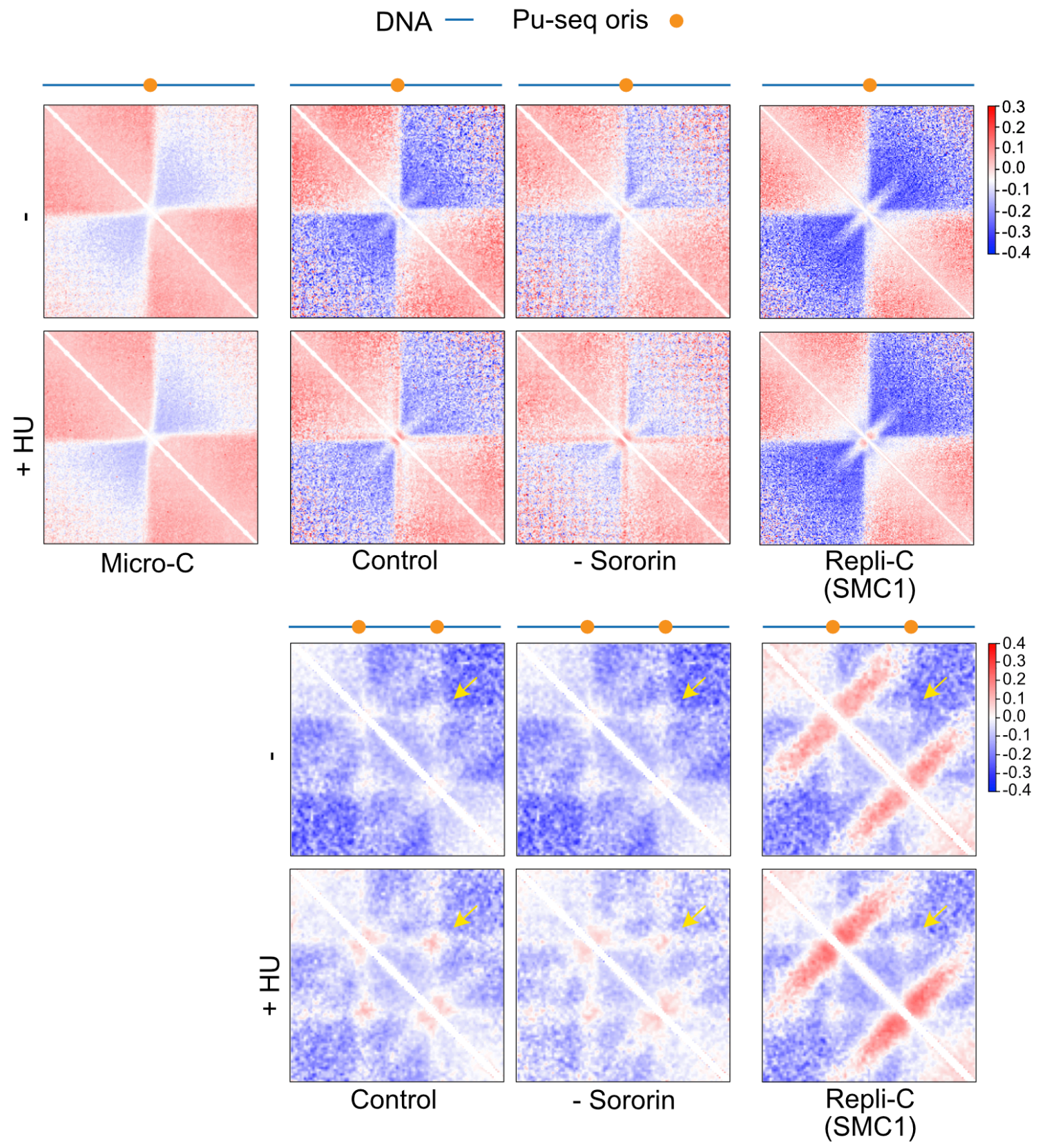

These results argue that Sororin is dispensable for intra- and inter-replicon contact formation upon HU, further supporting that only the loop-extrusion function of cohesin is required for this cellular response to replication stress. However, recognizing the suboptimal technical quality of this experiment, and our wish to keep the highest standards of data quality in the main manuscript, we would prefer to provide these results “for reviewers only”, and anyway accessible to the readers via the “peer-review” document published along with it.

We also want to mention that new data added as Extended Data Figure 9 in response to another request (point 6 below) also supports the lack of involvement of cohesion/Sororin in the observed phenotypes.

5. To support the hypothesis that the loop extrusion and not the cohesion function of cohesin contributes to replication fork reversal and slowing the authors used cohesin containing SMC1-3D and SMC1-4E as mutants that are selectively defective in cohesion and loop extrusion, respectively. However, the SMC1-3D mutant is not only defective in cohesion but has also been reported to have some defects in loop extrusion (Nagasaka et al., 2023). This raises the question whether the loop extrusion activities of these mutants really correlate with their accumulation at stalled replication forks and the formation of fountains and inter-replisome contacts. To test how good these correlations are it would be important to analyse the phenotypes of these mutants also in Micro-C experiments and to compare these data to the effects of these mutants at stalled replication forks.

- We agree with the reviewer that SMC1-3D mutant was shown to have some defects on loop extrusion, already tested in Hi-C experiments in the original report (PMID: 37591243), and this might impact as well cohesin’s role at forks. As it can be observed in Figure 2j and Figure 3d, the SMC1-3D mutant has an intermediate phenotype on cohesin accumulation at forks and fork slowdown upon HU treatment. We note that these effects are observed in a context where cohesin levels on chromatin (detected by Rad21 staining) for cells expressing the SMC1-3D mutant are comparable to those observed upon SMC1-WT expression. These results exclude that the phenotypes observed reflect problems on cohesin chromatin loading (**new Extended Data Figure 5f** and shown below), as already observed elsewhere and pointed out by the reviewer (PMID: 37591243).

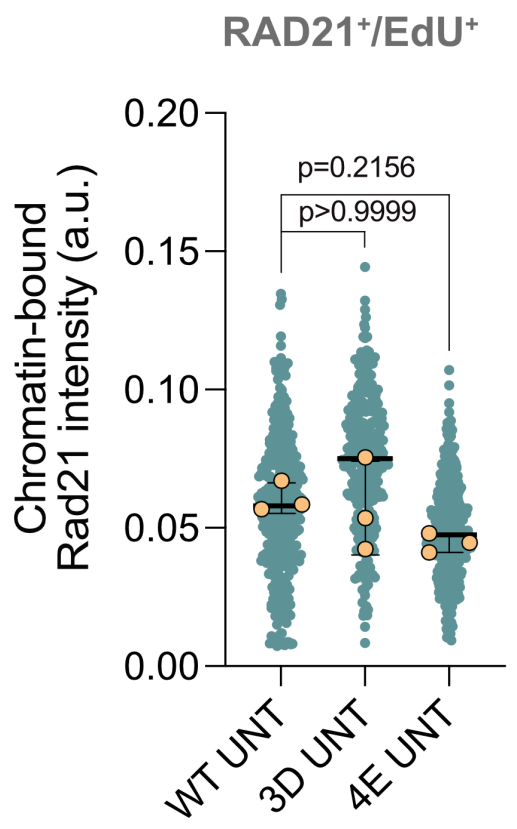

To clarify this, we have modified the following paragraphs in the Results section (page 6-7):

"In the $SMC1^{3D}$ mutant, three lysine residues in the hinge domain are replaced by aspartic acid and the resulting complex can sustain loop extrusion *in vitro* and shows a mild loop extrusion defect *in vivo* but is completely deficient in cohesion and cell proliferation[8] (**Fig. 2i**).".

"However, in SIRF assays with cohesin RAD21 subunit, $SMC1^{WT}$ and $SMC1^{3D}$ cohesin complexes significantly accumulate at forks upon HU treatment, while $SMC1^{4E}$ cohesin does not (**Fig. 2j**). The intermediate phenotype observed for $SMC1^{3D}$ is likely due to its incomplete loop extrusion activity *in vivo* since chromatin-bound levels in RAD21+/EdU+ cells are comparable to $SMC1^{WT}$ and $SMC1^{4E}$ (**Extended Data Fig. 5f**). These data confirm that cohesion is not required for cohesin recruitment at stalled forks."

"Accordingly, ectopic expression of $SMC1^{WT}$ and partly $SMC1^{3D}$, but not $SMC1^{4E}$, restore fork slowdown upon HU treatment when endogenous SMC1 is depleted (**Fig. 3d**). Unrestrained fork progression in $SMC1^{4E}$-expressing cells was also observed upon long-term substitutions (**Fig. 3e**).".

6. The authors' proposal that loop extrusion mediated fork slowdown upon HU treatment protects the forks from restart by PRIMPOL is based on rather complicated combinatorial depletions followed by DNA fibre analysis only and thus remains speculative. It would be useful to strengthen these conclusions experimentally. For example, the authors conclude that cohesin shields **DNA from PRIMPOL activity, but they do not show PRIMPOL accumulation when cohesin is depleted. iPOND experiments ± cohesin should be performed to support the claim directly.**

- We thank the reviewer for pointing this out and agree that showing whether the presence of PrimPol changes upon cohesin depletion would increase confidence in one of our key conclusions. However, attempts to detect PrimPol by iPOND have proved difficult in our hands. As an alternative to properly address this criticism, we have gone through extensive optimization of SIRF conditions, testing different PrimPol antibodies, EdU timings and fixation protocols. We have finally been able to detect increased PrimPol presence at HU-treated replication forks, an increase that was completely abolished upon PrimPol knock down by siRNA (new Extended Data Figure 9b), proving the specificity of the assay. Importantly, PrimPol SIRF upon HU was increased when SMC1 or NIPBL were depleted, but not in the absence of Sororin, further supporting that cohesin-mediated loop extrusion, not cohesion, protects nascent DNA from the excessive access of PrimPol (new Figure 4a, and Extended Data Figure 9c-d).

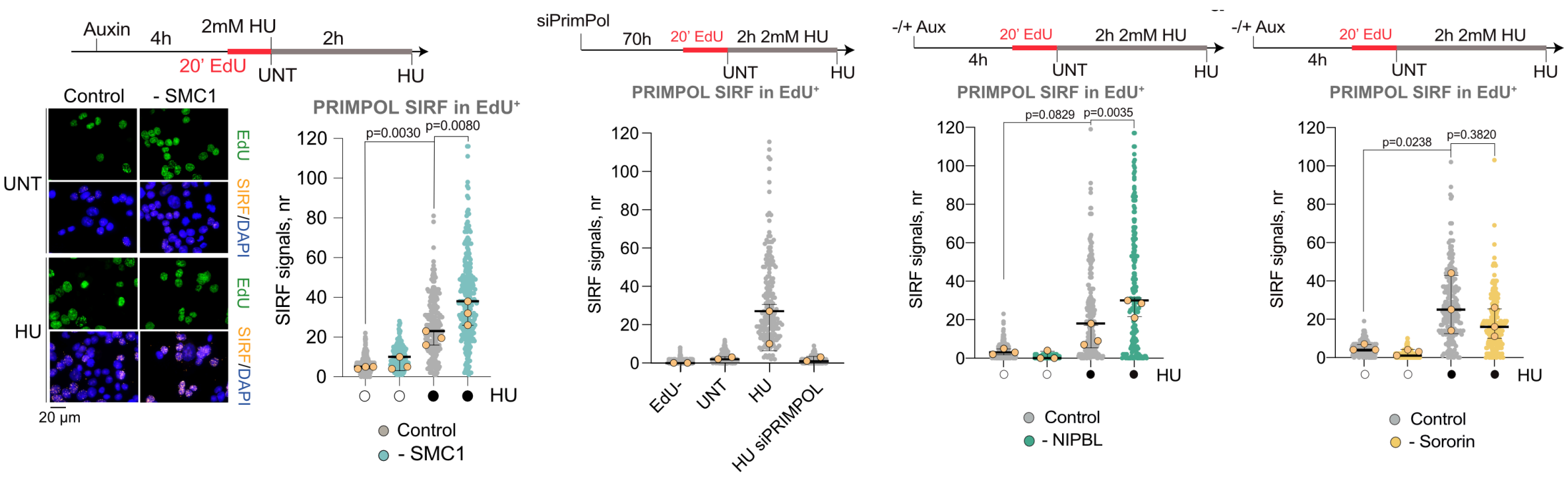

We have included the following text in the Results section (page 8). to discuss these experiments:

"IdU tracks were shortened by S1 treatment in HU-treated RAD21-depleted cells, pointing towards a discontinuous DNA synthesis (**Extended Data Fig. 9a**). The major contributor to discontinuous DNA synthesis upon fork reversal defects is the PRIMPOL primase[61]. To test the possible role of PRIMPOL we carried out PRIMPOL SIRF assays and showed that PRIMPOL increases at HU-stalled replication forks, an effect that is reversed by PRIMPOL downregulation (**Extended Data Fig. 9b**). Depletion of SMC1 or NIBPL, but not Sororin, further increased PRIMPOL levels at stalled replication forks (**Fig. 4a and Extended Data Fig. 9c and d**). To test the functional relevance of this increase, we downregulated PRIMPOL and observed that it prevented the unrestrained DNA synthesis of RAD21-depleted cells (**Fig. 4b and Extended Data Fig. 9e**), NIPBL-depleted cells (**Fig. 4c and Extended Data Fig. 9f**) and long-term substituted $SMC1^{4E}$-expressing cells (**Fig. 4d and Extended Data Fig. 9g**), clearly linking defective loop extrusion to deregulated PRIMPOL action."

Minor points

1. 'Length' spelt incorrectly in y axis Fig. 2d.

- This has been corrected.

2. Page 4, 'Moreover, SMC1 depletion drastically increased the fountain length, while HU consistently reduced it independently of cohesin'. The language should be toned down here. From the graphs presented in Fig. 2d, SMC1 depletion led to less than a ~ 1.5x increase in fountain length.

- We thank the reviewer for pointing this out. We have now toned down our phrasing in the Results section (page 5), which now reads as follows:

"Moreover, SMC1 depletion increased the fountain length, while HU consistently reduced it independently of cohesin".

3. SMC1 spelt incorrectly in Extended Data Fig. 6a upper right most panel.

- This has been corrected.

Cohesin reshapes replication fork contacts to aid fork slowing and reversal

González-Acosta Daniel[1, 5, *], Giménez-Llorente Daniel[2, 5], Rodrigues Melani[1], Aouami Moses[1], Barroso-Gomila Orhi[3], Muñoz Javier[3, 4], Cuadrado Ana[2], Losada Ana[2, *], Lopes Massimo[1, *]

Response to Referees' comments:

We thank all reviewers for their support and contributions to our manuscript throughout the revision process. Their comments and suggestions have considerably strengthened the manuscript and we are grateful for that.

****

Referee #1 (Remarks to the Author):

In my view, the authors have done an excellent job revising the manuscript and have addressed most, if not all, of the concerns raised by the three reviewers. They have added key experiments that strengthen their conclusions and made a substantial effort to improve the accessibility of the work for non-specialist readers, both in the main text and through new or improved graphical models. Regarding the issue of accessibility for non-specialized readers, I would be somewhat cautious about reviewer #2's point #1. While they are entirely right to emphasize that recent single-molecule studies suggest that fewer than 20% of initiation events occur within initiation zones identified by population-based approaches (Pu-seq, SNS-seq, OK-seq…), being too cautious in using Pu-seq data to interpret Repli-C results could be potentially confusing to readers. Since Repli-C is also a population-based assay, comparing the positions identified by these methods is fully appropriate.

We thank this reviewer for raising this point, on which we agree completely. In the final version of the manuscript – which needed to be shortened for editorial policy – the sentence discussing this point stands as follows: "Initiation of DNA replication takes place at DNA replication origins that spread along the genome[13,14] which are enriched in initiation zones (IZ), defined by population studies[15]".

****

Referee #2 (Remarks to the Author):

I am satisfied with the authors'answers to my comments.

We are grateful to this reviewer for his/her support to our revised manuscript.

****

Referee #3 (Remarks to the Author):

The authors have carefully revised their manuscript, in many cases by performing additional experiments and analyses. As a result, the authors have addressed and clearly answered almost all my comments and questions.

A single exception was my suggestion to perform Micro-C experiments with cells expressing the mutated cohesin subunits SMC1-4E and SMC1-3D to test directly if the effects of these mutations on fork restart really correlate with their effects on genome architecture. The purpose of these experiments would have been to obtain additional evidence that it is cohesin's loop extrusion and not its sister chromatid cohesion function in controlling replication fork slowdown upon HU treatement. However, the authors provide other lines of evidence in support of a loop extrusion not cohesion role of cohesin in this process. I therefore agree with the authors that these Micro-C experiments are not absolutely essential.

The revision has further strengthened this manuscript, which is impressive with respect to the amount and quality of data, resulting in a study that is exceptionally rigorous and advances both the genome architecture and replication stress fields conceptually in important ways. I would therefore now fully support publication of this interesting paper in Nature.

We thank Refereee #3 for recognizing that the specific role of loop extrusion (vs sister-chromatid cohesion) is supported by several lines of evidence in our manuscript. We are also grateful for the strong words of appreciation for our work and its improvement during revision.
